# New isolates from the 1970s to early 2000s provide insights into the evolution of Acinetobacter baumannii international clone 2 and its resistome

**DOI:** 10.1099/mgen.0.001752

**Published:** 2026-07-02

**Authors:** Matthew Neil, Frédéric Grenier, Nancy Allard, Gemma C. Langridge, Santiago Castillo-Ramírez, Louis-Patrick Haraoui, Benjamin A. Evans

**Affiliations:** 1 Norwich Medical School, University of East Anglia, Norwich, NR4 7UQ, UK; 2 Metabolic Health Research Department, Faculty of Medicine and Health Sciences, University of East Anglia, Norwich, NR4 7TJ, UK; 3 Centre for Microbial Interactions, Norwich Research Park, Norwich, NR4 7UG, UK; 4 Department of Biology, Faculty of Science, Université de Sherbrooke, Sherbrooke, Sherbrooke, Québec, Canada; 5 Department of Microbiology and Infectious Diseases, Université de Sherbrooke, Sherbrooke, QC J1K 2R1, Canada; 6 Centre de recherche Charles-Le Moyne, CISSS Montérégie-Centre, Greenfield Park, Québec, QC J4V 2H1, Canada; 7 Quadram Institute Bioscience, Rosalind Franklin Rd, Norwich Research Park, Norwich NR4 7UQ, UK; 8 Programa de Genómica Evolutiva, Centro de Ciencias Genómicas, Universidad Nacional Autónoma de México, Cuernavaca, Mexico; 9 Humans & the Microbiome Program, Canadian Institute for Advanced Research, Toronto, Ontario, Canada

**Keywords:** antimicrobial resistance, bacterial genomics, mobile genetic element, pathogen evolution

## Abstract

*Acinetobacter baumannii* remains a pervasive nosocomial pathogen, with international clone 2 (IC2) being the most successful lineage. IC2 is often discussed as a singular entity, and the genomic factors behind its success over other lineages are poorly characterized. This study aimed to track genomic changes occurring around the expansion of IC2 and further determine the within-clone population structure. A set of historical *A. baumannii* isolates from the 1970s to early 2000s was long-read sequenced and assembled into high-quality chromosomes. These were then used to supplement publicly available *A. baumannii* genomes, producing a dataset of 1,281 chromosomes derived from isolates collected across 6 continents. A recombination-free phylogeny was produced and combined with antimicrobial resistance gene presence/absence data. Analysis showed that IC2 became the dominant lineage around 2005, which coincides with the acquisition of *oxa23* and AbGRI3. Further, IC2 can be split into at least four distinct groups. Groups 1, 2 and 3 represent incremental evolution of the *A. baumannii* chromosome over time, while group 4 represents a separate evolutionary path distinct from the main IC2 lineage, which has recently been isolated at higher frequency.

**Impact Statement**

Characterizing the evolution and population structure of antimicrobial-resistant bacterial pathogens is of key importance both for epidemiological monitoring and for developing approaches to combat infections and their spread. Here, we use a unique collection of historical isolates to show that the major international clone in the World Health Organization (WHO) priority pathogen *Acinetobacter baumannii* is comprised of different, independent groups with individual antimicrobial resistance gene complements. This highlights new areas for research to understand the clinical impact of the different groups and the drivers underlying the evolution and spread of epidemic clones of antimicrobial-resistant bacterial pathogens.

## Data Summary

Sequencing data and assembled genomes for all isolates sequenced in this study are available on the Sequence Read Archive and GenBank, respectively, under BioProject PRJNA1404816. BioSample accessions for individual isolates can be found in Table S1.

## Introduction

Antimicrobial resistance (AMR) remains one of the most substantial causes of human morbidity and mortality worldwide, with *Acinetobacter baumannii* being particularly notable for its AMR properties. In 2019 alone, it was responsible for >400,000 AMR-related deaths, making it the fifth most lethal bacterial pathogen by this metric [1]. In addition to being a pervasive nosocomial pathogen, *A. baumannii* can be found in animals, plants, food products and even aquatic environments, making it a One Health issue [2]. Despite its current ubiquity, *A. baumannii* is thought to have emerged relatively recently, likely around the 1970s [3,4]. During this early expansion, international clone 1 (IC1) and international clone 2 (IC2) lineages were the dominant form of *A. baumannii*. Then, IC2 began to overtake IC1 as the dominant lineage and currently accounts for the majority of *A. baumannii* infections worldwide, followed by IC5 [5]. However, the lack of sequencing data from historical isolates means that identifying the genomic changes underlying the success of IC2 over IC1 is challenging to determine with existing data.

The *A. baumannii* genome is characterized by a small core genome and a large, diverse accessory genome containing an array of antimicrobial resistance genes (ARGs) and virulence factors (VFs) that tend to be concentrated on the chromosome [6,8]. Intrinsic ARGs in *A. baumannii* encode class C and class D *β*-lactamases, referred to as *Acinetobacter*-derived cephalosporinase (ADC) and *Acinetobacter baumannii* oxacillinase (OxaAb, also referred to as OXA-51-like) enzymes, respectively. Additionally, the *A. baumannii* chromosome contains clusters of genes involved in the biosynthesis of extracellular capsular polysaccharides and lipooligosaccharides, known as the K-locus and OC-locus. These play a major role in virulence and resistance to both environmental stresses and antibiotics [9,10]. *A. baumannii* chromosomes also contain a vast array of acquired ARGs. Two of the most clinically relevant are *oxa23* (also referred to as *bla_oxa-23_*), conferring high-level carbapenem resistance [11], and *armA*, which confers resistance to a broad range of aminoglycosides [12,13]. Due to the high genome plasticity of *A. baumannii*, closely related isolates may have very different ARGs, leading to potentially different antimicrobial susceptibility patterns.

Many ARGs in *A. baumannii* are co-localized in resistance islands (RIs), of which there are two main types: AbaR-type RIs are associated with IC1, while AbGRI-type RIs are associated with IC2 [14]. Of the AbGRI-type RIs, AbGRI3 is perhaps the most clinically relevant due to the presence of *armA*, which is found exclusively within AbGRI3 in IC2 isolates [15]. AbGRI3 contains three main sections: a core containing *armA*, *mphE* and *msrE*; a large integron containing *sul1*, *qacEΔ1*, *aadA*, *catB8* and *aac(6*′)*-Ib9* (also referred to as *aacA4*), which together with the core region forms Tn*6180*; and an additional transposon (Tn*6179*) containing *aph(3*′)*-Ia* (also referred to as *aphA1b*). At least five variants of AbGRI3 exist based on the presence/absence of these sections and the deletion of a replication initiation protein in the core section and/or a class 1 integron integrase in the integron section [15]. However, for simplicity, here we define ‘short’ AbGRI3 variants as those which only contain the core section (version 4 in Blackwell *et al*. [15]) and ‘long’ AbGRI3 variants as those which contain additional sections.

Within the core genome, *A. baumannii* displays low genetic variance due to high levels of recombination and recent, rapid clonal expansion [16]. As core-genome alignments with recombination not removed are commonly used for constructing *A. baumannii* phylogenies, subgroups within ICs including IC2 are not routinely identified. Nonetheless, an approach using whole-genome recombination-free alignments, which include much greater genetic variance, has previously been applied to IC1 to track its emergence and the genomic factors behind its success [17]. A recent paper by Li *et al*. [18] similarly found subgroups within IC2. However, they did not fully characterize the genome content of each group with respect to AMR, even though isolates separated by little evolutionary distance in the core genome can have considerable variation in their accessory genome, which includes ARGs [19]. Therefore, it is unclear which ARGs may be associated with each group and what potential variation between insertion sequence (IS)/RI content exists between groups.

To gain a better understanding of the emergence and structure of IC2 over time, we sequenced a set of historical *A. baumannii* genomes. These were combined with publicly available sequences to produce a comprehensive dataset of *A. baumannii* chromosomes collected across six continents and five decades. By integrating ARG presence/absence with a recombination-free phylogeny constructed from a whole-genome alignment, we show that IC2 can be split into at least four distinct groups. Three of these groups reflect a stepwise acquisition and loss of certain ARGs over time, associated with changes in the RI AbGRI3. The fourth group is distinct from the other three, representing a parallel evolutionary path within the *A. baumannii* IC2 major lineage.

## Methods

### Historical isolates sequencing

Following overnight growth at 37 °C in Miller’s lysogeny broth, genomic DNA was extracted using the Quick-DNA Magbead Plus Kit (Zymo Research). DNA was purified further using Ampure XP beads (Beckman Coulter). DNA libraries were prepared using the Rapid Barcoding Kit 96 V14 from Oxford Nanopore Technologies (ONT). Sequencing was done with an R10.4.1 PromethION Flow Cell using a PromethION 2 Solo. Base calling and adaptor trimming were performed using Dorado v0.7.2 with the dna_r10.4.1_e8.2_400bps_sup@v5.0.0 model (ONT, Oxford, UK). Reads under 1 Kbp or below Q10 were filtered out using Filtlong v0.2.1 [20]. Samples with predicted coverage <30× were removed, and samples with a predicted coverage >80× were downsampled using Filtlong. Read length was prioritized over read quality by setting length weight to 10 and leaving quality weight at the default of 1.

### Historical chromosome assembly and quality control

All computational analysis was performed on a high-performance computing cluster running Rocky Linux v8.10. Reads were assembled using Flye v2.9.5-b1801 [21] with the nano raw model. Target coverage was set to 50× with an estimated genome size of 3.9 Mbp. A single round of polishing was performed with the in-built polisher. Next, one round of Racon v1.5.0 [22] and one round of Medaka v2.0.1 (Oxford Nanopore Technologies, 2018) polishing were applied to all assemblies, with Minimap v2.28-r1209 used as the aligner. Finally, ReCycled v0.1.0 [23] was used to circularize and start all contigs at the *dnaA* gene, with a small subset of 20 contigs where this process failed being kept.

Benchmarking universal single-copy orthologues (BUSCO) scores, which assess the completeness of the annotations by identifying a set of highly conserved single-copy orthologs, were obtained for the longest contig in each assembly using BUSCO v5.8.3 [24]. Kraken v2.1.4 [25] was used to classify each contig. Only contigs classified as *A. baumannii*, >3.5 Mbp and <4.5 Mbp in length, with GC content >36% and <43%, containing at least one origin as detected by ReCycled, and with BUSCO scores >95%, were considered whole chromosomes. Average nucleotide identity (ANI), which evaluates the genetic relatedness of two genomes, against the *A. baumannii* type strain (ATCC 19606, GenBank accession no. CP045110.1), was checked using FastANI v1.34 [26], with all isolates having >95% ANI.

### Publicly available genome retrieval and metadata collation

Publicly available *A. baumannii* genomes marked as ‘chromosome’ or ‘complete genome’ were retrieved using the National Center for Biotechnology Information (NCBI) datasets utility [27] (accessed on 24 May 2025). Genomes marked as atypical or suppressed were removed. For each genome, the longest contig was extracted, and any contigs shorter than 3 Mbp, longer than 5 Mbp or not containing a *dnaA* gene were removed. This resulted in a total of 1,055 publicly available chromosomes, which were used to supplement the 226 historical chromosomes. For all isolates, metadata were processed to obtain the collection year and country. In 84 historical isolates (but no publicly available isolates), a date range was given, and an average was taken in these cases.

### Chromosome annotation

Chromosomes were annotated with bakta v1.11 [28] using the light database. ARGs were annotated with the resistance gene identifier (RGI) v6.0.4, which queries the Comprehensive Antibiotic Resistance Database (CARD) [29]. VFs were annotated using ABRicate v1.0.1 [30], which queries the virulence factor database [31]. ISs were annotated using digIS v1.2 [32]. Integron features were annotated using IntegronFinder v2.0.6 with genome topology set to circular and local max set to true for increased sensitivity [33].

### Core genome phylogeny

Panaroo v1.5.2 [34] was run on GFF3 files output by Bakta to create a core genome alignment with MAFFT [35] as the aligner and a core genome threshold of 1. IQ-TREE v2.4.0 [36] was run on this alignment with the substitution model determined using ModelFinder [37]. The core genome phylogeny was rooted with an *Acinetobacter nosocomialis* outgroup (GenBank accession no. CP157432.1).

### Multi-locus sequence typing and IC assignment

*In silico* multi-locus sequence typing (MLST) was performed on all genomes using FastMLST v0.0.16 [38] using the Pasteur scheme. Clonal complexes were constructed at the single locus variant level using the goeBURST algorithm [39] implemented in PHYLOViZ [40]. The founder sequence type (ST) and other associated STs for each IC were obtained from previously published data [4,41, 42]. Isolates that were both assigned to the same clonal complex and monophyletic group within the core genome phylogeny as an isolate of a founder ST were assigned to the corresponding IC.

### Recombination-free IC2 phylogeny

Whole-genome alignment of chromosome sequences was performed using ska2 v0.4.0 [43] against a recent ST2 isolate (GenBank accession no. CP181411.1). Gubbins v3.4 [44] was used to generate a recombination-masked alignment using five iterations. The minimum SNPs to identify a recombinant region was lowered from the default 3 to 2 for aggressive removal of recombination. For efficiency, Gubbins allows for a different (typically simpler and faster) substitution model and tree builder to be used on the first iteration compared to subsequent iterations. Here, the first iteration substitution model was set to Jukes–Cantor, and the first iteration tree builder was set to FastTree. On subsequent iterations, IQ-TREE was selected as the tree builder, and the best substitution model was automatically calculated on each iteration by ModelFinder [37], which is run as part of IQ-TREE. Marginal ancestral state reconstruction was used with RAxML as the model fitter. The substitution model used during ancestral state reconstruction was GTR, as in previous runs, ModelFinder had identified this as the best substitution model for tree building. To obtain support values, the Gubbins bootstrap argument, which controls the number of bootstrap replicates performed by the tree builder, was set to 1,000. As the tree builder was set to IQ-TREE, these replicates were performed by UFBoot2 [45], which is invoked by IQ-TREE. The ATCC 19606 genome described above was used as an outgroup and was pruned from the final tree using Newick utilities v1.6 [46]. Visualization was performed using TreeViewer [47].

### Temporal and other statistical analysis

Gene enrichment/depletion in both ICs and IC2 subgroups was determined based on ARG annotations produced by RGI with Fisher’s exact test using Scipy v1.13.1 [48]. Root-to-tip analysis was performed using BactDating v1.1.3 [49] in R v4.3.1. Nodes without dates were pruned before analysis using ape v5.8–1 [50]. Mann–Whitney U test was performed to determine if distributions of total features (ARGs, VFs, ISs and ISA*ba1*) were significantly different between groups. To quantify the size of this difference, the effect size was calculated with Cliff’s delta [51]. All *P* values were adjusted for multiple testing using the Holm–Bonferroni correction with statsmodels v0.14.4 [52], with *P*<0.05 deemed statistically significant.

### ADC variant analysis

Amino acid sequences for ADC-175, ADC-25, ADC-30, ADC-73 and ADC-33 were obtained from the CARD (CARD accession no. WP_001211208.1, ABK34773.1, AEL30572.1, ALA14808.1 and WP_001211220.1 respectively). Alignment was performed using muscle v5.3 [53]. Phylogeny was inferred with IQ-TREE v3.0.1 [36] with the substitution model determined using ModelFinder [37]. Network analysis was performed using NetworkX v3.1 [54].

## Results

### Historical *A. baumannii* chromosomes suggest the expansion of IC2 began in 2005

To analyse the changes in the *A. baumannii* chromosome over time, a set of 226 historical *A. baumannii* isolates from the 1970s to early 2000s was collated. All isolates were sequenced using Oxford Nanopore Technology and assembled into complete chromosomes. All chromosomes showed BUSCO scores >95% and ANI >95% when compared to the reference strain ATCC 19606 (Table S1, available in the online Supplementary Material). This set of isolates was then combined with publicly available *A. baumannii* genomes at the ‘complete’ or ‘chromosome’ level to create a dataset of 1,281 chromosomes (Tables S1 and S2). The majority of isolates were collected from the late 1970s to the present day, although seven chromosomes were from isolates collected before 1970. Of the 183 isolates sequenced in this study with a known country of origin, most were from Europe (45%), the UK (15%) and Saudi Arabia (15%), with smaller contributions from countries such as Türkiye (9%), Singapore (6%) and Croatia (5%). Meanwhile, the majority of isolates collected after 2010 were from the USA and China, reflecting the global trends in strain isolation and genome sequencing.

**Fig. 1.**
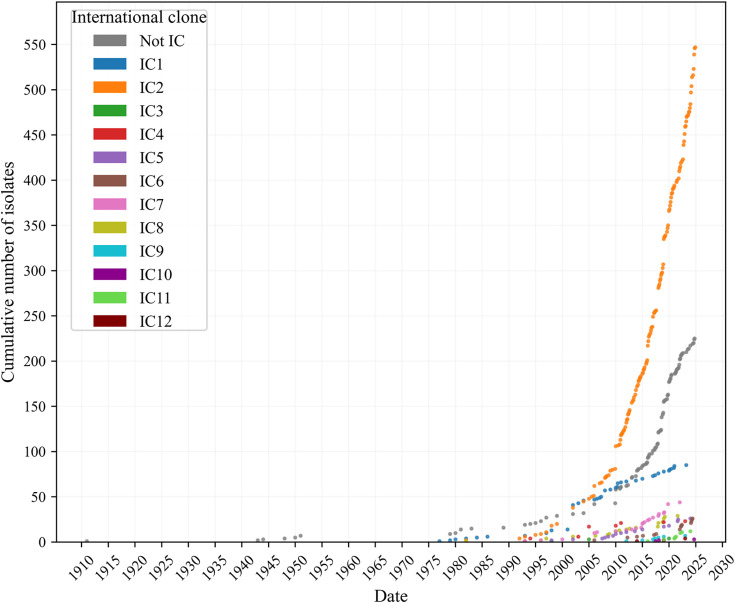
Cumulative number of isolates over time. Each dot represents a single isolate. Dots are coloured according to the assigned IC, with isolates not belonging to any IC shown in grey.

All isolates were assigned to ICs using MLST according to the Pasteur scheme. As expected, IC2 was the largest, with just over half (50.9%, 628 isolates) of all isolates assigned a ST belonging to IC2. Of these, 68 were from historical isolates sequenced for this study (Table S1), and 560 were publicly available genomes (Table S2). During inspection of the core genome phylogeny, it was found that one IC2 isolate (Genome ID: Chr_733_1 in Table S1) was not grouped with other IC2 isolates and formed a distinct outgroup, so it was removed from downstream analyses. The first IC2 isolate in this dataset was collected in 1982. However, it was not until the early 2000s (~2005) that IC2 overtook IC1 to become the dominant clone (Fig. 1). Since then, IC2 has shown rapid growth, while IC1 has shown far less growth. Therefore, it is probable that any genomic changes that underlie IC2’s success would have arisen around the early 2000s.

### IC2 isolates are enriched in a diverse set of ARGs

**Fig. 2.**
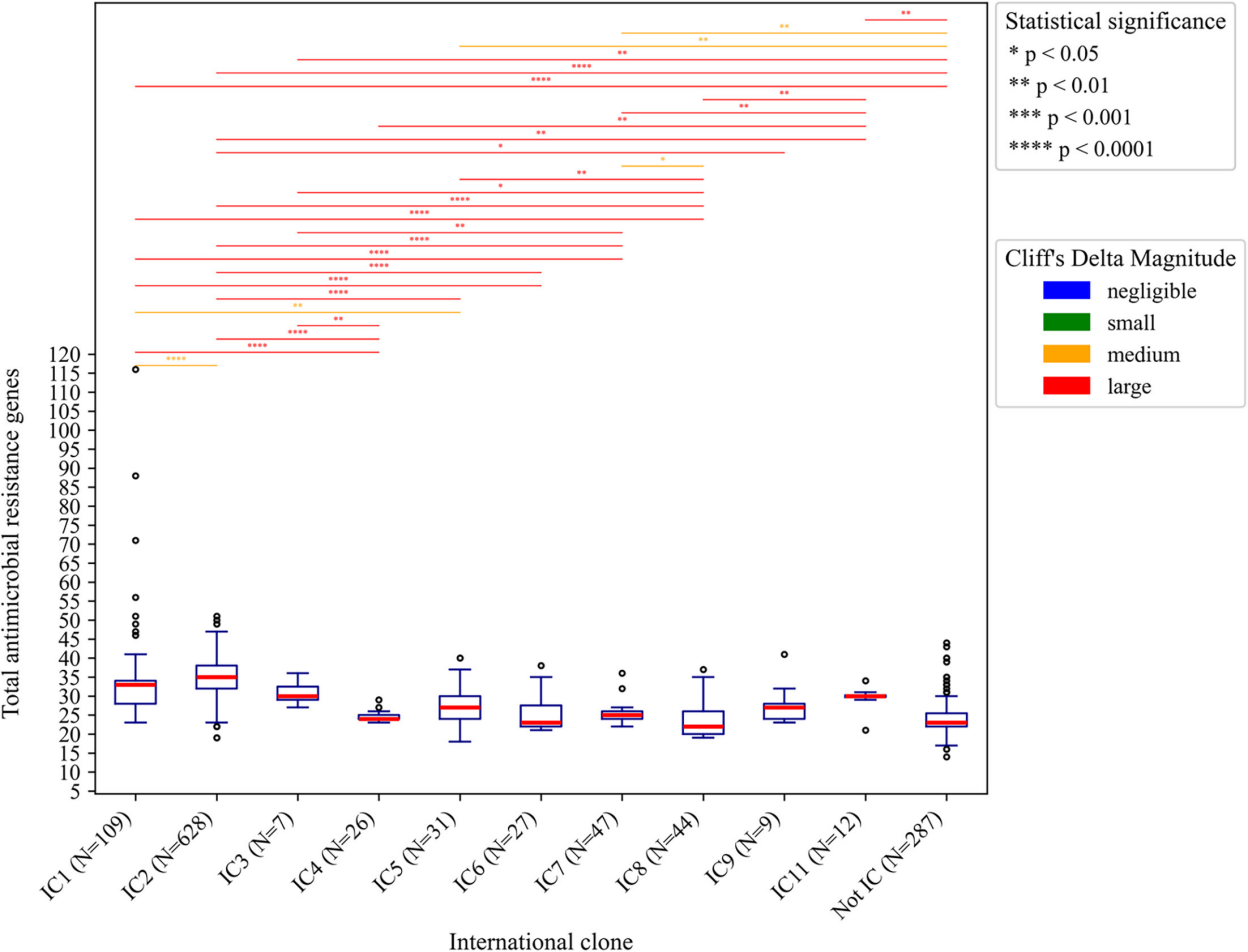
Boxplots of total ARGs on *A. baumannii* chromosomes in the different ICs. IC10 and IC12 have been omitted due to being represented by fewer than five isolates in our dataset. Medians are shown with red lines. Outliers (±1.5×IQR) are shown as open circles. Statistical significance (*P*<0.05) was determined using the Mann–Whitney U test with the Holm–Bonferroni correction applied. Cliff’s delta was used to determine the effect size.

**Fig. 3.**
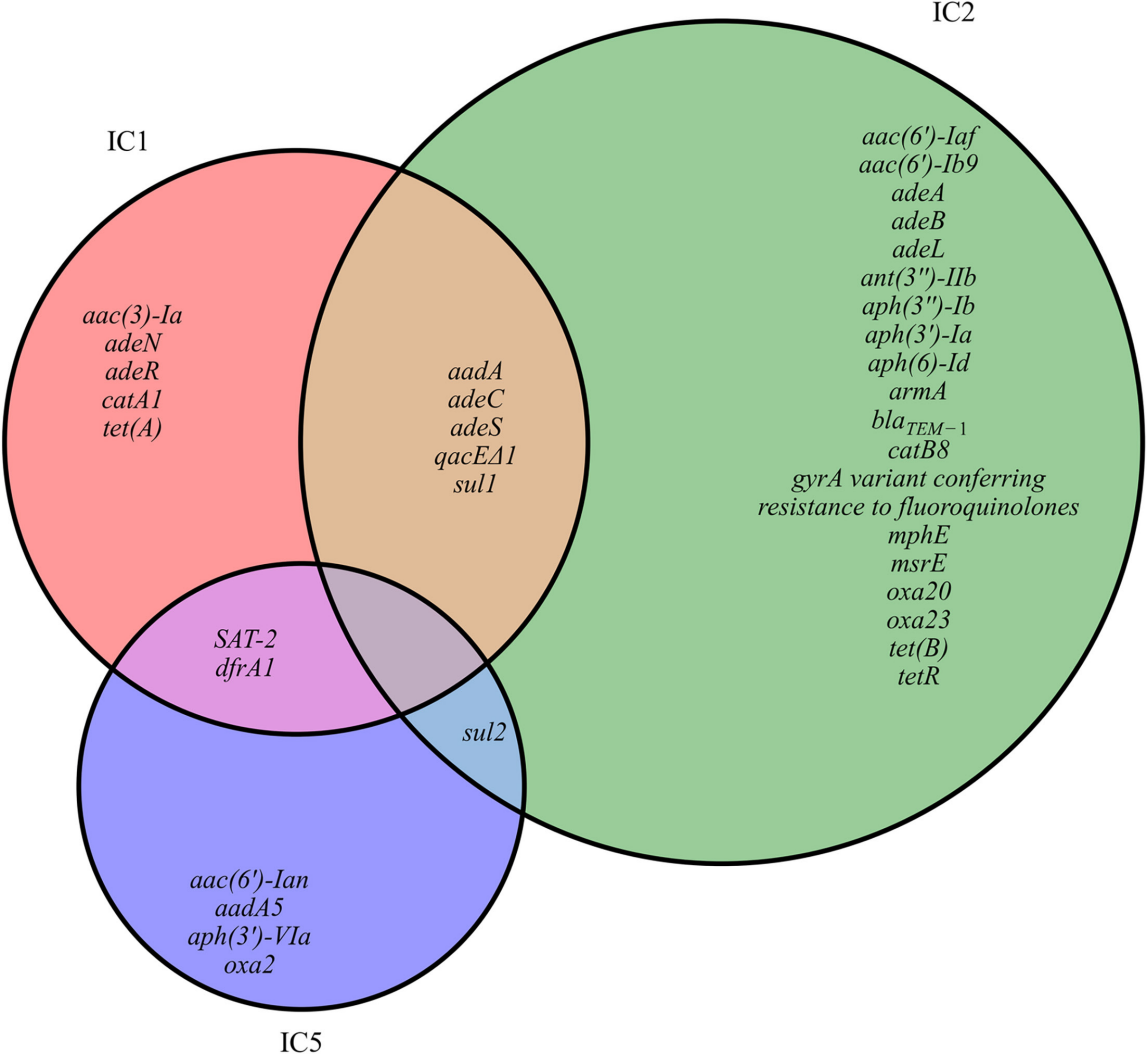
Enrichment of ARGs in IC1, IC2 and IC5. ARGs were annotated using RGI. Statistically significant (*P*<0.05) enrichment of ARGs in each IC was determined using Fisher’s exact test with the Holm–Bonferroni correction applied. Results from the three most globally dominant ICs (IC1, IC2 and IC5) are shown, with other ICs omitted. The intrinsic *bla*_ADC_ and *oxaAb* genes have been omitted.

To further investigate the genomic changes in IC2 that may have contributed to its success, ARGs were annotated using the RGI, which queries the CARD. The highest number of ARGs per chromosome was found in a set of three IC1 isolates with a large gene amplification [55] (GenBank accession no. CP090607.1, CP090606.1, CP091172.1). Beyond these three isolates, IC2 isolates were found to have a significantly higher number of ARGs on their chromosomes than all other ICs and those not belonging to an IC (Fig. 2). Further, a large set of ARGs was significantly enriched in IC2 (Fig. 3), many of which were either unique to this clone or were only sparsely found outside of it. These ARGs confer resistance to a wide array of antimicrobials, including carbapenems, aminoglycosides, sulphonamides, fluoroquinolones, macrolides and tetracyclines, as well as certain disinfecting agents. This, combined with the higher median number of chromosomally encoded ARGs in IC2 isolates over other IC/not IC isolates, suggests that IC2’s success may have been driven by resistance to a variety of antimicrobials rather than to a single antimicrobial.

### IC2 can be split into at least four groups with corresponding ARG presence/absence patterns

**Fig. 4.**
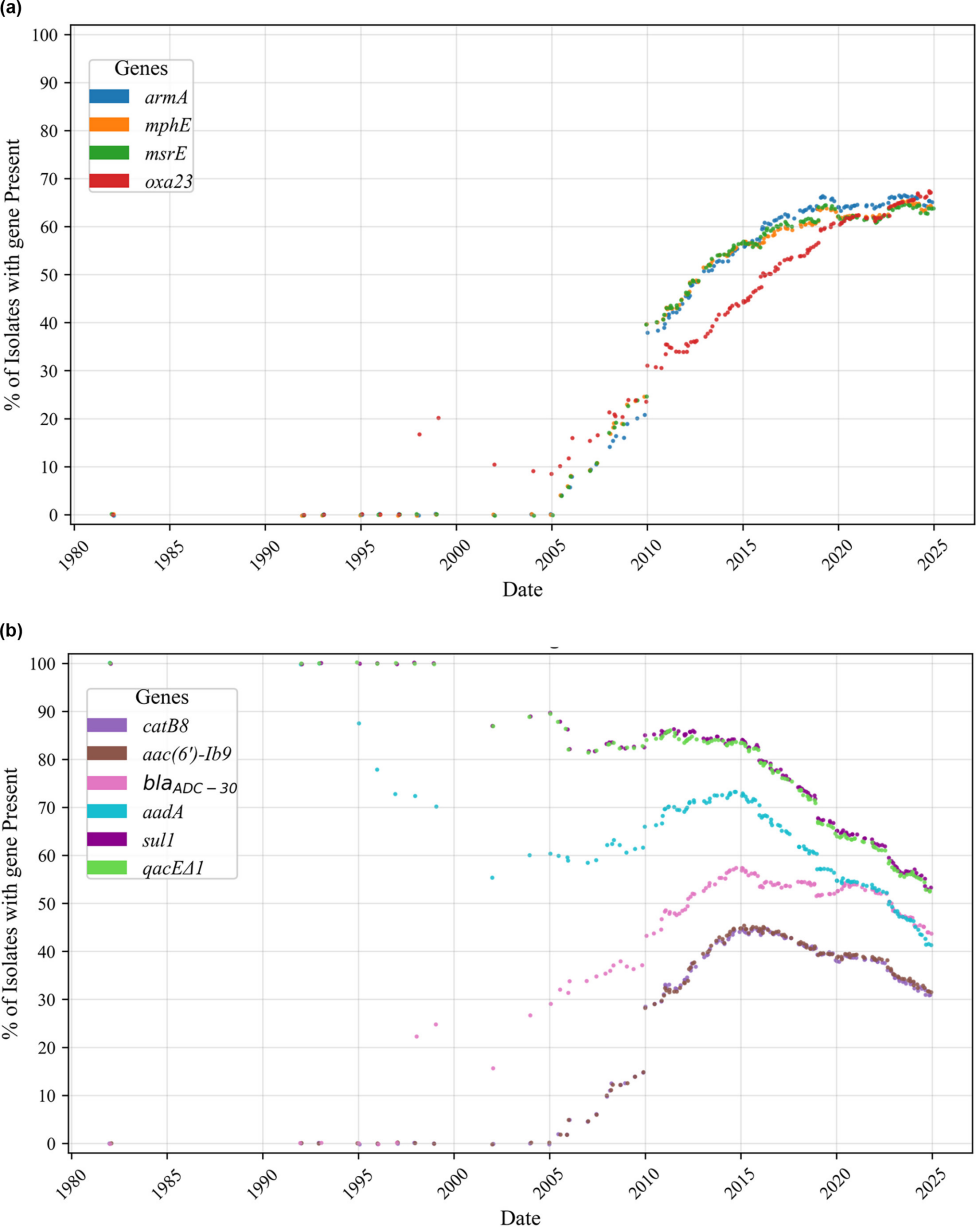
Proportion of IC2 isolates containing ARGs over time. Each dot represents the percentage of IC2 isolates (*N*=627) up to and including that date, which contain at least one chromosomal copy of the corresponding ARG. (**a**) and (**b**) show different sets of ARGs with differing presence/absence patterns over time. In (**a**), percentage values plateau towards a maximum value due to the inclusion of older isolates, which lack the ARGs shown. Dots are coloured according to a corresponding ARG.

Following the previous analysis of ARG enrichment in IC2, the proportions of IC2 isolates containing ARGs over time were investigated. Two patterns were noted amongst these ARGs. A subset of ARGs (*armA*, *mphE*, *msrE* and *oxa23*) are nearly all absent from IC2 until around 2005, after which the proportion of isolates containing these genes increases rapidly, with the genes remaining common in IC2 to this day (Fig. 4a). A different set of genes (*catB8*, *aac(6*′)*-Ib9*, *bla_ADC-30_*, *aadA*, *sul1* and *qacEΔ1*), some of which also show rapid acquisition after around 2005, drop considerably in proportion after about 2015 and do not recover (Fig. 4b). These patterns of ARG presence/absence show that the genomic content of IC2 has been changing dramatically over time. Further, the fact that the acquisition of some of these genes coincides with the sudden expansion of IC2 suggests that this subset of ARGs may have contributed significantly to the success of IC2.

**Fig. 5.**
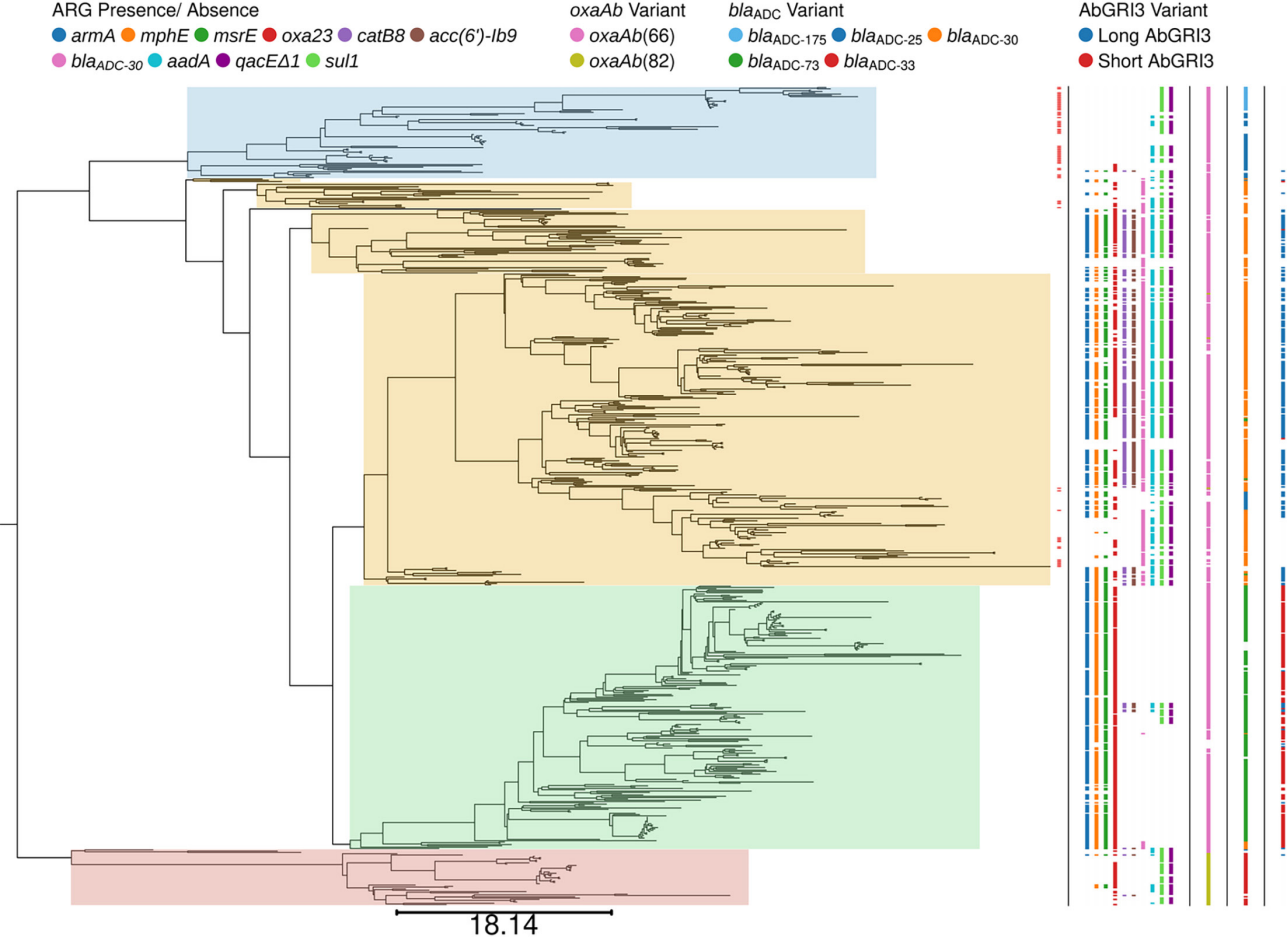
IC2 phylogeny highlighting groups. The phylogeny was created using Gubbins and outgroup rooted as described in the ‘Methods’ section. The scale bar indicates the total number of nucleotide substitutions as identified by Gubbins and annotated using TreeViewer. The four major groups are marked by background shading of the phylogeny: group 1 in blue, group 2 in yellow, group 3 in green and group 4 in red. The five datasets to the right of the phylogeny represent, from left to right, whether an isolate is historical, i.e. sequenced for this study (shown in red), the presence of ARGs, the *oxaAb* variant, the *bla*_ADC_ variant and the AbGRI3 variant. For simplicity, only ‘short’ and ‘long’ AbGRI3 variants were identified as stated in the Introduction. Only major *oxaAb* and *bla*_ADC_ variants are shown.

**Fig. 6.**
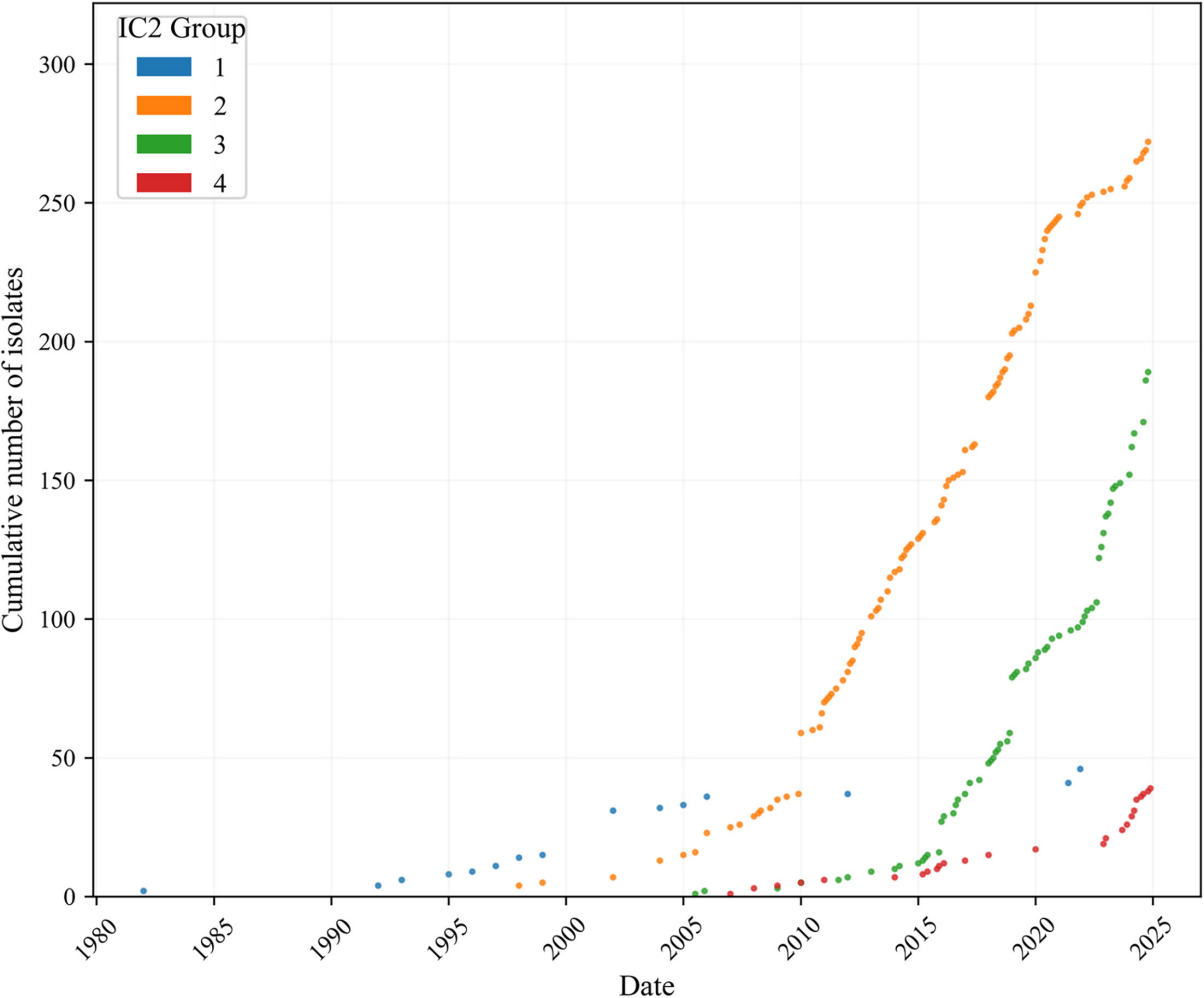
Cumulative number of IC2 group isolates over time. Each dot represents a single isolate and is coloured according to the groups shown in the legend.

To further investigate potential groups within IC2, an IC2 phylogeny was created from a recombination-free alignment and rooted with ATCC 19606 as an outgroup (see the ‘Methods’ section). Root-to-tip analysis gave an *R*^2^ value of 0.14, which was insufficient for dating. However, bootstrap support values were high across the phylogeny, especially in shallow branches, giving high confidence in major splits. Examining this phylogeny revealed a number of clades with shared ARG presence/absence patterns, splitting IC2 into at least four groups (Fig. 5). The occurrence of each group over time is shown in Fig. 6. Group 1 contains the oldest isolates and contains few IC2-specific ARGs aside from *aadA*, *sul1* and *qacEΔ1*, all of which are also enriched in IC1 (Fig. 3), suggesting these genes may be ancestral to both IC2 and IC1. Correspondingly, the genes *armA*, *mphE*, *msrE, catB8* and *aac(6*′)*-Ib9* were statistically significantly depleted in group 1. Group 2 contains the next most recent isolates, being the dominant IC2 group between 2005 and 2015 and accounting for a high proportion of IC2 isolates to this day. Most of its isolates contain the ARGs discussed previously (*armA*, *mphE*, *msrE* and *oxa23*), accounting for their sudden increase in frequency around 2005 (Fig. 4). These genes were not found to be statistically significantly enriched in this group. However, this is likely due to their high frequency in group 3. The genes *catB8*, *aac(6*′)*-Ib9, aadA*, *sul1,* and *qacEΔ1* were all statistically significantly enriched in this group due to being frequently found in these isolates. Group 3 represents the most recent clade of IC2 to emerge. While the first isolate was found in 2005, the group did not start rapidly expanding until ~2015. Of the ARGs shown in Fig. 4, only the subset *armA*, *mphE*, *msrE* and *oxa23* are widespread in these isolates, which accounts for the steep decline in frequency of other ARGs. Correspondingly, these genes were statistically significantly enriched in this group, while the genes *catB8*, *aac(6*′)*-Ib9, aadA*, *sul1* and *qacEΔ1* were statistically significantly depleted. The final major group, group 4, appears to have emerged independently of groups 2 and 3. While the first isolate was collected in 2007, the phylogeny suggests it is the oldest. There has been a notable increase in the isolation of *A. baumannii* belonging to group 4 since 2022. However, these recent isolates originated from the Utah Public Health Laboratory Infectious Disease submission group or the Centers for Disease Control and Prevention, both in the USA. Therefore, it is unclear whether this represents a recent expansion (as seen in groups 2 and 3 in 2005 and 2015, respectively) or is due to increased pathogen surveillance. Aside from the ARGs described above, group 4 isolates are deficient in almost all ARGs which are enriched in IC2 over other ICs, with *armA*, *mphE*, *msrE*, *catB8*, *aac(6*′)*-Ib9, bla_TEM-1_*, *tetR* and *tet(B*) all being statistically significantly depleted. This is with the notable exception of *oxa23*, *sul1* and *qacEΔ1*, which are common in isolates from this group, although only *sul1* and *qacEΔ1* were statistically significantly enriched, likely due to the high frequency of *oxa23* in groups 2 and 3.

### IC2 groups contain distinct variants of AbGRI3

Many of the ARGs (*aac(6*′)*-Ib9*, *catB8*, *aadA*, *qacEΔ1*, *sul1*, *armA*, *msrE* and *mphE*) that show group-specific presence/absence patterns are contained within AbGRI3. Due to the significant variance in the content and exact structure of AbGRI3 between individual isolates, we identified only ‘short’ and ‘long’ AbGRI3 variants as described in the ‘Introduction’ section. Groups 2 and 3 were enriched in long and short variants, respectively (Fig. 5). Since the AbGRI3-localized genes, which are absent from group 3 (*aac(6*′)*-Ib9*, *catB8*, *aadA*, *qacEΔ1* and *sul1*), are found in a single integron, the switch from long to short AbGRI3 likely occurred in a single deletion event.

**Fig. 7.**
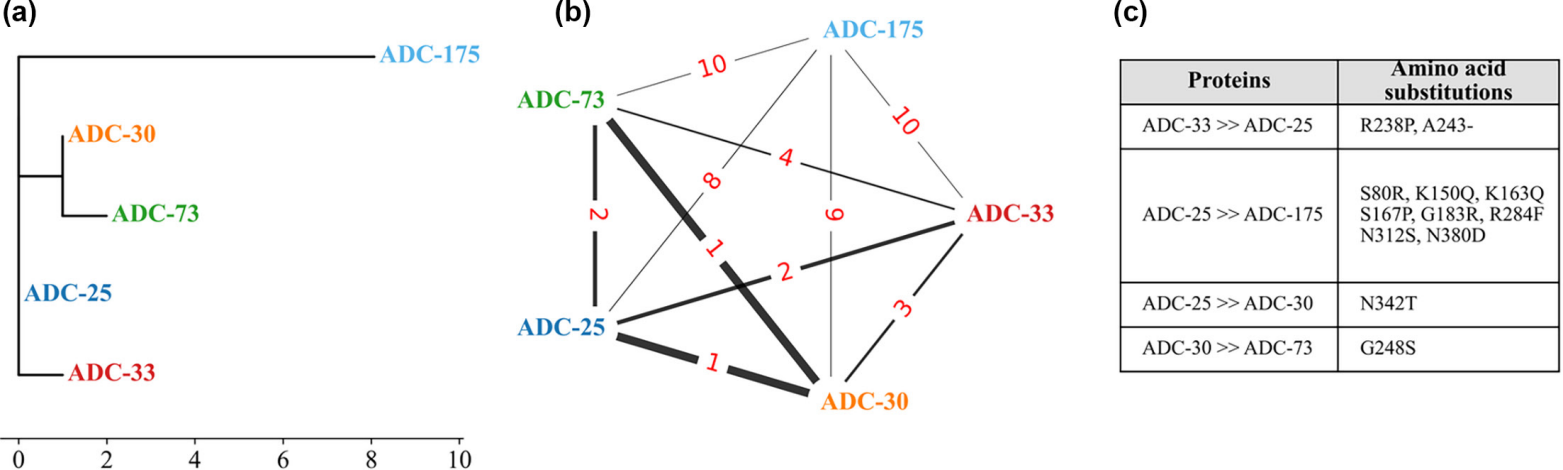
Amino acid differences between major ADC protein variants. (**a**) Phylogeny inferred from alignment of ADC amino acid sequences, with scale bar indicating number of amino acid substitutions. (**b**) Network diagram showing the total number of amino acid differences, shown in red, between ADC variants. Line thickness is inversely proportional to the number of mutations between each variant. (**c**) Table showing specific amino acid differences between selected ADC variants.

While ARG presence/absence patterns show a clear distinction between IC2 groups, which is congruent with the IC2 population structure represented by the recombination-free phylogeny, further analysis was performed to confirm how these groups are related by analysing the *oxaAb* and *bla*_ADC_ gene variants. The *bla*_ADC_ genes showed greater variation: group 1 contained mostly *bla_ADC-25_*, with a separate subgroup containing *bla*_ADC-175_; groups 2 and 3 contained *bla_ADC-30_* and *bla_ADC-73_*, respectively; and group 4 mainly contained *bla_ADC-33_* (Fig. 5). Analysis of amino acid differences between these genes suggests an evolution from *bla_ADC-25_* to *bla_ADC-30_* to *bla_ADC-73_* (Fig. 7a), with each gene differing from its predecessor by only a single amino acid substitution (Fig. 7b). *bla_ADC-33_* was most similar to *bla_ADC-25_* with only two amino acid substitutions (Fig. 7b). *bla_ADC-175_* was also most similar to *bla_ADC-25_*, but by eight substitutions which were distributed across the gene (Fig. 7c), suggesting significant evolutionary distance between these isolates and the rest of group 1, an inference supported by the protein phylogeny (Fig. 7a). Overall, this is consistent with the inference from the data on group prevalence (Fig. 6), AMR gene content and AbGRI3 structures (Fig. 5) that groups 1, 2 and 3 arose sequentially. Further, it supports the phylogeny in suggesting that group 4 is distinct from other groups.

The *oxaAb* genes showed less variation, with almost all group 1, 2 and 3 isolates having *oxaAb(66)*, and almost all group 4 isolates containing *oxaAb(82*) (Fig. 5). Although these two variants are separated by only a single SNP, this results in an L167V mutation that substantially increases carbapenemase activity through lowered Km [56]. However, since group 4 isolates often contain *oxa23*, it is unclear what further advantage this offers. Nonetheless, this confirms that group 4 is distinct from groups 1, 2, and 3.

### Group 4 isolates have few ARGs but many ISs

**Fig. 8.**
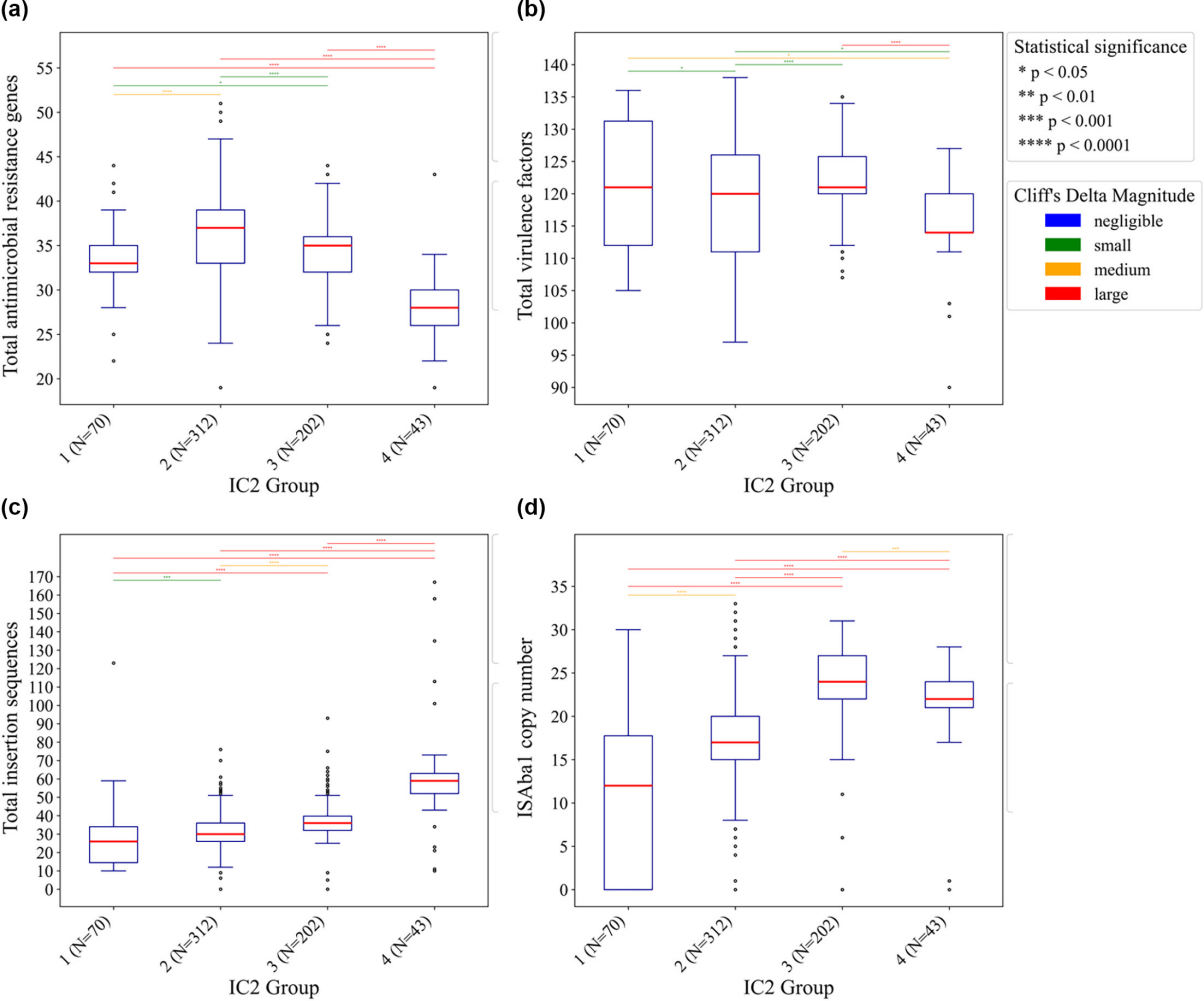
Boxplots of total ARGs, VFs, ISs and IS*Aba1* copies within chromosomes of IC2 group isolates. Total number of chromosomally encoded ARGs, VFs, ISs and ISAba1 are shown in (**a**), (**b**), (**c**) and (**d**), respectively. Medians are shown with red lines. Outliers (±1.5×IQR) are shown as open circles. Statistical significance (*P*<0.05) was determined with the Mann–Whitney U test with Holm–Bonferroni correction applied. Cliff’s delta was used to determine the effect size.

To further characterize the IC2 groups, ARG, VF and IS content across chromosomes of isolates from each group were analysed. In line with previous results, group 2 isolates had the most ARGs, followed by groups 3, 1 and 4 (Fig. 8a). Further analysis showed that, in addition to the ARGs shown in Fig. 4, group 4 isolates often lack other ARGs enriched in IC2, such as *bla_TEM-1_*, *tetR* and *tet(B*). Conversely, total VF gene content was similar between groups. Although there was a statistical difference in the distribution of total VFs within chromosomes, Cliff’s delta showed small effect sizes in most cases (Fig. 8b), and the high interquartile ranges (IQRs) make it difficult to draw comparisons. Therefore, although ARG content is distinct between different groups, the difference in VF gene content is negligible between groups.

Group 3 chromosomes were found to have the second-highest number of ISs, followed by groups 2 and 1 (Fig. 8c). However, group 4 chromosomes had the highest number of ISs (Fig. 8c). Further, while group 3 did have the highest median IS*Aba1* copy number, group 4 still had the second highest, followed by group 2 and finally group 1 (Fig. 8d). Further analysis showed that the high IS content in group 4 is driven mainly by IS*Aba13*, IS*Aba17* and IS*Aba125*. The latter of these was commonly associated with the ARG *aph(3′)-VIa* (also called *aphA6*), which in turn was mainly found in isolates dating from after 2022. IS*Aba125* contains an even stronger promoter than IS*Aba1* [57] and likely drives high expression of this ARG, potentially compensating for a lack of *armA* within group 4. Additionally, a median of 56.5% and 64% of IS*Aba13* and IS*Aba17*, respectively, was found within the K-locus or OC-locus. Therefore, we speculate that the group 4 isolates may have altered capsule and biofilm properties that alter their antimicrobial sensitivity phenotype, which may compensate for a relative lack of ARGs.

## Discussion

While IC2 is the most successful lineage of *A. baumannii*, the genomic factors behind its success and the evolution of its chromosome have remained poorly studied. We leveraged a set of historical *A. baumannii* isolates to identify a set of ARGs that we propose contributed to the success of *A. baumannii* IC2 over IC1. Further, we identify clades within IC2 which share common ARG presence/absence patterns, enabling us to split IC2 into four distinct subgroups. Each subgroup is associated with 1–2 *bla*_*ADC*_ genes, which may allow for future assignment of isolates to each of these groups.

Analyses of a number of aspects of IC2 suggest that three of the groups identified emerged in sequence over time, with corresponding gain or loss of a subset of ARGs. By collection date alone, the oldest IC2 isolates in our study are found in group 1 and, therefore, represent isolates that are closely related to the common ancestor with IC1. Consistent with this, group 1 isolates contain ARGs that are also enriched in IC1 (*aadA*, *sul1* and *qacEΔ1*). Group 2 seems to have expanded around 2005 with the acquisition of a large set of ARGs, many of which are associated with AbGRI3, which first appears in 2005 in this dataset. This is consistent with the proposition that AbGRI3 was originally acquired by IC2 sometime in the early 2000s [15]. Our data shows that the rapid expansion of group 2 around 2005 coincides with IC2 overtaking IC1 to become the dominant *A. baumannii* lineage, in line with other reports [18]. Therefore, it is probable that the acquisition of AbGRI3 and *oxa23* contributed to the rapid expansion and current success of IC2 over IC1. The rapidly expanding clade 2.5.6 reported by Li *et al*. [18] is equivalent to group 3, which our data also confirms began expanding in East Asia around 2005 and is expanding quickly in the USA and China. We found that group 3 began rapidly expanding around 2015 and has lost a subset of ARGs through genomic streamlining in AbGRI3. While some of these lost ARGs have been functionally superseded by other retained ARGs (e.g. *armA* provides broad aminoglycoside resistance), others provided resistance to antimicrobials which are not typically used against *A. baumannii* (*catB8* and *sul1*). Therefore, we propose that the loss of these genes provides a net fitness benefit to group 3 isolates. The proposed transition from group 1 to group 2, then group 3, is also reflected in increasing IS*Aba1* copy number. This aligns with the proposal that the acquisition of IS*Aba1*, and the genomic plasticity that this then engenders, was a major step in the evolution of *A. baumannii* as a pathogen [58,59]. Overall, groups 1, 2 and 3 reflect incremental adaptation of the *A. baumannii* IC2 chromosome to AMR selection over time.

In our study, we have identified group 4 as being quite distinct from the other three IC2 groups detected. Group 4 is not described by Li *et al*. [18] as a separate clade, likely due to the majority of these genomes not being present in their data. While the first isolate from group 4 was collected in 2007, *bla_ADC_* and *oxaAb* results suggest it may have split from other IC2 isolates earlier than this, with the phylogeny suggesting that it is the oldest group and most closely related to the common ancestor with IC1. Further, the subset of ARGs commonly found in this group is *aadA*, *sul1* and *qacEΔ1*, which suggests these isolates are derived from an early form of IC2 as discussed above. Notably, many isolates from this group contain *oxa23*. Despite lacking many ARGs which are enriched specifically in IC2, the chromosomes of group 4 isolates contain numerous copies of IS*Aba1*, IS*Aba125*, IS*Aba13* and IS*Aba17*. Colocalization between *oxa23*/IS*Aba1* and *aph(3′)-Via*/IS*Aba125* has been described in the literature, conferring resistance to carbapenems and amikacin, respectively [60,61]. Amikacin in particular is an aminoglycoside often used to treat infections by AMR Gram-negative bacteria, including *A. baumannii* [62,63]. Additional copies of IS*Aba13* and IS*Aba17* inserted into the K-locus and OC-locus may provide further resistance through disruptions of genes involved in capsule and biofilm biosynthesis. IS-mediated disruption of genes involved in these pathways, especially by IS*Aba13*, has been previously described, in some cases with corresponding increases in virulence and/or antimicrobial resistance [64,66]. Alterations in capsule and biofilm formation may reduce the need for ARGs against specific antimicrobials, compensating for the general lack of ARGs in these isolates, and are an area that merits further study. These mechanisms, some of which are rarely seen in IC2 isolates outside of group 4, may have enabled group 4 to persist at low levels, while its closest counterpart, group 1, became comparatively rare. There has been a notable increase in the isolation and sequencing of group 4 isolates since 2023, mainly driven by pathogen surveillance programmes in the USA. While it is hard to determine if this is due to increased sampling, we note similarities to the pattern of expansion in groups 2 and 3, and the periodicity at which new forms of IC2 emerge. Therefore, group 4 may be in the early stages of expansion.

We note that historical isolates sequenced and assembled here show a bias towards the UK and Europe. This resulted in a notable lack of isolates from Asia and the Middle East, despite these regions having *A. baumannii* outbreaks in the early 2000s [67]. This bias may have offset the detected date when a gene was first acquired by a few years if the gene was first acquired in these regions. For instance, the first reported occurrence of *armA* in *A. baumannii* was in Korea in 2003 [68], while the first occurrence in our dataset was in China in 2005. Nonetheless, these historical chromosomes provide significant insight into the evolution of the *A. baumannii* IC2 chromosome during its expansion, which would be extremely challenging with pre-existing data. Further, only publicly available genomes with complete or chromosome-level genomes were utilized. While this may have impacted the geographic and temporal distribution of isolates, an accurate whole-genome alignment could not be generated with chromosomes fragmented into multiple contigs, and recombination could not be removed with Gubbins without this alignment. The use of fragmented chromosomes would also have introduced uncertainty into ARG/VF detection. Therefore, draft genomes were excluded from this study. Plasmid sequences in publicly available genomes were analysed for ARG/VF content. Almost all plasmids contained no VFs and either one or no ARGs (Table S2) in line with previous studies [6,8]. Hence, plasmids were excluded from this study.

In conclusion, we leveraged a set of historical *A. baumannii* isolates to identify patterns of ARG gain and loss, which may have contributed to the success of IC2 over other *A. baumannii* lineages. Further, we find that IC2 can be split into at least four distinct groups, with matching ARG presence/absence patterns. While group 1 represents an early form of IC2, we propose that group 2 became highly successful in part due to the acquisition of *oxa23* and the formation of AbGRI3. Group 3 contains a streamlined form of AbGRI3, which we propose has enabled it to rapidly expand within the last decade. Group 4, meanwhile, is distinct from other groups with a parallel evolutionary history that may be in the early stages of expansion.

## Supplementary material

10.1099/mgen.0.001752 Supplementary Material 1.

## Abbreviations

ADC: *Acinetobacter*-derived cephalosporinase
AMR: antimicrobial resistance
ANI: average nucleotide identity
ARG: antimicrobial resistance gene
BUSCO: benchmarking universal single-copy orthologues
CARD: Comprehensive Antibiotic Resistance Database
IC1: international clone 1
IC2: international clone 2
IQRs: interquartile ranges
IS: insertion sequence
MLST: multi-locus sequence typing
ONT: Oxford Nanopore Technologies
RGI: resistance gene identifier
RIs: resistance islands
ST: sequence type
VFs: virulence factors
