# Supplementary material for: New isolates from the 1970s to early 2000s provide insights into the evolution of Acinetobacter baumannii international clone 2 and its resistome
Source: Microb Genom. 2026 Jul 2;12(7):001752. doi: 10.1099/mgen.0.001752 (PMC13324835; doi:10.1099/mgen.0.001752)
Supplement: Supplementary Material 1. [file mgen-12-01752-s001.pdf]

Supplemental data

Table S1 Historical *A. baumannii* chromosome metadata and quality control results for isolates sequenced in this study. Isolate number and BioSample accessions are shown. Multi-locus sequence typing (MLST) was performed according to the Pasteur scheme, and assignment to international clones (ICs) was conducted using existing data as stated in Methods. Benchmarking universal single copy orthologues (BUSCO) score, defined as percentage of complete and single-copy BUSCOs, and average nucleotide identity (ANI) to an ATCC 19606 reference genome is also shown for the chromosome of each isolate.

Genome ID	Biosample accession	Isolate number	Location	Parsed location	Collection date	Parsed collection date	Isolation source	Parsed isolation source	Sequence type	International clone	IC2 Group	BUSCO score	ANI to reference genome	ADC gene(s)
Chr_752_2	SAMN54724540	752	Saudi Arabia	Saudi Arabia	2006-2007	2006	Clinical_sample	Clinical_sample	new_ST	NA	NA	99.7	97.7	ADC-241
Chr_241_2	SAMN54724391	241	Missing	Missing	Missing	Missing	Missing	Missing	388	Not IC	NA	99.4	97.6	ADC-308
Chr_259_1	SAMN54724405	259	Missing	Missing	Missing	Missing	Missing	Missing	221	Not IC	NA	99.7	97.7	ADC-248
Chr_615_1	SAMN54724489	615	Missing	Missing	1997	1997	Missing	Missing	762	Not IC	NA	99.8	97.7	ADC-158
Chr_614_1	SAMN54724488	614	Missing	Missing	1997	1997	Missing	Missing	762	Not IC	NA	99.8	97.7	ADC-158
Chr_613_1	SAMN54724487	613	Missing	Missing	1997	1997	Missing	Missing	762	Not IC	NA	99.8	97.7	ADC-158
Chr_53_1	SAMN54724356	53	Freiburg, Germany	Germany	Missing	Missing	Hospital_enviro nment	Hospital_envirom ent	106	Not IC	NA	99.5	97.6	ADC-164
Chr_139_1	SAMN54724370	139	Ghent Belgium	Belgium	Missing	Missing	Missing	Missing	333	Not IC	NA	99.7	97.7	ADC-179
Chr_137_1	SAMN54724368	137	Ghent Belgium	Belgium	Missing	Missing	Missing	Missing	333	Not IC	NA	99.7	97.6	ADC-179
Chr_136_1	SAMN54724367	136	Ghent Belgium	Belgium	Missing	Missing	Missing	Missing	333	Not IC	NA	99.5	97.7	ADC-179
Chr_138_4	SAMN54724369	138	Ghent Belgium	Belgium	Missing	Missing	Missing	Missing	333	Not IC	NA	99.7	97.7	ADC-179
Chr_595_2	SAMN54724484	595	Missing	Missing	1983	1983	Missing	Missing	1140	Not IC	NA	99.4	97.5	ADC-159
Chr_769_3	SAMN54724553	769	Saudi Arabia	Saudi Arabia	2006-2007	2006	Clinical_sample	Clinical_sample	new_ST	NA	NA	99.5	97.8	ADC-203
Chr_17_1	SAMN54724342	17	Leeds, UK	United Kingdom	1993	1993	Clinical_sample	Clinical_sample	25	IC7	NA	99.7	97.7	ADC-342

Chr_666_1	SAMN54724506	666	South Africa	South Africa	2000	2000	Missing	Missing	25	IC7	NA	99.5	97.6	ADC-342
Chr_195_1	SAMN54724380	195	Chris Hani Baragwanath Hospital Soweto South Africa	South Africa	1996	1996	Pus	Skin	25	IC7	NA	99.6	97.6	ADC-342
Chr_492_1	SAMN54724478	492	Iraq isolates from US military, Iraq	Iraq	Missing	Missing	Missing	Missing	25	IC7	NA	99.3	97.5	ADC-342
Chr_753_1	SAMN54724541	753	Saudi Arabia	Saudi Arabia	2006-2007	2006	Clinical_sample	Clinical_sample	113	IC7	NA	99.7	97.6	ADC-57
Chr_746_1	SAMN54724535	746	Saudi Arabia	Saudi Arabia	2006-2007	2006	Clinical_sample	Clinical_sample	113	IC7	NA	99.7	97.6	ADC-57
Chr_747_1	SAMN54724536	747	Saudi Arabia	Saudi Arabia	2006-2007	2006	Clinical_sample	Clinical_sample	113	IC7	NA	99.6	97.6	ADC-57
Chr_744_1	SAMN54724533	744	Saudi Arabia	Saudi Arabia	2006-2007	2006	Clinical_sample	Clinical_sample	113	IC7	NA	99.8	97.6	ADC-57
Chr_743_1	SAMN54724532	743	Saudi Arabia	Saudi Arabia	2006-2007	2006	Clinical_sample	Clinical_sample	113	IC7	NA	99.7	97.5	ADC-57
Chr_755_1	SAMN54724543	755	Saudi Arabia	Saudi Arabia	2006-2007	2006	Clinical_sample	Clinical_sample	113	IC7	NA	99.7	97.5	ADC-57
Chr_478A_1	SAMN54724562	478A	EU Clone III	Missing	Missing	Missing	Missing	Missing	126	Not IC	NA	99.8	97.6	ADC-271
Chr_470_1	SAMN54724467	470	Warsaw Poland	Poland	2001-2004	2002	Hospital_environment	Hospital_enviroment	5	Not IC	NA	99.7	97.9	NA
Chr_473_1	SAMN54724469	473	Warsaw Poland	Poland	2003-2005	2004	Missing	Missing	5	Not IC	NA	99.5	97.8	NA
Chr_A46_1	SAMN54724565	A46	Hull UK	United Kingdom	1980-1982	1981	Missing	Missing	52	Not IC	NA	99.5	100	ADC-158
Chr_305_1	SAMN54724413	305	Newcasttle EU Clone II aac C2, UK	United Kingdom	1989	1989	Missing	Missing	438	Not IC	NA	99.6	99.7	ADC-158
Chr_593_1	SAMN54724483	593	Glasgow Royal	United Kingdom	1979	1979	Hospital_environment	Hospital_enviroment	438	Not IC	NA	99.7	99.6	ADC-158

			Infirmary UK											
Chr_807_1	SAMN54724559	807	Glasgow Royal Infirmary UK	United Kingdom	1979	1979	Hospital_environment	Hospital_enviroment	438	Not IC	NA	99.6	99.6	ADC-158
Chr_233_1	SAMN54724385	233	Missing	Missing	Missing	Missing	Missing	Missing	154	Not IC	NA	97.6	97.8	ADC-160
Chr_18_1	SAMN54724343	18	Leeds, UK	United Kingdom	1993	1993	Clinical_sample	Clinical_sample	154	Not IC	NA	99.5	97.8	ADC-160
Chr_27_1	SAMN54724346	27	Cardiff University of Wales Hospital, UK	United Kingdom	1993	1993	ICU	Hospital_enviroment	154	Not IC	NA	99.5	97.8	ADC-160
Chr_95_1	SAMN54724364	95	Trieste Italy	Italy	Missing	Missing	Missing	Missing	new_ST	NA	NA	99.8	97.8	ADC-25
Chr_761_1	SAMN54724548	761	Saudi Arabia	Saudi Arabia	2006-2007	2006	Clinical_sample	Clinical_sample	78	IC6	NA	99.6	97.9	ADC-52
Chr_758_2	SAMN54724545	758	Saudi Arabia	Saudi Arabia	2006-2007	2006	Clinical_sample	Clinical_sample	78	IC6	NA	99.7	98	ADC-52
Chr_740_1	SAMN54724529	740	Scotland Stirling or St. Andrews abattoir, UK	United Kingdom	2006-2007	2006	Pig	Pig	new_ST	NA	NA	99.8	97.9	ADC-237
Chr_616_2	SAMN54724490	616	Missing	Missing	1997	1997	Missing	Missing	1526	Not IC	NA	99.7	97.7	ADC-249
Chr_739_1	SAMN54724528	739	Scotland Stirling or St. Andrews abattoir, UK	United Kingdom	2006-2007	2006	Pig	Pig	new_ST	NA	NA	99.8	97.8	ADC-315
Chr_792_2	SAMN54724558	792	Hull Royal Infirmary UK	United Kingdom	1981	1981	Hospital_environment	Hospital_enviroment	29	Not IC	NA	99.6	97.8	ADC-2
Chr_576_5	SAMN54724480	576	Hull Royal Infirmary UK	United Kingdom	1981	1981	Hospital_environment	Hospital_enviroment	29	Not IC	NA	99.5	97.8	ADC-2

Chr_587_1	SAMN54724482	587	Hull Royal Infirmary UK	United Kingdom	1980	1980	Hospital_environment	Hospital_enviroment	29	Not IC	NA	99.5	97.9	ADC-2
Chr_31_1	SAMN54724348	31	Hull Royal Infirmary UK	United Kingdom	1981	1981	Hospital_environment	Hospital_enviroment	29	Not IC	NA	99.6	97.9	ADC-2
Chr_21_1	SAMN54724345	21	CHU Bichat Paris, France	France	1993	1993	Clinical_sample	Clinical_sample	32	Not IC	NA	99.7	97.7	ADC-79
Chr_764_1	SAMN54724550	764	Saudi Arabia	Saudi Arabia	2006-2007	2006	Clinical_sample	Clinical_sample	32	Not IC	NA	99.7	97.7	ADC-95
Chr_768_1	SAMN54724552	768	Saudi Arabia	Saudi Arabia	2006-2007	2006	Clinical_sample	Clinical_sample	32	Not IC	NA	99.6	97.7	ADC-95
Chr_759_1	SAMN54724546	759	Saudi Arabia	Saudi Arabia	2006-2007	2006	Clinical_sample	Clinical_sample	32	Not IC	NA	99.6	97.6	ADC-95
Chr_762_1	SAMN54724549	762	Saudi Arabia	Saudi Arabia	2006-2007	2006	Clinical_sample	Clinical_sample	53	Not IC	NA	99.1	97.7	ADC-79
Chr_269_1	SAMN54724407	269	Missing	Missing	1994	1994	Missing	Missing	267	Not IC	NA	99.4	97.8	ADC-291
Chr_198_1	SAMN54724382	198	Chris Hani Baragwanath Hospital Soweto South Africa	South Africa	1996	1996	Blood	Blood	new_ST	NA	NA	99.8	97.8	ADC-117
Chr_197_1	SAMN54724381	197	Chris Hani Baragwanath Hospital Soweto South Africa	South Africa	1996	1996	Pus	Skin	new_ST	NA	NA	99.8	97.8	ADC-117
Chr_189_1	SAMN54724379	189	Chris Hani Baragwanath Hospital Soweto South Africa	South Africa	1996	1996	Blood	Blood	625	Not IC	NA	99.6	97.9	ADC-158

Chr_741_1	SAMN54724530	741	Scotland Stirling or St. Andrews abattoir, UK	United Kingdom	2006-2007	2006	Pig	Pig	1198	Not IC	NA	99.8	97.8	ADC-270
Chr_169_1	SAMN54724376	169	Rohodes Susan	Greece	Missing	Missing	ICU	Hospital_enviroment	new_ST	NA	NA	99.7	97.7	ADC-328
Chr_238_1	SAMN54724388	238	Missing	Missing	Missing	Missing	Missing	Missing	2	IC2	1	99.6	98	ADC-175
Chr_442_2	SAMN54724455	442	Sofia Bulgaria	Bulgaria	2001-2004	2002	Hospital_environment	Hospital_enviroment	2	IC2	1	99.6	97.9	ADC-175
Chr_237_2	SAMN54724387	237	Missing	Missing	Missing	Missing	Missing	Missing	2	IC2	1	99.6	97.9	ADC-175
Chr_239_1	SAMN54724389	239	Missing	Missing	Missing	Missing	Missing	Missing	2	IC2	1	99.6	98	ADC-175
Chr_394_2	SAMN54724430	394	Thessalonica Greece	Greece	2001-2004	2002	Hospital_environment	Hospital_enviroment	2	IC2	1	99.5	97.9	ADC-175
Chr_397_1	SAMN54724431	397	Thessalonica Greece	Greece	2001-2004	2002	Clinical_sample	Clinical_sample	2	IC2	1	99.3	97.9	ADC-175
Chr_373_1	SAMN54724420	373	Athens Greece	Greece	2004	2004	Hospital_environment	Hospital_enviroment	2	IC2	1	99.3	98	ADC-175
Chr_385_2	SAMN54724424	385	Ioannina Greece	Greece	2001-2004	2002	Hospital_environment	Hospital_enviroment	2	IC2	1	99.6	98	ADC-175
Chr_490_5	SAMN54724477	490	Missing	Missing	Missing	Missing	ICU	Hospital_enviroment	2	IC2	1	99.3	97.9	ADC-175
Chr_489_2	SAMN54724476	489	Imported from Crete, Greece	Greece	Missing	Missing	Missing	Missing	2	IC2	1	99.3	97.9	ADC-175
Chr_279_1	SAMN54724410	279	Innsbruck, Austria	Austria	Missing	Missing	Missing	Missing	2	IC2	1	99.5	98	ADC-175
Chr_280_2	SAMN54724411	280	Innsbruck	Austria	Missing	Missing	Missing	Missing	2	IC2	1	99.5	97.9	ADC-175
Chr_240_1	SAMN54724390	240	Missing	Missing	Missing	Missing	Missing	Missing	2	IC2	1	99.5	97.9	ADC-175
Chr_236_5	SAMN54724386	236	Missing	Missing	Missing	Missing	Missing	Missing	2	IC2	1	99.6	97.9	ADC-175
Chr_242_3	SAMN54724392	242	Missing	Missing	Missing	Missing	Missing	Missing	2	IC2	1	99.6	97.9	ADC-175
Chr_140_2	SAMN54724371	140	Cologne Germany	Germany	Missing	Missing	Missing	Missing	2	IC2	1	99.5	97.9	ADC-175
Chr_387_1	SAMN54724425	387	Ioannina Greece	Greece	2001-2004	2002	Clinical_sample	Clinical_sample	45	IC2	1	99.7	97.8	ADC-185

Chr_231_1	SAMN54724384	231	Missing	Missing	1995	1995	ICU	Hospital_enviroment	2	IC2	1	99.6	97.9	ADC-25
Chr_65_2	SAMN54724361	65	Nottingham	United Kingdom	1992	1992	Hospital_environment	Hospital_enviroment	2	IC2	1	99.5	98	ADC-25
Chr_66_1	SAMN54724362	66	Nottingham	United Kingdom	1992	1992	Hospital_environment	Hospital_enviroment	2	IC2	1	99.6	97.9	ADC-25
Chr_89_1	SAMN54724363	89	Nottingham, UK	United Kingdom	1993	1993	Hospital_environment	Hospital_enviroment	2	IC2	1	99.7	98	ADC-25
Chr_320_1	SAMN54724415	320	Rottendam EU Clone II	Netherlands	1982	1982	Missing	Missing	2	IC2	1	99.7	98	ADC-25
Chr_48_1	SAMN54724352	48	Berlin, Germany	Germany	Missing	Missing	Missing	Missing	2	IC2	1	99.6	98	ADC-25
Chr_47_1	SAMN54724351	47	Berlin, Germany	Germany	Missing	Missing	Missing	Missing	2	IC2	1	99.7	98	ADC-25
Chr_40_1	SAMN54724349	40	Singapore	Singapore	Missing	Missing	Missing	Missing	2	IC2	1	99.6	98	ADC-25
Chr_50_1	SAMN54724353	50	Berlin, Germany	Germany	Missing	Missing	Missing	Missing	2	IC2	1	99.6	98	ADC-25
Chr_650_1	SAMN54724501	650	Turkey	Türkiye	1998	1998	Missing	Missing	2	IC2	1	99.6	98	ADC-25
Chr_28_1	SAMN54724347	28	East Glamorgan Hospital, UK	United Kingdom	1993	1993	ICU	Hospital_enviroment	2	IC2	1	99.5	98	ADC-25
Chr_410_1	SAMN54724434	410	Dolmoslaskie Centrum Wroclaw Poland	Poland	2001-2004	2002	Clinical_sample	Clinical_sample	2	IC2	1	99.3	97.9	ADC-25 # ADC-25
Chr_417_1	SAMN54724439	417	Medical University Wroclaw Poland	Poland	2001-2004	2002	Hospital_environment	Hospital_enviroment	2	IC2	1	99.4	97.8	ADC-25
Chr_432_1	SAMN54724451	432	Croatia	Croatia	2001-2004	2002	Hospital_environment	Hospital_enviroment	2	IC2	1	99.5	97.7	ADC-25
Chr_427_1	SAMN54724448	427	Croatia	Croatia	2001-2004	2002	Hospital_environment	Hospital_enviroment	2	IC2	1	99.4	97.7	ADC-25
Chr_418_1	SAMN54724440	418	Medical University	Poland	2001-2004	2002	Hospital_environment	Hospital_enviroment	2	IC2	1	99.6	97.8	ADC-25

			Wroclaw Poland											
Chr_419_1	SAMN54724441	419	Medical University Wroclaw Poland	Poland	2001-2004	2002	Hospital_environment	Hospital_enviroment	2	IC2	1	99.6	97.9	ADC-25
Chr_421_1	SAMN54724443	421	Medical University Wroclaw Poland	Poland	2001-2004	2002	Hospital_environment	Hospital_enviroment	2	IC2	1	99.3	97.7	ADC-25
Chr_420_1	SAMN54724442	420	Medical University Wroclaw Poland	Poland	2001-2004	2002	Hospital_environment	Hospital_enviroment	2	IC2	1	99.2	97.9	ADC-25
Chr_392_1	SAMN54724428	392	Freiburg Germany	Germany	2001-2004	2002	Clinical_sample	Clinical_sample	2	IC2	1	99.6	97.9	ADC-25
Chr_756_1	SAMN54724544	756	Saudi Arabia	Saudi Arabia	2006-2007	2006	Clinical_sample	Clinical_sample	2	IC2	1	99.4	97.9	ADC-25
Chr_629_3	SAMN54724494	629	Turkey	Türkiye	1997	1997	Missing	Missing	2	IC2	1	99.3	98	ADC-25
Chr_368_4	SAMN54724417	368	Missing	Missing	Missing	Missing	Missing	Missing	2	IC2	1	99.7	98	ADC-1
Chr_217_2	SAMN54724383	217	Royal Hallamshire Sheffield, UK	United Kingdom	1996	1996	Missing	Missing	2	IC2	1	99.7	98	ADC-1
Chr_267_1	SAMN54724406	267	Missing	Missing	1995	1995	Missing	Missing	2	IC2	1	99.6	97.9	ADC-1
Chr_370_1	SAMN54724418	370	Oviedo Spain	Spain	2001-2004	2002	Hospital_environment	Hospital_enviroment	2	IC2	1	99.3	97.8	ADC-260
Chr_465_1	SAMN54724466	465	Portugal Carnaxide	Portugal	2001-2004	2002	Hospital_environment	Hospital_enviroment	2	IC2	1	99.5	97.9	ADC-1
Chr_774_1	SAMN54724555	774	Saudi Arabia	Saudi Arabia	2006-2007	2006	Clinical_sample	Clinical_sample	2	IC2	1	99.5	97.9	ADC-25
Chr_771_2	SAMN54724554	771	Saudi Arabia	Saudi Arabia	2006-2007	2006	Clinical_sample	Clinical_sample	2	IC2	1	99.7	97.9	ADC-25
Chr_641_1	SAMN54724495	641	Singapore	Singapore	1998	1998	Missing	Missing	2	IC2	1	99.5	98	ADC-25
Chr_647_1	SAMN54724500	647	Singapore	Singapore	1998	1998	Missing	Missing	2	IC2	1	99.5	97.9	ADC-25
Chr_745_1	SAMN54724534	745	Saudi Arabia	Saudi Arabia	2006-2007	2006	Clinical_sample	Clinical_sample	2	IC2	2	99.5	97.8	ADC-30

Chr_749_1	SAMN54724538	749	Saudi Arabia	Saudi Arabia	2006-2007	2006	Clinical_sample	Clinical_sample	2	IC2	2	99.6	97.9	ADC-30
Chr_481B_4	SAMN54724564	481B	Missing	Missing	Missing	Missing	Missing	Missing	2	IC2	2	98.6	97.8	ADC-30
Chr_484_1	SAMN54724473	484	Nottingham UK	United Kingdom	Missing	Missing	Missing	Missing	2	IC2	2	99.1	97.9	ADC-30
Chr_480_1	SAMN54724470	480	Missing	Missing	Missing	Missing	Missing	Missing	2	IC2	2	99.3	97.9	ADC-30
Chr_485_1	SAMN54724474	485	Missing	Missing	Missing	Missing	Missing	Missing	2	IC2	2	99.3	98	ADC-30
Chr_482_2	SAMN54724471	482	Missing	Missing	Missing	Missing	Missing	Missing	2	IC2	2	99	97.9	ADC-30
Chr_383_2	SAMN54724423	383	Chelsea Westminster, UK	United Kingdom	2001-2004	2002	Hospital_environment	Hospital_enviroment	2	IC2	2	99.2	97.9	ADC-143
Chr_659_4	SAMN54724503	659	Singapore	Singapore	1998	1998	Missing	Missing	2	IC2	2	99.6	97.9	ADC-30
Chr_644_1	SAMN54724498	644	Singapore	Singapore	1998	1998	Missing	Missing	2	IC2	2	99.6	97.8	ADC-30
Chr_645_1	SAMN54724499	645	Singapore	Singapore	1998	1998	Missing	Missing	2	IC2	2	99.6	97.8	ADC-30
Chr_43_1	SAMN54724350	43	Singapore	Singapore	Missing	Missing	Missing	Missing	2	IC2	2	99.7	97.9	ADC-30
Chr_643_1	SAMN54724497	643	Singapore	Singapore	1998	1998	Missing	Missing	2	IC2	2	99.6	97.9	ADC-30
Chr_454_1	SAMN54724463	454	Nitra Slovakia	Slovakia	2001-2004	2002	Hospital_environment	Hospital_enviroment	2	IC2	2	99.6	97.9	ADC-30
Chr_669_2	SAMN54724507	669	USA	United States	2004	2004	Missing	Missing	2	IC2	2	99.6	97.9	ADC-30
Chr_380_1	SAMN54724422	380	Chelsea Westminster, UK	United Kingdom	2001-2004	2002	Clinical_sample	Clinical_sample	new_alleles	NA	NA	99.3	97.7	ADC-30
Chr_748_1	SAMN54724537	748	Saudi Arabia	Saudi Arabia	2006-2007	2006	Clinical_sample	Clinical_sample	2	IC2	2	99.5	97.9	ADC-30
Chr_760_1	SAMN54724547	760	Saudi Arabia	Saudi Arabia	2006-2007	2006	Clinical_sample	Clinical_sample	2	IC2	2	99.5	97.9	ADC-115
Chr_776_1	SAMN54724557	776	Saudi Arabia	Saudi Arabia	2006-2007	2006	Clinical_sample	Clinical_sample	2	IC2	2	99.5	97.9	ADC-115
Chr_498_1	SAMN54724479	498	Missing	Missing	Missing	Missing	Missing	Missing	2	IC2	2	99.5	97.9	ADC-30
Chr_733_1	SAMN54724526	733	Scotland, UK	United Kingdom	2006-2007	2006	Missing	Missing	492	IC2	NA	99.8	97.8	ADC-169
Chr_737_1	SAMN54724527	737	Scotland Stirling or St. Andrews	United Kingdom	2006-2007	2006	Sheep	Sheep	1061	Not IC	NA	99.8	97.8	ADC-303

			abattoir, UK											
Chr_765_3	SAMN54724551	765	Saudi Arabia	Saudi Arabia	2006-2007	2006	Clinical_sample	Clinical_sample	2167	Not IC	NA	99.7	97.7	ADC-2
Chr_750_1	SAMN54724539	750	Saudi Arabia	Saudi Arabia	2006-2007	2006	Clinical_sample	Clinical_sample	2167	Not IC	NA	99.7	97.7	ADC-2
Chr_754_2	SAMN54724542	754	Saudi Arabia	Saudi Arabia	2006-2007	2006	Clinical_sample	Clinical_sample	2167	Not IC	NA	99.7	97.7	ADC-2
Chr_272_1	SAMN54724408	272	Missing	Missing	1996	1996	Missing	Missing	2371	Not IC	NA	99.7	97.7	ADC-97
Chr_287_2	SAMN54724412	287	Christchurch N.Z.	New Zealand	2001-2004	2002	Clinical_sample	Clinical_sample	79	IC5	NA	99.5	97.7	ADC-5
Chr_62_1	SAMN54724359	62	Argentina	Argentina	Missing	Missing	Missing	Missing	79	IC5	NA	99.7	97.7	ADC-5
Chr_663_2	SAMN54724504	663	Buenos Aires Argentina	Argentina	1998	1998	Missing	Missing	79	IC5	NA	99.7	97.6	ADC-263
Chr_664_2	SAMN54724505	664	Buenos Aires Argentina	Argentina	1998	1998	Missing	Missing	79	IC5	NA	99.7	97.7	ADC-263
Chr_255_1	SAMN54724403	255	Missing	Missing	Missing	Missing	Missing	Missing	10	IC8	NA	99.6	97.8	ADC-76
Chr_243_1	SAMN54724393	243	Missing	Missing	Missing	Missing	Missing	Missing	10	IC8	NA	99.5	97.8	ADC-76
Chr_245_2	SAMN54724394	245	Missing	Missing	Missing	Missing	Missing	Missing	10	IC8	NA	99.3	97.8	ADC-76
Chr_246_1	SAMN54724395	246	Missing	Missing	Missing	Missing	Missing	Missing	10	IC8	NA	99.1	97.7	ADC-76
Chr_251_1	SAMN54724399	251	Missing	Missing	Missing	Missing	Missing	Missing	10	IC8	NA	99.7	97.7	ADC-76
Chr_254_1	SAMN54724402	254	Missing	Missing	Missing	Missing	Missing	Missing	10	IC8	NA	99.7	97.7	ADC-76
Chr_252_2	SAMN54724400	252	Missing	Missing	Missing	Missing	Missing	Missing	10	IC8	NA	99.7	97.7	ADC-76
Chr_249_1	SAMN54724397	249	Missing	Missing	Missing	Missing	Missing	Missing	10	IC8	NA	99.5	97.7	ADC-76
Chr_248_1	SAMN54724396	248	Missing	Missing	Missing	Missing	Missing	Missing	10	IC8	NA	99.7	97.8	ADC-76
Chr_253_1	SAMN54724401	253	Missing	Missing	Missing	Missing	Missing	Missing	10	IC8	NA	99.1	97.7	ADC-76
Chr_250_1	SAMN54724398	250	Missing	Missing	Missing	Missing	Missing	Missing	10	IC8	NA	99.6	97.7	ADC-76
Chr_257_2	SAMN54724404	257	Missing	Missing	Missing	Missing	Missing	Missing	10	IC8	NA	99.7	97.8	ADC-76
Chr_483_1	SAMN54724472	483	Agardir Imported from Morocco	Morocco	2001-2004	2002	Clinical_sample	Clinical_sample	10	IC8	NA	98.7	97.6	ADC-76

Chr_180_1	SAMN54724378	180	Chris Hani Baragwanath Hospital Soweto South Africa	South Africa	1996	1996	Hospital_environment	Hospital_enviroment	10	IC8	NA	99.7	97.6	ADC-76
Chr_56_2	SAMN54724358	56	Missing	Missing	1982	1982	Missing	Missing	10	IC8	NA	99.5	97.6	ADC-76
Chr_376_1	SAMN54724421	376	Feldkirchen Austria	Austria	2002	2002	Clinical_sample	Clinical_sample	23	IC8	NA	99.5	97.6	ADC-76
Chr_457_1	SAMN54724465	457	Tallinn Estonia	Estonia	2001-2004	2002	Clinical_sample	Clinical_sample	1035	Not IC	NA	99.7	97.7	ADC-280
Chr_742_1	SAMN54724531	742	Saudi Arabia	Saudi Arabia	2006-2007	2006	Clinical_sample	Clinical_sample	216	Not IC	NA	98.8	97.5	ADC-154
Chr_582_1	SAMN54724481	582	UK	United Kingdom	1983	1983	Missing	Missing	new_ST	NA	NA	99.5	97.6	ADC-99
Chr_692_1	SAMN54724523	692	Buenos Aires Argentina	Argentina	1994	1994	Hospital_environment	Hospital_enviroment	15	IC4	NA	99.5	97.8	ADC-2
Chr_694_2	SAMN54724525	694	Buenos Aires Argentina	Argentina	1994	1994	Hospital_environment	Hospital_enviroment	15	IC4	NA	99.5	97.7	ADC-2
Chr_693_1	SAMN54724524	693	Buenos Aires Argentina	Argentina	1994	1994	Hospital_environment	Hospital_enviroment	15	IC4	NA	99.5	97.7	ADC-2
Chr_690_2	SAMN54724522	690	Buenos Aires Argentina	Argentina	1993	1993	Hospital_environment	Hospital_enviroment	15	IC4	NA	99.5	97.8	ADC-2
Chr_393_14	SAMN54724429	393	Freiburg Germany	Germany	Missing	Missing	Missing	Missing	15	IC4	NA	99.6	97.8	ADC-77
Chr_687_2	SAMN54724520	687	Izmir Turkey	Türkiye	2005	2005	Missing	Missing	15	IC4	NA	99.4	97.8	ADC-77
Chr_682_1	SAMN54724516	682	Izmir Turkey	Türkiye	2005	2005	Missing	Missing	15	IC4	NA	99.5	97.9	ADC-77
Chr_681_2	SAMN54724515	681	Izmir Turkey	Türkiye	2005	2005	Missing	Missing	15	IC4	NA	99.6	97.8	ADC-77
Chr_680_1	SAMN54724514	680	Izmir Turkey	Türkiye	2005	2005	Missing	Missing	15	IC4	NA	99.5	97.8	ADC-77

Chr_688_1	SAMN54724521	688	Izmir Turkey	Türkiye	2005	2005	Missing	Missing	15	IC4	NA	99.5	97.8	ADC-77
Chr_684_2	SAMN54724518	684	Izmir Turkey	Türkiye	2005	2005	Missing	Missing	15	IC4	NA	99.5	97.8	ADC-77
Chr_686_3	SAMN54724519	686	Izmir Turkey	Türkiye	2005	2005	Missing	Missing	15	IC4	NA	99.5	97.9	ADC-77
Chr_683_2	SAMN54724517	683	Izmir Turkey	Türkiye	2005	2005	Missing	Missing	15	IC4	NA	99.6	97.9	ADC-77
Chr_679_1	SAMN54724513	679	Izmir Turkey	Türkiye	2005	2005	Missing	Missing	84	IC4	NA	99.1	97.8	ADC-77
Chr_677_5	SAMN54724511	677	Izmir Turkey	Türkiye	2005	2005	Missing	Missing	84	IC4	NA	99.3	97.7	ADC-77
Chr_678_2	SAMN54724512	678	Izmir Turkey	Türkiye	2005	2005	Missing	Missing	84	IC4	NA	99.4	97.7	ADC-77
Chr_144_1	SAMN54724372	144	Copenhagen Denmark	Denmark	Missing	Missing	Hospital_enviro nment	Hospital_envirom ent	15	IC4	NA	99.5	97.8	ADC-2
Chr_128_12	SAMN54724365	128	Belfast, Ireland	Ireland	Missing	Missing	Missing	Missing	15	IC4	NA	99.5	97.8	ADC-185
Chr_273_1	SAMN54724409	273	Johannesburg. Straim, South Africa	South Africa	Missing	Missing	Missing	Missing	3	IC3	NA	99.6	97.6	ADC-79
Chr_478B_1	SAMN54724563	478B	EU Clone III	Missing	Missing	Missing	Missing	Missing	3	IC3	NA	99.3	97.9	ADC-119
Chr_673_1	SAMN54724509	673	Spain	Spain	2005	2005	Missing	Missing	3	IC3	NA	99.5	97.7	ADC-119
Chr_332_1	SAMN54724416	332	Bilbao Spain	Spain	1998-1999	1998	Sputum	Respiratory_tract	3	IC3	NA	99.4	97.7	ADC-119
Chr_674_1	SAMN54724510	674	Spain	Spain	2005	2005	Missing	Missing	3	IC3	NA	99.5	97.7	ADC-1
Chr_609_1	SAMN54724485	609	Spain	Spain	1997	1997	Missing	Missing	new_alleles	NA	NA	99.5	97.8	ADC-119
Chr_610_1	SAMN54724486	610	Spain	Spain	1997	1997	Missing	Missing	new_alleles	NA	NA	99.6	97.8	ADC-119
Chr_372_1	SAMN54724419	372	Athens Greece	Greece	2003	2003	Clinical_sample	Clinical_sample	1	IC1	NA	99.5	97.6	ADC-81
Chr_433_1	SAMN54724452	433	Croatia	Croatia	2001-2004	2002	Hospital_enviro nment	Hospital_envirom ent	1	IC1	NA	99.6	97.6	ADC-11

Chr_446_1	SAMN54724458	446	Poland	Poland	2001-2004	2002	Hospital_environment	Hospital_enviroment	1	IC1	NA	99.5	97.6	ADC-25
Chr_448_1	SAMN54724460	448	Poland	Poland	2001-2004	2002	Hospital_environment	Hospital_enviroment	1	IC1	NA	99.6	97.6	ADC-25
Chr_447_1	SAMN54724459	447	Poland	Poland	2001-2004	2002	Hospital_environment	Hospital_enviroment	1	IC1	NA	99.7	97.6	ADC-25
Chr_449_1	SAMN54724461	449	Poland	Poland	2001-2004	2002	Hospital_environment	Hospital_enviroment	1	IC1	NA	99.6	97.6	ADC-25
Chr_444_2	SAMN54724456	444	Poland	Poland	2001-2004	2002	Hospital_environment	Hospital_enviroment	1	IC1	NA	99.7	97.6	ADC-25
Chr_451_1	SAMN54724462	451	Wroclaw Poland	Poland	2001-2004	2002	Hospital_environment	Hospital_enviroment	1	IC1	NA	99.6	97.6	ADC-25
Chr_445_2	SAMN54724457	445	Poland	Poland	2001-2004	2002	Hospital_environment	Hospital_enviroment	1	IC1	NA	99.7	97.6	ADC-25
Chr_472_1	SAMN54724468	472	Warsaw Poland	Poland	2003-2005	2004	Missing	Missing	1	IC1	NA	99.5	97.6	ADC-25
Chr_414_1	SAMN54724438	414	Medical University Wroclaw Poland	Poland	2001-2004	2002	Clinical_sample	Clinical_sample	1	IC1	NA	99.7	97.6	ADC-25
Chr_413_2	SAMN54724437	413	Dolmoslaskie Centrum Wroclaw Poland	Poland	2001-2004	2002	Hospital_environment	Hospital_enviroment	1	IC1	NA	99.4	97.6	ADC-178
Chr_411_1	SAMN54724435	411	Dolmoslaskie Centrum Wroclaw Poland	Poland	2001-2004	2002	Clinical_sample	Clinical_sample	1	IC1	NA	99.7	97.6	ADC-25
Chr_407_1	SAMN54724432	407	Dolmoslaskie Centrum Wroclaw Poland	Poland	2001-2004	2002	Hospital_environment	Hospital_enviroment	1	IC1	NA	99.6	97.6	ADC-178
Chr_409_1	SAMN54724433	409	Dolmoslaskie Centrum Wroclaw Poland	Poland	2001-2004	2002	Hospital_environment	Hospital_enviroment	1	IC1	NA	99.4	97.6	ADC-25

Chr_412_2	SAMN54724436	412	Dolmoslaskie Centrum Wroclaw Poland	Poland	2001-2004	2002	Hospital_environment	Hospital_enviroment	1	IC1	NA	99.7	97.6	ADC-25
Chr_167_2	SAMN54724375	167	Bosnia	Bosnia	Missing	Missing	Missing	Missing	1	IC1	NA	99.7	97.6	ADC-79
Chr_391_2	SAMN54724427	391	Pleven Bulgaria	Bulgaria	2001-2004	2002	Hospital_environment	Hospital_enviroment	1	IC1	NA	99.6	97.7	ADC-79
Chr_390_1	SAMN54724426	390	Pleven Bulgaria	Bulgaria	2001-2004	2002	Clinical_sample	Clinical_sample	1	IC1	NA	99.5	97.6	ADC-79
Chr_424_2	SAMN54724445	424	Zagreb Croatia	Croatia	2001-2004	2002	Clinical_sample	Clinical_sample	1	IC1	NA	99.6	97.6	ADC-176
Chr_428_1	SAMN54724449	428	Croatia	Croatia	2001-2004	2002	Hospital_environment	Hospital_enviroment	1	IC1	NA	99.5	97.6	ADC-176
Chr_426_1	SAMN54724447	426	Croatia	Croatia	2001-2004	2002	Hospital_environment	Hospital_enviroment	1	IC1	NA	99.6	97.7	ADC-176
Chr_431_1	SAMN54724450	431	Croatia	Croatia	2001-2004	2002	Hospital_environment	Hospital_enviroment	1	IC1	NA	99.5	97.7	ADC-176
Chr_425_4	SAMN54724446	425	Croatia	Croatia	2001-2004	2002	Hospital_environment	Hospital_enviroment	1	IC1	NA	99.5	97.6	ADC-176
Chr_422_1	SAMN54724444	422	Croatia	Croatia	2001-2004	2002	Hospital_environment	Hospital_enviroment	new_alleles	NA	NA	99.6	97.6	ADC-176
Chr_441_1	SAMN54724454	441	Sofia Bulgaria	Bulgaria	2001-2004	2002	Hospital_environment	Hospital_enviroment	1	IC1	NA	97.8	97.6	ADC-79
Chr_438_1	SAMN54724453	438	Sofia Bulgaria	Bulgaria	2001-2004	2002	Clinical_sample	Clinical_sample	1	IC1	NA	99.7	97.6	ADC-79
Chr_671_1	SAMN54724508	671	USA	United States	2004	2004	Missing	Missing	1	IC1	NA	99.4	97.6	NA
Chr_653_1	SAMN54724502	653	Singapore	Singapore	1998	1998	Missing	Missing	1	IC1	NA	99.6	97.7	ADC-191
Chr_627_2	SAMN54724493	627	Turkey	Türkiye	1997	1997	Missing	Missing	231	IC1	NA	99.7	97.7	ADC-11
Chr_622_1	SAMN54724491	622	Turkey	Türkiye	1997	1997	Missing	Missing	231	IC1	NA	99.5	97.7	ADC-11
Chr_625_3	SAMN54724492	625	Turkey	Türkiye	1997	1997	Missing	Missing	231	IC1	NA	99.7	97.7	ADC-11
Chr_775_2	SAMN54724556	775	Saudi Arabia	Saudi Arabia	2006-2007	2006	Clinical_sample	Clinical_sample	1	IC1	NA	99.8	97.8	ADC-11
Chr_456_1	SAMN54724464	456	Austria	Austria	2001-2004	2002	Hospital_environment	Hospital_enviroment	20	IC1	NA	99.6	97.7	ADC-74
Chr_487_2	SAMN54724475	487	France	France	Missing	Missing	Missing	Missing	1	IC1	NA	98.9	97.7	ADC-11

Chr_156_1	SAMN54724374	156	Venlo Netherlands	Netherlands	Missing	Missing	Missing	Missing	1	IC1	NA	99.5	97.6	ADC-11
Chr_154_1	SAMN54724373	154	Venlo Netherlands	Netherlands	Missing	Missing	Missing	Missing	1	IC1	NA	99.5	97.6	ADC-11
Chr_14_1	SAMN54724340	14	Venlo, Netherlands	Netherlands	1986	1986	Sputum	Respiratory_tract	1	IC1	NA	99.5	97.5	ADC-11
Chr_306_1	SAMN54724414	306	Missing	Missing	Missing	Missing	Missing	Missing	1	IC1	NA	99.5	97.6	ADC-11
Chr_51_2	SAMN54724354	51	Freiburg, Germany	Germany	Missing	Missing	Hospital_environment	Hospital_enviroment	1	IC1	NA	99.2	97.6	ADC-11
Chr_55_1	SAMN54724357	55	Freiburg, Germany	Germany	Missing	Missing	Hospital_environment	Hospital_enviroment	1	IC1	NA	99.6	97.7	ADC-11
Chr_52_1	SAMN54724355	52	Freiburg, Germany	Germany	Missing	Missing	Hospital_environment	Hospital_enviroment	1	IC1	NA	99.7	97.6	ADC-11
Chr_63_1	SAMN54724360	63	Argentina	Argentina	Missing	Missing	Missing	Missing	1	IC1	NA	99.5	97.7	ADC-11
Chr_134_1	SAMN54724366	134	Missing	Missing	1977	1977	Missing	Missing	1	IC1	NA	99.7	97.6	ADC-11
Chr_176_1	SAMN54724377	176	Nottingham UK	United Kingdom	1980	1980	Missing	Missing	1	IC1	NA	99.7	97.6	ADC-11
Chr_832_1	SAMN54724561	832	St. Thomas' Hospital London UK	United Kingdom	1979	1979	Hospital_environment	Hospital_enviroment	1	IC1	NA	99.7	97.6	ADC-11
Chr_830_1	SAMN54724560	830	Missing	Missing	Missing	Missing	Missing	Missing	1	IC1	NA	99.6	97.6	ADC-11
Chr_20_1	SAMN54724344	20	CHU Bichat Paris, France	France	1993	1993	Clinical_sample	Clinical_sample	1	IC1	NA	99.7	97.6	ADC-11
Chr_15_1	SAMN54724341	15	Dordrecht, Netherlands	Netherlands	1984	1984	Urinary_tract	Urinary_system	new_alleles	NA	NA	99.7	97.6	ADC-11
Chr_642_1	SAMN54724496	642	Singapore	Singapore	1998	1998	Missing	Missing	1	IC1	NA	99.7	97.6	ADC-191

Table S2 Bacterial genome metadata from publicly available genomes. Multi-locus sequence typing (MLST) was performed according to the Pasteur scheme, and assignment to international clones was conducted using existing data as stated in Methods.

Genome ID	GenBank accession	Location	Parsed location	Collection date	Parsed collection date	Isolation source	Parsed isolation source	Sequence type	International clone	IC2 Group	Total plasmids	Plasmid resistance genes	Plasmid virulence factors	ADC gene(s)
GCA_007107525.1_ASM710752v1_genomic	CP041587.1	Australia: Westmead, Sydney	Australia	1999	1999	Missing	Missing	49	Not IC	NA	3	ANT(2'')-Ia # sul2 # floR # ADC-176 # mphE # msrE	NA	ADC-258
GCA_050032045.1_ASM5003204v1_genomic	CP188281.1	China:Nanchang	China	2022-06	2022.4	sputum	Respiratory_tract	164	IC11	NA	5	NA	NA	ADC-199 # ADC-52
GCA_024205285.1_ASM2420528v1_genomic	CP088895.1	China:Hangzhou	China	2021-01-19	2021.1	patient	Hospital_enviroment	164	IC11	NA	5	NA	NA	ADC-199 # ADC-52
GCA_050031255.1_ASM5003125v1_genomic	CP188269.1	China:Nanchang	China	2024-01	2024.0	sputum	Respiratory_tract	164	IC11	NA	5	NA	NA	ADC-199 # ADC-52
GCA_040801815.1_ASM4080181v1_genomic	CP161817.1	China: Hangzhou	China	2021-05-04	2021.3	Homo sapiens	Missing	164	IC11	NA	5	NA	NA	ADC-199 # ADC-52
GCA_024205265.1_ASM2420526v1_genomic	CP088894.1	China:Hangzhou	China	2021-01-14	2021.0	patient	Hospital_enviroment	164	IC11	NA	0	NA	NA	ADC-199 # ADC-52
GCA_050032545.1_ASM5003254v1_genomic	CP188287.1	China:Nanchang	China	2022-02	2022.1	sputum	Respiratory_tract	164	IC11	NA	3	NA	NA	ADC-199 # ADC-52
GCA_047226445.1_ASM4722644v1_genomic	CP178406.1	China: Hangzhou	China	2022-02-14	2022.1	stool	Digestive_system	164	IC11	NA	5	NA	NA	ADC-199 # ADC-52

GCA_047227645.1_ASM4722764v1_genomic	CP178418.1	China: Hangzhou	China	2022-02-17	2022.1	stool	Digestive_system	164	IC11	NA	5	NA	NA	ADC-199 # ADC-52
GCA_047226455.1_ASM4722645v1_genomic	CP178412.1	China: Hangzhou	China	2022-02-17	2022.1	stool	Digestive_system	164	IC11	NA	5	NA	NA	ADC-199 # ADC-52
GCA_042143465.1_Lv371_genomic	CP169811.1	France	France	2016	2016	Urinary source	Urinary_system	164	IC11	NA	7	floR # sul2 # APH(3'')-Ib # APH(6)-Id # mphE # msrE # APH(3')-VIa # OXA-58 # sul1 # qacEdelta1 # AAC(6')-Ib9 # arr-3 # dfrA19 # sul1 # qacEdelta1 # CARB-2 # tet(39) # tet(B) # sul2 # tetR	NA	ADC-199 # ADC-52
GCA_029713805.1_ASM2971380v1_genomic	CP121570.1	Saudi Arabia:Riyadh	Saudi Arabia	2019	2019	Missing	Missing	164	IC11	NA	6	tet(39) # ANT(2'')-Ia	NA	ADC-199 # ADC-52
GCA_041154825.1_ASM4115482v1_genomic	AP031581.1	Nepal:Kathmandu	Nepal	2019	2019	Missing	Missing	164	IC11	NA	0	NA	NA	ADC-199 # ADC-52
GCA_018135585.1_AS	CP073060.1	China:Hangzhou	China	2019-08-27	2019.7	patient	Hospital_enviroment	221	Not IC	NA	1	NA	NA	ADC-248

M1813558v1_genomic														
GCA_033190015.1_ASM3319001v1_genomic	CP136172.1	France: Marcy l'etoile	France	2021-11-18	2021.9	oral swab	Respiratory_tract	2247	Not IC	NA	0	NA	NA	NA
GCA_037939845.1_ASM3793984v1_genomic	CP149800.1	China: shijiazhuang, Shanxi Norman Bethune Hospital	China	2021-04-13	2021.3	Missing	Missing	1336	Not IC	NA	2	OXA-23	NA	ADC-165
GCA_000746605.1_ASM74660v1_genomic	CP009256.1	Canada:Toronto	Canada	2010-01-01	2010.0	hospital	Hospital_enviroment	638	Not IC	NA	0	NA	NA	ADC-203
GCA_012931625.1_ASM1293162v1_genomic	CP051869.1	Ghana: Sekondi-Takoradi	Ghana	2016-03	2016.2	cerebrospinal fluid	CNS	103	Not IC	NA	5	dfrA1 # mphE # msrE # AAC(3)-IId # OXA-23	NA	ADC-203
GCA_012931645.1_ASM1293164v1_genomic	CP051875.1	Ghana: Sekondi-Takoradi	Ghana	2016-01	2016.0	sputum	Respiratory_tract	103	Not IC	NA	4	dfrA1 # mphE # msrE # AAC(3)-IId # OXA-23	NA	ADC-203
GCA_024749525.1_ASM2474952v1_genomic	CP087312.1	Germany	Germany	Missing	Missing	Missing	Missing	103	Not IC	NA	4	sul2 # tet(B) # APH(6)-Id # APH(3'')-Ib # APH(3')-VIa # ANT(2'')-Ia # tetR	NA	ADC-203
GCA_050032335.1_ASM5003233v1_genomic	CP188291.1	China:Nanchang	China	2022-02	2022.1	sputum	Respiratory_tract	773	Not IC	NA	2	NA	NA	ADC-203

GCA_050320275.1_Up398_genomic	CP190363.1	Germany	Germany	2019	2019	Urinary source	Urinary_system	103	Not IC	NA	0	NA	NA	ADC-203
GCA_025854095.1_ASM2585409v1_genomic	CP107599.1	USA	United States	2018-08-14	2018.6	skin	Skin	16	Not IC	NA	1	NA	NA	ADC-169
GCA_025854255.1_ASM2585425v1_genomic	CP107616.1	USA	United States	2018-08-14	2018.6	ENVIRONMENT	ENVIRONMENT	16	Not IC	NA	1	NA	NA	ADC-169
GCA_025854035.1_ASM2585403v1_genomic	CP107593.1	USA	United States	2018-08-14	2018.6	skin	Skin	16	Not IC	NA	1	NA	NA	ADC-169
GCA_013394285.1_ASM1339428v1_genomic	CP044519.1	China:Guangdong	China	2017-06	2017.4	feces	Digestive_system	16	Not IC	NA	2	APH(6)-Id # AAC(3)-IIe # TEM-2 # msrE # mphE # APH(3')-Ia # sul2 # APH(3'')-Ib # APH(6)-Id # dfrA1 # floR # tet(X5) # tet(39) # APH(6)-Id # APH(3'')-Ib # sul2	NA	ADC-169
GCA_022811905.1_ASM2281190v1_genomic	CP065051.1	China: Guangzhou,Guangdong	China	2016-10-24	2016.8	balf	Respiratory_tract	16	Not IC	NA	1	dfrA19 # arr-3 # AAC(6')-Ib9 # qacEdelta1 # sul1 # APH(3'')-Ib #	NA	ADC-169

												APH(6)-Id # mphE # msrE # tet(39) # floR # tet(Y)		
GCA_050164325.1_ASM5016432v1_genomic	CP181857.1	Germany	Germany	2022	2022	Missing	Missing	25	IC7	NA	2	AAC(6')-Ian # APH(3'')-Ib # APH(6)-Id # sul2 # arr-2 # cmlA5 # sul1 # PER-7 # sul1 # armA # msrE # mphE # sul2 # tet(B) # tetR	NA	ADC-342
GCA_050164075.1_ASM5016407v1_genomic	CP181854.1	Germany	Germany	2022	2022	Missing	Missing	25	IC7	NA	2	AAC(6')-Ian # APH(3'')-Ib # APH(6)-Id # sul2 # arr-2 # cmlA5 # sul1 # PER-7 # sul1 # armA # msrE # mphE # sul2 # tet(B) # tetR	NA	ADC-342

GCA_041154865.1_ASM4115486v1_genomic	AP031586.1	Nepal:Kathmandu	Nepal	2019	2019	Missing	Missing	NA	NA	NA	1	armA # sul1 # PER-7 # sul1 # qacEdelta1 # cmlA5 # arr-2 # APH(6)-Id # APH(3'')-Ib # AAC(6')-Ian # tet(B) # mphE # msrE # tetR	NA	ADC-342
GCA_012934905.1_ASM1293490v1_genomic	CP050385.1	India	India	2019	2019	blood	Blood	25	IC7	NA	2	AAC(6')-Ian # OXA-23 # APH(3'')-Ib # APH(6)-Id # sul2 # sul2 # tet(B) # mphE # msrE # armA # sul1 # PER-7 # sul1 # cmlA5 # arr-2 # tetR	NA	ADC-342
GCA_005518095.1_ASM551809v1_genomic	CP040259.1	India	India	2018	2018	Pus	Skin	25	IC7	NA	3	sul2 # tet(B) # mphE # msrE # armA # sul1 #	NA	ADC-342

												PER-7 # sul1 # cmlA5 # arr-2 # sul2 # APH(6)-Id # APH(3'')-Ib # AAC(6')-Ian # tetR		
GCA_002082785.1_ASM208278v1_genomic	CP020597.1	South Korea: Busan	Korea, Republic of	2013-10	2013.8	sputum	Respiratory_tract	25	IC7	NA	1	armA # msrE # mphE # tet(B) # sul2 # AAC(6')-Ian # AAC(3)-IIe # sul2 # arr-2 # cmlA5 # qacEdelta1 # sul1 # PER-7 # sul1 # tetR	NA	ADC-342
GCA_038442565.1_CIRI-F15-02_genomic	CP151713.1	France: Marcy l'etoile	France	2015-02	2015.1	urine	Urinary_system	25	IC7	NA	1	AAC(6')-Ian # AAC(3)-IIe # APH(3'')-Ib # APH(6)-Id # tet(B) # sul2 # tetR	NA	ADC-342
GCA_038442305.1_CIRI-F19-10-9_genomic	CP151699.1	France: Marcy l'etoile	France	2019-10	2019.8	urine	Urinary_system	25	IC7	NA	0	NA	NA	ADC-342

GCA_038442395.1_CIRI-F19-10-2_genomic	CP151704.1	France: Marcy l'etoile	France	2019-10	2019.8	urine	Urinary_system	25	IC7	NA	0	NA	NA	ADC-342
GCA_038442275.1_CIRI-F19-10-CH3_genomic	CP151694.1	France: Marcy l'etoile	France	2019-10	2019.8	urine	Urinary_system	25	IC7	NA	1	AAC(6')-Ian # AAC(3)-IIe # APH(3'')-Ib # APH(6)-Id # tet(B) # sul2 # tetR	NA	ADC-342
GCA_038442235.1_CIRI-F19-10-CH8_genomic	CP151690.1	France: Marcy l'etoile	France	2019-10	2019.8	urine	Urinary_system	25	IC7	NA	1	AAC(6')-Ian # AAC(3)-IIe # APH(3'')-Ib # APH(6)-Id # tet(B) # sul2 # tetR	NA	ADC-342
GCA_038442585.1_CIRI-F15-01A_genomic	CP151717.1	France: Marcy l'etoile	France	2015-01	2015.0	urine	Urinary_system	25	IC7	NA	1	AAC(6')-Ian # AAC(3)-IIe # APH(3'')-Ib # APH(6)-Id # tet(B) # sul2 # tetR	NA	ADC-342
GCA_038442415.1_CIRI-F18-02_genomic	CP151707.1	France: Marcy l'etoile	France	2018-02	2018.1	urine	Urinary_system	25	IC7	NA	1	AAC(3)-IIe # AAC(6')-Ian # sul2 # tet(B) # APH(6)-Id # APH(3'')-Ib # tetR	NA	ADC-342

GCA_038442405.1_CIRI-F19-02_genomic	CP151705.1	France: Marcy l'etoile	France	2019-02	2019.1	urine	Urinary_system	25	IC7	NA	1	AAC(3)-IIe # sul2 # tet(B) # APH(6)-Id # APH(3'')-Ib # tetR	NA	ADC-342
GCA_038442555.1_CIRI-F15-06_genomic	CP151711.1	France: Marcy l'etoile	France	2015-06	2015.4	urine	Urinary_system	25	IC7	NA	1	AAC(6')-Ian # AAC(3)-IIe # APH(3'')-Ib # APH(6)-Id # tet(B) # sul2 # tetR	NA	ADC-342
GCA_038442575.1_CIRI-F15-01B_genomic	CP151715.1	France: Marcy l'etoile	France	2015-01	2015.0	urine	Urinary_system	25	IC7	NA	1	AAC(6')-Ian # AAC(3)-IIe # APH(3'')-Ib # APH(6)-Id # tet(B) # sul2 # tetR	NA	ADC-342
GCA_038442285.1_CIRI-F19-10-MH12_genomic	CP151696.1	France: Marcy l'etoile	France	2019-10	2019.8	urine	Urinary_system	25	IC7	NA	0	NA	NA	ADC-342
GCA_038442375.1_CIRI-F19-10-8_genomic	CP151700.1	France: Marcy l'etoile	France	2019-10	2019.8	urine	Urinary_system	25	IC7	NA	1	AAC(6')-Ian # AAC(3)-IIe # sul2 # tet(B) # APH(6)-Id # APH(3'')-Ib # tetR	NA	ADC-342
GCA_038442265.1_CIRI-F19-10-	CP151692.1	France: Marcy l'etoile	France	2019-10	2019.8	urine	Urinary_system	25	IC7	NA	1	AAC(3)-IIe # AAC(6')-Ian #	NA	ADC-342

CH6_genomic												tet(B) # APH(6)-Id # APH(3'')-Ib # tetR		
GCA_038442295.1_CIRI-F19-10-MH6_genomic	CP151697.1	France: Marcy l'etoile	France	2019-10	2019.8	urine	Urinary_system	25	IC7	NA	1	sul2 # tet(B) # APH(6)-Id # APH(3'')-Ib # AAC(3)-IIe # AAC(6')-Ian # tetR	NA	ADC-342
GCA_038442385.1_CIRI-F19-10-4_genomic	CP151702.1	France: Marcy l'etoile	France	2019-10	2019.8	urine	Urinary_system	25	IC7	NA	1	AAC(6')-Ian # AAC(3)-IIe # APH(3'')-Ib # APH(6)-Id # tet(B) # sul2 # tetR	NA	ADC-342
GCA_038442425.1_CIRI-F16-05_genomic	CP151709.1	France: Marcy l'etoile	France	2016-05	2016.3	urine	Urinary_system	25	IC7	NA	1	sul2 # tet(B) # APH(6)-Id # APH(3'')-Ib # AAC(3)-IIe # AAC(6')-Ian # tetR	NA	ADC-342
GCA_038442635.1_CIRI-F14-11_genomic	CP151719.1	France: Marcy l'etoile	France	2014-11	2014.8	urine	Urinary_system	25	IC7	NA	1	AAC(3)-IIe # AAC(6')-Ian # sul2 # tet(B) # APH(6)-Id # APH(3'')-Ib # tetR	NA	ADC-342
GCA_038442225.1_CIRI-	CP151688.1	France	France	2015	2015	urine	Urinary_system	25	IC7	NA	1	AAC(3)-IIe # AAC(6')-Ian # APH(3'')-	NA	ADC-342

40288_genomic												Ib # APH(6)-Id # tet(B) # sul2 # tetR		
GCA_019457715.1_ASM1945771v1_genomic	CP077801.1	France	France	2015	2015	urine	Urinary_system	25	IC7	NA	1	sul2 # tet(B) # APH(6)-Id # APH(3'')-Ib # AAC(6')-Ian # AAC(3)-IIe # tetR	NA	ADC-342
GCA_003006035.1_ASM300603v1_genomic	CP027530.1	Missing	Missing	Missing	Missing	Missing	Missing	25	IC7	NA	2	sul2 # tet(B) # APH(6)-Id # APH(3'')-Ib # AAC(3)-IIe # AAC(6')-Ian # NDM-1 # APH(3')-VIa # tetR	NA	ADC-342
GCA_042153585.1_Up377_genomic	CP169872.1	Argentina	Argentina	2018	2018	tracheal aspirate	Respiratory_tract	25	IC7	NA	4	AAC(3)-IIe # AAC(6')-Ian # APH(3'')-Ib # APH(6)-Id # tet(B) # sul2 # tetR	NA	ADC-259
GCA_042140285.1_Hv373_genomic	CP169768.1	Belgium	Belgium	2017	2017	Urinary source	Urinary_system	25	IC7	NA	2	ANT(2'')-Ia # ANT(2'')-Ia # ANT(2'')-Ia # ANT(2'')-Ia	NA	ADC-259 # ADC-80 # ADC-259

GCA_006843645.1_ASM684364v1_genomic	CP032215.1	Argentina: Buenos Aires, San Andres, Hospital Zonal General de Agudos "General Manuel Belgrano", Zip B1604BSB	Argentina	2016	2016	urine	Urinary_system	25	IC7	NA	5	APH(6)-Id # APH(3'')-Ib # AAC(3)-IIe # tetR	NA	ADC-259
GCA_042142835.1_Lv347_genomic	CP169809.1	Bolivia	Bolivia	2016	2016	Respiratory/sputum	Respiratory_tract	25	IC7	NA	1	AAC(3)-IIe # AAC(6')-Ian # APH(3'')-Ib # APH(6)-Id # tet(B) # sul2 # tetR	NA	ADC-259
GCA_042140455.1_Hv455_genomic	CP169776.1	Missing	Missing	Missing	Missing	Missing	Missing	new_alleles	NA	NA	2	APH(3'')-Ib # AAC(6')-Ian # AAC(3)-IIe # sul2 # tet(B) # tetR	NA	ADC-259
GCA_024426665.1_ASM2442666v1_genomic	CP098521.1	Lebanon	Lebanon	2012	2012	urine	Urinary_system	25	IC7	NA	1	sul2 # tet(B) # APH(6)-Id # APH(3'')-Ib # AAC(3)-IIe # AAC(6')-Ian # tetR	NA	ADC-342
GCA_018831125.1_AS	CP048131.1	Australia: Sydney	Australia	2010	2010	mid stream urine	Urinary_system	25	IC7	NA	4	ANT(2'')-Ia # APH(3')-	NA	ADC-88

M1883112v1_genomic												VIa # OXA-23 # tet(B) # sul2 # mphE # msrE # APH(6)-Id # APH(3'')-Ib # tetR		
GCA_030413705.1_ASM3041370v1_genomic	CP129245.1	France: Nancy	France	2013	2013	urine	Urinary_system	25	IC7	NA	4	ANT(2'')-Ia # NDM-9 # APH(3'')-Ib # APH(6)-Id # sul2	NA	ADC-342
GCA_003431385.1_ASM343138v1_genomic	CP022283.1	Mexico: Monterrey, Nuevo Leon	Mexico	2006-07-05	2006.5	bronchoalveolar lavage fluid	Respiratory_tract	25	IC7	NA	2	APH(6)-Id # APH(3'')-Ib	NA	ADC-174
GCA_035937495.1_ASM3593749v1_genomic	CP142393.1	Pakistan: Punjab	Pakistan	2017-01	2017.0	endotracheal tube	Respiratory_tract	307	IC7	NA	0	NA	NA	ADC-96
GCA_050039885.1_ASM5003988v1_genomic	CP188770.1	Missing	Missing	Missing	Missing	Missing	Missing	25	IC7	NA	2	sul2 # tet(B) # APH(6)-Id # APH(3'')-Ib # tetR	NA	ADC-342
GCA_018808925.1_ASM1880892v1_genomic	CP048849.1	Australia: Sydney	Australia	2006	2006	wound	Skin	25	IC7	NA	2	sul2 # APH(6)-Id # APH(3'')-Ib	NA	ADC-342
GCA_021899715.1_ASM2189971v1_genomic	CP091596.1	Germany	Germany	2018-02-18	2018.1	Missing	Missing	25	IC7	NA	3	APH(3'')-Ib # APH(6)-Id # sul2 # APH(3')-VIa # APH(3')-VIa #	NA	ADC-342

												ANT(2'')-Ia # ANT(2'')-Ia		
GCA_014322245.1_ASM1432224v1_genomic	CP047973.1	China:Hangzhou	China	2019-08-06	2019.6	rectal swab	Digestive_system	138	Not IC	NA	3	AAC(3)-IId # OXA-58 # sul2 # aadT # msrE # mphE # tet(39)	NA	ADC-156
GCA_002504145.1_ASM250414v1_genomic	CP018332.1	China:Hainan	China	2010	2010	sputum	Respiratory_tract	138	Not IC	NA	2	NA	NA	ADC-156
GCA_045278695.1_ASM4527869v1_genomic	CP137055.1	South Korea: Republic of	Korea, Republic of	2019-04-04T08:39Z	2019.3	Seoul National University Bundang Hospital Intensive Care Unit	Hospital_enviroment	756	Not IC	NA	1	NA	NA	ADC-156
GCA_024749425.1_ASM2474942v1_genomic	CP087325.1	Germany	Germany	Missing	Missing	Missing	Missing	193	Not IC	NA	2	NA	NA	ADC-156
GCA_045182155.1_ASM4518215v1_genomic	CP174007.1	USA	United States	2024-09-05	2024.7	urine	Urinary_system	132	IC10	NA	5	NA	NA	ADC-156
GCA_019997865.1_ASM1999786v1_genomic	CP083181.1	China:Yangzhou	China	2017-07-05	2017.5	sputum	Respiratory_tract	132	IC10	NA	1	NA	NA	ADC-156
GCA_024749405.1_ASM2474940v1_genomic	CP087328.1	Germany	Germany	Missing	Missing	Missing	Missing	33	IC10	NA	2	NA	NA	ADC-156
GCA_014159355.1_AS	AP022077.1	Japan:Tokyo	Japan	2018-02-13	2018.1	Missing	Missing	213	IC10	NA	0	NA	NA	ADC-156

M1415935v1_genomic														
GCA_001517645.1_R2091_genomic	LN997846.1	France	France	2014	2014	Missing	Missing	126	Not IC	NA	1	NA	NA	ADC-271
GCA_001457535.1_CIP70.10_genomic	LN865143.1	USA	United States	1950	1950	Missing	Missing	126	Not IC	NA	1	NA	NA	ADC-271
GCA_023809705.1_ASM2380970v1_genomic	CP098795.1	India:Mumbai	India	2020	2020	blood	Blood	126	Not IC	NA	4	AAC(3)-IId # mphE # msrE # APH(3')-VIa # OXA-58 # qacG # sul1 # qacEdelta1 # CARB-2 # NDM-1 # adeH # adeF # adeG # adeL # abeS	adeH # adeG # adeF # gspE2 # gspL # gspM	ADC-271
GCA_023809625.1_ASM2380962v1_genomic	CP098791.1	India:Mumbai	India	2020	2020	blood	Blood	126	Not IC	NA	3	AAC(3)-IId # mphE # msrE # APH(3')-VIa # OXA-58 # qacG # sul1 # qacEdelta1 # CARB-2 # NDM-1	NA	ADC-271
GCA_014874575.1_AS	CP059300.1	Malaysia: Kuala	Malaysia	2016	2016	blood	Blood	126	Not IC	NA	4	aadT # AAC(3)-IId # sul2 #	NA	ADC-271

M1487457v 1_genomic		Terengganu										NDM-1 # APH(3'')-Ib # APH(6)-Id # OXA-58 # msrE # mphE		
GCA_003012895.3_ASM301289v3_genomic	CP027704.2	India: Anantpur, Andhra Pradesh	India	2005	2005	soil	Soil	new_alleles	NA	NA	5	NA	NA	NA
GCA_042474915.1_ASM4247491v1_genomic	CP132915.1	China	China	2010	2010	sputum	Respiratory_tract	152	Not IC	NA	4	NDM-1 # APH(3')-VIa	NA	ADC-32
GCA_025264005.1_ASM2526400v1_genomic	CP103338.1	China:Jiangsu	China	2022-04-20	2022.3	Missing	Missing	2266	Not IC	NA	6	NA	NA	ADC-32
GCA_001077965.2_ASM107796v2_genomic	CP021347.1	India: Vellore	India	2015-03-15	2015.2	blood	Blood	1549	Not IC	NA	1	NA	NA	ADC-32
GCA_012931605.1_ASM1293160v1_genomic	CP051862.1	Ghana: Sekondi-Takoradi	Ghana	2016-01	2016.0	blood	Blood	1472	Not IC	NA	3	APH(6)-Id # APH(3'')-Ib # sul2 # APH(3')-Ia # floR # OXA-58	NA	ADC-32
GCA_033192035.1_ASM3319203v1_genomic	CP136183.1	France: Marcy l'etoile	France	2021-05-18	2021.4	oral swab	Respiratory_tract	2532	Not IC	NA	3	NA	NA	ADC-277
GCA_019703285.1_ASM1970328v1_genomic	AP024802.1	Japan	Japan	2016-07-16	2016.5	Missing	Missing	new_alleles	NA	NA	11	NA	NA	ADC-310
GCA_008931365.1_AS	CP042556.1	Australia: Sydney	Australia	2013-02-08	2013.1	Room 7	Missing	1547	Not IC	NA	8	qacG	NA	ADC-243

M893136v1_genomic														
GCA_003812065.1_ASM381206v1_genomic	CP033754.1	Missing	Missing	Missing	Missing	clinical isolate	Clinical_sample	1542	Not IC	NA	3	NA	NA	ADC-32
GCA_019903235.1_ASM1990323v1_genomic	CP061705.1	Finland	Finland	Missing	Missing	paper pulp mill	Paper_production	649	Not IC	NA	8	sul2 # MCR-4.7	NA	ADC-154
GCA_019076685.1_ASM1907668v1_genomic	CP060994.1	Malaysia	Malaysia	2018-10-30	2018.8	Faecal	Digestive_system	142	Not IC	NA	2	NA	NA	ADC-238
GCA_029223565.1_ASM2922356v1_genomic	CP104295.1	China: Hangzhou	China	2019-09-22	2019.7	Missing	Missing	46	Not IC	NA	1	NA	NA	ADC-342
GCA_023517235.1_ASM2351723v1_genomic	CP077835.1	China: Hangzhou, Sir Run Run Shaw Hospital ICU	China	2019-10-08	2019.8	Rectal swab_P87_BU24	Digestive_system	46	Not IC	NA	1	NA	NA	ADC-342
GCA_029774195.1_ASM2977419v1_genomic	CP104297.1	China: Hangzhou	China	2021-12-20	2021.9	Missing	Missing	46	Not IC	NA	2	OXA-23	NA	ADC-342
GCA_017724215.1_ASM1772421v1_genomic	CP072290.1	India: New Delhi	India	2020	2020	Respiratory specimen	Respiratory_tract	622	Not IC	NA	4	mphE # msrE # armA # sul1 # PER-7 # sul1 # cmlA5 # arr-2 # sul2 # APH(6)-Id # APH(3'')-Ib #	NA	ADC-342

												APH(3')-VIa # APH(3')-VIa		
GCA_017726555.1_ASM1772655v1_genomic	CP072300.1	India: New Delhi	India	2020	2020	Respiratory specimen	Respiratory_tract	622	Not IC	NA	4	mphE # msrE # armA # sul1 # PER-7 # sul1 # cmlA5 # arr-2 # sul2 # APH(6)-Id # APH(3'')-Ib # APH(3')-VIa	NA	ADC-342
GCA_017724115.1_ASM1772411v1_genomic	CP072275.1	India: New Delhi	India	2020	2020	Respiratory specimen	Respiratory_tract	622	Not IC	NA	4	mphE # msrE # armA # sul1 # PER-7 # sul1 # cmlA5 # arr-2 # sul2 # APH(6)-Id # APH(3'')-Ib # APH(3')-VIa # APH(3')-VIa	NA	ADC-342
GCA_017723975.1_ASM1772397v1_genomic	CP072270.1	India: New Delhi	India	2020	2020	Respiratory specimen	Respiratory_tract	622	Not IC	NA	4	mphE # msrE # armA # sul1 # PER-7 # sul1 #	NA	ADC-342

												cmlA5 # arr-2 # sul2 # APH(6)-Id # APH(3'')-Ib # APH(3')-VIa		
GCA_017724155.1_ASM1772415v1_genomic	CP072280.1	India: New Delhi	India	2020	2020	Respiratory specimen	Respiratory_tract	622	Not IC	NA	4	mphE # msrE # armA # sul1 # PER-7 # sul1 # cmlA5 # arr-2 # sul2 # APH(6)-Id # APH(3'')-Ib # OXA-23 # APH(3')-VIa	NA	ADC-342
GCA_017726495.1_ASM1772649v1_genomic	CP072295.1	India: New Delhi	India	2020	2020	Respiratory specimen	Respiratory_tract	622	Not IC	NA	4	mphE # msrE # armA # sul1 # PER-7 # sul1 # cmlA5 # arr-2 # sul2 # APH(6)-Id # APH(3'')-Ib # OXA-23 # APH(3')-VIa # APH(3')-VIa	NA	ADC-342

GCA_017639875.1_ASM1763987v1_genomic	CP072122.1	India: New Delhi	India	2020	2020	Respiratory specimen	Respiratory_tract	622	Not IC	NA	4	mphE # msrE # armA # sul1 # PER-7 # sul1 # cmlA5 # arr-2 # sul2 # APH(6)-Id # APH(3'')-Ib # OXA-23 # APH(3')-VIa # APH(3')-VIa	NA	ADC-342
GCA_017742855.1_ASM1774285v1_genomic	CP072398.1	India: New Delhi	India	2020	2020	Respiratory specimen	Respiratory_tract	622	Not IC	NA	4	mphE # msrE # armA # sul1 # PER-7 # sul1 # cmlA5 # arr-2 # sul2 # APH(6)-Id # APH(3'')-Ib # OXA-23 # APH(3')-VIa # APH(3')-VIa	NA	ADC-342
GCA_017724195.1_ASM1772419v1_genomic	CP072285.1	India: New Delhi	India	2020	2020	Respiratory specimen	Respiratory_tract	622	Not IC	NA	4	mphE # msrE # armA # sul1 # PER-7 # sul1 #	NA	ADC-342

												cmlA5 # arr-2 # sul2 # APH(6)-Id # APH(3'')-Ib # OXA-23 # APH(3')-VIa		
GCA_041154845.1_ASM4115484v1_genomic	AP031583.1	Nepal:Kathmandu	Nepal	2019	2019	Missing	Missing	new_alleles	NA	NA	1	APH(3'')-Ib # APH(6)-Id # sul2 # arr-2 # cmlA5 # qacEdelta1 # sul1 # armA # msrE # mphE	NA	ADC-342
GCA_000828935.1_ASM82893v1_genomic	AP014649.1	Nepal: Kathmandu	Nepal	2013	2013	Missing	Missing	622	Not IC	NA	1	mphE # msrE # armA # sul1 # PER-7 # sul1 # qacEdelta1 # cmlA5 # arr-2 # sul2 # APH(6)-Id # APH(3'')-Ib	NA	ADC-342
GCA_005280435.1_ASM528043v1_genomic	CP040050.1	India	India	2019	2019	blood	Blood	622	Not IC	NA	2	APH(3'')-Ib # APH(6)-Id # sul2 # arr-2 # cmlA5 # sul1 #	NA	ADC-342

												PER-7 # armA # msrE # mphE # APH(3')-VIa		
GCA_045689805.1_ASM4568980v1_genomic	CP142642.1	South Korea	Korea, Republic of	2020-02-02	2020.1	human	human	149	Not IC	NA	2	NA	NA	ADC-342
GCA_041154855.1_ASM4115485v1_genomic	AP031585.1	Nepal:Kathmandu	Nepal	2019	2019	Missing	Missing	149	Not IC	NA	0	NA	NA	ADC-342
GCA_001077565.2_ASM107756v2_genomic	CP021345.1	India: Vellore	India	2014-04-14	2014.3	blood	Blood	149	Not IC	NA	3	APH(3')-VIa # APH(3'')-Ib # APH(6)-Id # sul2 # arr-2 # cmlA5 # sul1 # PER-7 # sul1 # armA # msrE # mphE	NA	ADC-342
GCA_008632635.1_ASM863263v1_genomic	CP043953.1	Malaysia	Malaysia	2017-12-10	2017.9	soil	Soil	46	Not IC	NA	1	OXA-72	NA	ADC-342
GCA_024749645.1_ASM2474964v1_genomic	CP087340.1	Germany	Germany	Missing	Missing	Missing	Missing	268	Not IC	NA	3	NA	NA	ADC-342
GCA_043050845.1_ASM4305084v1_genomic	CP134579.1	China: Tianjin	China	2010	2010	sputum	Respiratory_tract	990	Not IC	NA	2	NA	NA	ADC-342

GCA_043044125.1_ASM4304412v1_genomic	CP134555.1	China: Henan	China	2010	2010	sputum	Respiratory_tract	719	Not IC	NA	2	NA	NA	ADC-342
GCA_014672775.1_ASM1467277v1_genomic	CP059041.1	Missing	Missing	Missing	Missing	Missing	Missing	437	Not IC	NA	1	NA	NA	ADC-342
GCA_013372085.1_ASM1337208v1_genomic	CP053098.1	USA: San Diego	United States	2017-08	2017.6	Missing	Missing	437	Not IC	NA	2	NA	NA	ADC-342
GCA_000015425.1_ASM1542v1_genomic	CP000521.1	Missing	Missing	Missing	Missing	Missing	Missing	437	Not IC	NA	2	NA	NA	ADC-342
GCA_008630895.1_ASM863089v1_genomic	CP043910.1	Missing	Missing	Missing	Missing	Generated in lab from ATCC 17979	Lab	437	Not IC	NA	0	NA	NA	ADC-342
GCA_001593425.2_ASM159342v2_genomic	CP018664.1	Missing	Missing	Missing	Missing	blood	Blood	437	Not IC	NA	0	NA	NA	ADC-342
GCA_001941765.1_ASM194176v1_genomic	CP019034.1	Missing	Missing	Missing	Missing	lab mutation of ATCC 17978	Lab	437	Not IC	NA	0	NA	NA	ADC-342
GCA_029459615.1_ASM2945961v1_genomic	CP113074.1	Missing	Missing	Missing	Missing	Missing	Missing	437	Not IC	NA	2	NA	NA	ADC-342
GCA_001077675.1_ASM107767v1_genomic	CP012004.1	Canada	Canada	2014	2014	Missing	Missing	437	Not IC	NA	1	sul2	NA	ADC-342
GCA_004794235.2_ASM479423v2_genomic	CP039028.2	South Korea	Korea, Republic of	2018-01	2018.0	Missing	Missing	437	Not IC	NA	3	NA	pilM # pilN # pilO # pilP # pilQ	ADC-342

GCA_004797155.2_ASM479715v2_genomic	CP039025.2	South Korea	Korea, Republic of	2018-01	2018.0	Missing	Missing	437	Not IC	NA	2	NA	NA	ADC-342
GCA_004794275.2_ASM479427v2_genomic	CP039023.2	South Korea	Korea, Republic of	2018-01	2018.0	Missing	Missing	437	Not IC	NA	0	NA	NA	ADC-342
GCA_024134665.1_ASM2413466v1_genomic	CP099855.1	Missing	Missing	Missing	Missing	Missing	Missing	437	Not IC	NA	0	NA	NA	ADC-342
GCA_026167785.1_ASM2616778v1_genomic	CP033108.1	Missing	Missing	Missing	Missing	Missing	Missing	437	Not IC	NA	1	NA	NA	ADC-342
GCA_026167805.1_ASM2616780v1_genomic	CP033110.1	Missing	Missing	Missing	Missing	Missing	Missing	437	Not IC	NA	1	NA	NA	ADC-342
GCA_019356215.1_ASM1935621v1_genomic	CP079931.1	France	France	1951	1951	Missing	Missing	437	Not IC	NA	3	sul2	NA	ADC-342
GCA_020912005.1_ASM2091200v1_genomic	CP074710.1	Missing	Missing	2020-03	2020.2	Missing	Missing	437	Not IC	NA	5	sul2	NA	ADC-342
GCA_019316825.1_ASM1931682v1_genomic	CP079212.1	USA	United States	2018	2018	Missing	Missing	437	Not IC	NA	0	NA	NA	ADC-342
GCA_024134645.1_ASM2413464v1_genomic	CP099856.1	Missing	Missing	Missing	Missing	Missing	Missing	437	Not IC	NA	0	NA	NA	ADC-342
GCA_022467135.1_ASM2246713v1_genomic	CP091335.1	France	France	1951	1951	Missing	Missing	437	Not IC	NA	0	NA	NA	ADC-342

GCA_024505625.1_ASM2450562v1_genomic	CP101889.1	Brazil	Brazil	2021-03-31	2021.2	catheter tip	Urinary_system	new_alleles	NA	NA	0	NA	NA	ADC-342
GCA_017726575.1_ASM1772657v1_genomic	CP072305.1	India: New Delhi	India	2020	2020	Respiratory specimen	Respiratory_tract	374	Not IC	NA	2	sul2	NA	ADC-181
GCA_013416255.1_ASM1341625v1_genomic	CP053215.1	Tanzania: Dar es Salaam	Tanzania, United Republic of	2017-10	2017.8	Missing	Missing	374	Not IC	NA	2	sul2 # dfrA1 # SAT-2 # aadA # CARB-16 # APH(3')-Ia # ANT(2'')-Ia	NA	ADC-342
GCA_040930415.1_ASM4093041v1_genomic	CP162300.1	Singapore	Singapore	2010-11-17	2010.9	Missing	Missing	374	Not IC	NA	2	OXA-72	NA	ADC-342
GCA_004028375.1_ASM402837v1_genomic	CP034092.1	China: Hangzhou	China	2015-08-02	2015.6	sputum	Respiratory_tract	77	Not IC	NA	5	OXA-72	NA	ADC-342
GCA_014169655.1_ASM1416965v1_genomic	AP022238.1	Japan:Tokyo	Japan	2018-02-13	2018.1	Missing	Missing	1548	Not IC	NA	2	NA	NA	ADC-342
GCA_042152325.1_Up280_genomic	CP169869.1	USA	United States	Missing	Missing	Urinary source/catheter isolate	Urinary_system	342	Not IC	NA	0	NA	NA	ADC-270
GCA_033469325.1_ASM3346932v1_genomic	CP136173.1	France: Marcy l'etoile	France	2021-11-08	2021.9	hospital environment	Hospital_enviroment	578	Not IC	NA	4	CARB-16	NA	ADC-274
GCA_040703085.1_ASM4070308v1_genomic	CP160032.1	Egypt: Alexandria	Egypt	2023	2023	MiniBAL	Respiratory_tract	158	IC12	NA	2	OXA-23 # sul1 # qacEdelta1 # dfrA7 # AAC(6')-	NA	ADC-117

												Ib3 # GES-35 # APH(3')-VIa		
GCA_040543095.1_ASM4054309v1_genomic	CP159763.1	Egypt: Alexandria	Egypt	2023	2023	swab	Hospital_enviroment	158	IC12	NA	3	OXA-23 # sul1 # qacEdelta1 # dfrA7 # AAC(6')-Ib3 # GES-35 # APH(3')-VIa	NA	ADC-117
GCA_029713725.1_ASM2971372v1_genomic	CP121591.1	Saudi Arabia:Medina	Saudi Arabia	2019	2019	Missing	Missing	158	IC12	NA	3	sul1 # qacEdelta1 # dfrA7 # AAC(6')-Ib3 # GES-11 # APH(3')-VIa # sul1 # qacEdelta1 # dfrA7 # AAC(6')-Ib10 # msrE # mphE	NA	NA
GCA_022460155.2_ASM2246015v2_genomic	CP091372.1	Belgium	Belgium	2014	2014	Missing	Missing	158	IC12	NA	1	ANT(2'')-Ia # aadA2 # APH(3'')-Ib # APH(6)-Id # GES-12 # AAC(6')-Ib10 # dfrA7 # qacEdelta1 # sul1 # CARB-16	NA	ADC-117

GCA_029582015.1_ASM2958201v1_genomic	CP113069.1	Poland	Poland	2014	2014	Nestling, choana	White_stork	309	Not IC	NA	0	NA	NA	ADC-312
GCA_008124045.1_ASM812404v1_genomic	CP043180.1	Missing	Missing	2018-05-18	2018.4	mouse gut	Mouse	294	Not IC	NA	0	NA	NA	ADC-87
GCA_009833005.1_ASM983300v1_genomic	CP045428.1	Canada: Edmonton	Canada	2015	2015	Coccygeal isolate	Bone	825	Not IC	NA	0	NA	NA	ADC-325
GCA_022466975.1_ASM2246697v1_genomic	CP091334.1	Netherlands	Netherlands	1911	1911	Missing	Missing	52	Not IC	NA	0	NA	NA	ADC-158
GCA_019331655.1_ASM1933165v1_genomic	CP058289.1	Italy	Italy	2014-10-02	2014.8	Missing	Missing	52	Not IC	NA	2	NA	NA	ADC-158
GCA_025995075.1_ASM2599507v1_genomic	AP025740.1	Missing	Missing	Missing	Missing	Missing	Missing	52	Not IC	NA	0	NA	NA	ADC-158
GCA_014672755.1_ASM1467275v1_genomic	CP059040.1	Missing	Missing	Missing	Missing	Missing	Missing	52	Not IC	NA	0	NA	NA	ADC-158
GCA_022870045.1_ASM2287004v1_genomic	CP064375.1	Missing	Missing	Missing	Missing	Missing	Missing	52	Not IC	NA	0	NA	NA	ADC-158
GCA_016117795.1_ASM1611779v1_genomic	CP065887.1	Missing	Missing	Missing	Missing	Missing	Missing	52	Not IC	NA	2	NA	NA	ADC-158
GCA_020911985.1_ASM2091198v1_genomic	CP074585.1	Missing	Missing	2020-03	2020.2	Missing	Missing	52	Not IC	NA	1	NA	NA	ADC-158

GCA_002116925.1_ASM211692v1_genomic	CP015121.1	USA	United States	2015	2015	Missing	Missing	52	Not IC	NA	1	NA	NA	ADC-158
GCA_009035845.1_ASM903584v1_genomic	CP045110.1	USA:Georgia, Atlanta	United States	1948	1948	urine	Urinary_system	52	Not IC	NA	2	NA	NA	ADC-158
GCA_009759685.1_ASM975968v1_genomic	CP046654.1	Australia: Melbourne	Australia	2017-11	2017.8	Missing	Missing	52	Not IC	NA	1	NA	NA	ADC-158
GCA_034083075.1_ASM3408307v1_genomic	CP139208.1	China: Zhengzhou	China	2022-03-06	2022.2	throat swab	Respiratory_tract	52	Not IC	NA	1	NA	NA	ADC-158
GCA_022369355.1_ASM2236935v1_genomic	CP059474.1	China:Guangzhou	China	2018-12	2018.9	Manis javanica	Pangolin	52	Not IC	NA	2	tet(B) # sul2 # APH(3')-Ia # dfrA16 # CARB-2 # aadA3 # AAC(3)-IId # floR # tet(M) # tetR	NA	ADC-158
GCA_015910475.1_ASM1591047v1_genomic	CP065432.1	USA	United States	2016-05	2016.3	blood	Blood	438	Not IC	NA	2	NA	NA	ADC-158
GCA_024749605.1_ASM2474960v1_genomic	CP087331.1	Germany	Germany	Missing	Missing	Missing	Missing	54	Not IC	NA	3	sul2	NA	ADC-346
GCF_038627335.1_ASM3862733v2_genomic	NZ_CP156933.1	USA	United States	2024-04-17	2024.3	sputum	Respiratory_tract	78	IC6	NA	3	NA	NA	ADC-152
GCA_038627335.2_AS	CP156933.1	USA	United States	2024-04-17	2024.3	sputum	Respiratory_tract	78	IC6	NA	3	NA	NA	ADC-152

M3862733v 2_genomic														
GCF_03700 8745.1_AS M3700874v 2_genomic	NZ_CP1557 21.1	USA	United States	2024-02-20	2024.1	skin	Skin	78	IC6	NA	2	NA	NA	ADC-152
GCF_03742 9075.1_202 4CK-00278_geno mic	NZ_CP1554 55.1	USA	United States	2024-03-04	2024.2	Tracheal Aspirate/Was h	Respiratory_tract	78	IC6	NA	2	NA	NA	ADC-152
GCA_03742 9075.2_202 4CK-00278_geno mic	CP155455.1	USA	United States	2024-03-04	2024.2	Tracheal Aspirate/Was h	Respiratory_tract	78	IC6	NA	2	NA	NA	ADC-152
GCA_03700 8745.2_AS M3700874v 2_genomic	CP155721.1	USA	United States	2024-02-20	2024.1	skin	Skin	78	IC6	NA	2	NA	NA	ADC-152
GCA_02305 4035.2_AS M2305403v 2_genomic	CP115639.1	USA	United States	2022-03-26	2022.2	sputum	Respiratory_tract	78	IC6	NA	1	NA	NA	ADC-152
GCF_02305 4035.1_AS M2305403v 2_genomic	NZ_CP1156 39.1	USA	United States	2022	2022	Respiratory_tr act	Respiratory_tract	78	IC6	NA	1	NA	NA	ADC-152
GCA_02361 5345.2_AS M2361534v 2_genomic	CP115643.1	USA	United States	2022-04-28	2022.3	sputum	Respiratory_tract	78	IC6	NA	1	NA	NA	ADC-152
GCF_02361 5345.1_AS M2361534v 2_genomic	NZ_CP1156 43.1	USA	United States	2022	2022	Respiratory_tr act	Respiratory_tract	78	IC6	NA	1	NA	NA	ADC-152
GCF_02309 3135.1_AS M2309313v 2_genomic	NZ_CP1156 41.1	USA	United States	2022	2022	Respiratory_tr act	Respiratory_tract	78	IC6	NA	1	NA	NA	ADC-152

GCF_022916135.1_ASM2291613v2_genomic	NZ_CP115637.1	USA	United States	2022	2022	Respiratory_tract	Respiratory_tract	78	IC6	NA	1	NA	NA	ADC-152
GCA_023093135.2_ASM2309313v2_genomic	CP115641.1	USA	United States	2022-04-01	2022.2	sputum	Respiratory_tract	78	IC6	NA	1	NA	NA	ADC-152
GCA_022916135.2_ASM2291613v2_genomic	CP115637.1	USA	United States	2022-03-13	2022.2	sputum	Respiratory_tract	78	IC6	NA	1	NA	NA	ADC-152
GCF_024106315.1_ASM2410631v2_genomic	NZ_CP115645.1	USA	United States	2022	2022	Skin	Skin	78	IC6	NA	2	NA	NA	ADC-152
GCA_024106315.2_ASM2410631v2_genomic	CP115645.1	USA	United States	2022-06-06	2022.4	skin	Skin	78	IC6	NA	2	NA	NA	ADC-152
GCA_040273015.2_ASM4027301v2_genomic	CP167043.1	USA	United States	2024-06-04	2024.4	skin	Skin	78	IC6	NA	2	NA	NA	ADC-152
GCA_003949725.2_ASM394972v2_genomic	CP039930.1	USA: Arizona	United States	2012-03-06	2012.2	blood	Blood	78	IC6	NA	3	OXA-90	NA	ADC-152
GCA_003948085.2_ASM394808v2_genomic	CP039341.1	USA: Arizona	United States	2012-03-12	2012.2	trach asp	Respiratory_tract	78	IC6	NA	1	NA	NA	ADC-152
GCA_003949115.2_ASM394911v2_genomic	CP039343.1	USA: Arizona	United States	2012-03-06	2012.2	trach asp	Respiratory_tract	78	IC6	NA	1	NA	NA	ADC-152
GCA_022459755.2_ASM2245975v2_genomic	CP091369.1	Belgium	Belgium	2014	2014	Missing	Missing	78	IC6	NA	1	OXA-72	NA	ADC-152

GCA_016864335.2_ASM1686433v2_genomic	CP060505.1	France: Sens	France	2015-11	2015.8	Catheter	Urinary_system	NA	NA	NA	1	OXA-72	NA	ADC-152
GCA_016864915.1_ASM1686491v1_genomic	CP059729.1	France: Nice	France	2017-08	2017.6	Peritoneal fluid	Abdomen	78	IC6	NA	2	OXA-72 # OXA-72	NA	ADC-152
GCA_016864315.2_ASM1686431v2_genomic	CP060504.1	France: Cayenne	France	2017-04	2017.2	bronchoalveolar lavage	Respiratory_tract	78	IC6	NA	1	OXA-72	NA	ADC-152
GCA_022460295.1_ASM2246029v1_genomic	CP091373.1	Belgium	Belgium	Missing	Missing	Missing	Missing	78	IC6	NA	0	NA	NA	ADC-152
GCA_016864295.1_ASM1686429v1_genomic	CP060029.1	France: Paris	France	2015-02	2015.1	Throat sample	Respiratory_tract	1077	IC6	NA	0	NA	NA	ADC-336
GCA_019903175.1_ASM1990317v1_genomic	CP059546.1	USA	United States	Missing	Missing	field soil	Soil	647	Not IC	NA	0	NA	NA	ADC-95
GCA_019903195.1_ASM1990319v1_genomic	CP059547.1	USA	United States	Missing	Missing	field soil	Soil	647	Not IC	NA	0	NA	NA	ADC-95
GCA_001895125.2_ASM189512v2_genomic	AP023077.1	Missing	Missing	2015-12-19	2015.9	Missing	Missing	412	Not IC	NA	3	APH(3')-VIa # NDM-1 # OXA-58	NA	ADC-249
GCA_022466815.1_ASM2246681v1_genomic	CP091333.1	USA	United States	1944	1944	Plant microbiota	Plant	738	Not IC	NA	0	NA	NA	ADC-158
GCA_005280675.1_ASM528067v1_genomic	CP040080.1	India	India	2019	2019	sputum	Respiratory_tract	285	Not IC	NA	3	NA	NA	ADC-269

GCA_023094035.1_ASM2309403v1_genomic	CP095784.1	India: Bangalore	India	2021-02-15	2021.1	Sewage	Water	new_ST	NA	NA	0	NA	NA	ADC-271
GCA_019264805.1_ASM1926480v1_genomic	CP078525.1	Thailand: Nakhon Ratchasima	Thailand	Missing	Missing	food	food	2119	Not IC	NA	0	NA	NA	ADC-266
GCA_022369275.1_ASM2236927v1_genomic	CP058729.1	China:Guangzhou	China	2018-12	2018.9	Manis javanica	Pangolin	866	Not IC	NA	2	tet(B) # sul2 # tetR	NA	ADC-158
GCA_026062515.1_ASM2606251v1_genomic	CP110462.1	Australia: Brisbane	Australia	1999	1999	Missing	Missing	111	Not IC	NA	6	ANT(2'')-Ia # APH(3')-VIa # OXA-23 # OXA-23 # sul2 # tet(B) # APH(6)-Id # APH(3'')-Ib # tetR	NA	ADC-344
GCA_049177245.1_ASM4917724v1_genomic	CP185768.1	India: Vypin, Kochi, Kerala	India	2023-07-25	2023.6	shrimp	shrimp	new_ST	NA	NA	3	OXA-72	NA	ADC-167
GCA_033612615.1_ASM3361261v1_genomic	CP138324.1	South Korea	Korea, Republic of	2022	2022	Missing	Missing	155	Not IC	NA	1	NA	NA	ADC-167
GCA_049177235.1_ASM4917723v1_genomic	CP185767.1	India: Kalamukku, Kochi, Kerala	India	2023-07-17	2023.5	Clam	Clam	479	Not IC	NA	0	NA	NA	ADC-88
GCA_029909475.1_ASM2990947v1_genomic	CP123854.1	Canada	Canada	2021-11	2021.8	Feedlot Water bowl	Water	155	Not IC	NA	5	adeK # adeJ # adeI # OXA-695	pgaB # pgaA # pgaB # pgaA	ADC-158
GCA_029847455.2_AS	CP151078.1	Missing	Missing	2022-12-21	2022.9	Missing	Missing	new_alleles	NA	NA	1	NA	NA	ADC-159

M2984745v 2_genomic														
GCA_030168265.1_ASM3016826v1_genomic	CP096812.1	USA:Kansas	United States	2013-10-26	2013.8	Missing	Missing	274	Not IC	NA	0	NA	NA	ADC-68
GCA_000814345.1_ASM81434v1_genomic	CP010397.1	Colombia	Colombia	2012	2012	bodily fluid	Secretion	464	IC9	NA	3	APH(3')-VIa	NA	ADC-80
GCA_022459095.1_ASM2245909v1_genomic	CP091361.1	Belgium	Belgium	Missing	Missing	Missing	Missing	85	IC9	NA	0	NA	NA	ADC-176 # ADC-80 # ADC-339
GCA_004768705.1_ASM476870v1_genomic	CP038644.1	India	India	2018	2018	blood	Blood	85	IC9	NA	8	mphE # armA # sul1 # CARB-2 # OXA-420 # ANT(2'')-Ia # ANT(2'')-Ia # ANT(2'')-Ia	NA	ADC-80 # ADC-176
GCA_034422815.1_ASM3442281v1_genomic	CP139836.1	Germany	Germany	2017-05-12	2017.4	Missing	Missing	85	IC9	NA	2	OXA-23 # APH(3')-VIa	NA	ADC-80 # ADC-176
GCA_020132105.1_ASM2013210v1_genomic	CP082952.1	Lebanon	Lebanon	2015	2015	tracheal aspirate	Respiratory_tract	85	IC9	NA	1	NA	NA	ADC-158 # ADC-176
GCA_015832155.1_ASM1583215v1_genomic	CP065392.1	Spain: Bilbao	Spain	2019	2019	clinical	Clinical_sample	85	IC9	NA	2	ANT(2'')-Ia	NA	NA
GCA_022468985.2_ASM2246898v2_genomic	CP091356.1	Belgium	Belgium	2017	2017	Missing	Missing	85	IC9	NA	1	NA	NA	ADC-176 # ADC-80

														# ADC-176
GCA_024749465.1_ASM2474946v1_genomic	CP087309.1	Germany	Germany	Missing	Missing	Missing	Missing	85	IC9	NA	2	APH(3')-VIa # sul1 # qacEdelta1 # dfrA7 # AAC(6')-Ib3 # GES-11 # APH(6)-Id # APH(3'')-Ib # aadA3 # cmlA5 # ANT(2'')-Ia	NA	ADC-80
GCA_041014305.2_ASM4101430v1_genomic	CP160130.1	Egypt: Alexandria	Egypt	2023	2023	blood	Blood	85	IC9	NA	2	sul1 # qacEdelta1 # dfrA7 # AAC(6')-Ib3 # GES-11 # APH(3')-VIa # OXA-23	NA	ADC-80
GCA_042151085.1_Rp772_genomic	CP169859.1	France: Montferneil	France	2022-03-03	2022.2	Respiratory/Bronchial aspirate	Respiratory_tract	32	Not IC	NA	6	rsmA	NA	ADC-79
GCA_038441325.1_ASM3844132v1_genomic	CP133706.1	Netherlands	Netherlands	2021-05-11	2021.4	sputum	Respiratory_tract	32	Not IC	NA	5	mphE # msrE # OXA-97 # APH(6)-Id # APH(3'')-Ib # ANT(2'')-Ia # APH(3')-Ia # NDM-1 # aadT # sul2 # tet(X3)	NA	ADC-79

GCA_050320285.1_Up423_genomic	CP190364.1	USA	United States	2019	2019	urine	Urinary_system	32	Not IC	NA	5	OXA-24	NA	ADC-79
GCA_003547115.1_ASM354711v1_genomic	CP027183.1	Missing	Missing	Missing	Missing	Missing	Missing	32	Not IC	NA	4	OXA-58 # OXA-58 # OXA-58 # ANT(2'')-Ia	NA	ADC-79
GCA_003431865.1_ASM343186v1_genomic	CP027178.1	Missing	Missing	Missing	Missing	Missing	Missing	32	Not IC	NA	4	OXA-58 # OXA-58 # OXA-58 # ANT(2'')-Ia	NA	ADC-79
GCA_038442095.1_ASM3844209v1_genomic	CP133712.1	Netherlands	Netherlands	2021-03-18	2021.2	BAL	Respiratory_tract	32	Not IC	NA	4	APH(3')-Ia # ANT(2'')-Ia # APH(3'')-Ib # APH(6)-Id # OXA-97 # msrE # mphE # tet(X3) # sul2 # aadT # NDM-1 # qacG	NA	ADC-79
GCA_038442125.1_ASM3844212v1_genomic	CP133717.1	Netherlands	Netherlands	2021-02-23	2021.1	sputum	Respiratory_tract	32	Not IC	NA	2	NA	NA	ADC-79
GCA_024749625.1_ASM2474962v1_genomic	CP087335.1	Germany	Germany	Missing	Missing	Missing	Missing	32	Not IC	NA	4	NA	NA	NA
GCA_019264785.1_ASM1926478v1_genomic	CP078526.1	Thailand: Phra Nakhon Si Ayutthaya	Thailand	Missing	Missing	food	food	2563	Not IC	NA	0	NA	NA	ADC-166
GCA_001261895.2_R2090_genomic	LN868200.1	France	France	2013	2013	Missing	Missing	267	Not IC	NA	0	NA	NA	ADC-291

GCF_000186665.3_ASM18666v4_genomic	NC_020547.2	Australia	Australia	Missing	Missing	Missing	Missing	267	Not IC	NA	1	NA	NA	ADC-291
GCA_000186665.4_ASM18666v4_genomic	CP003967.2	Australia	Australia	Missing	Missing	Missing	Missing	267	Not IC	NA	1	NA	NA	ADC-291
GCA_049177225.1_ASM4917722v1_genomic	CP185766.1	India: kalamukku, Kochi, Kerala	India	2023-07-17	2023.5	fish	fish	2276	Not IC	NA	0	NA	NA	ADC-311
GCA_048750975.1_ACI_genomic	CP178250.1	Portugal: Coimbra	Portugal	2022	2022	experiment	Lab	new_alleles	NA	NA	0	NA	NA	ADC-206
GCA_048750995.1_A118_genomic	CP178253.1	Argentina: Buenos Aeres	Argentina	2010	2010	Blood culture	Blood	172	Not IC	NA	0	NA	NA	ADC-206
GCA_029459655.1_ASM2945965v1_genomic	CP113072.1	Missing	Missing	Missing	Missing	Missing	Missing	172	Not IC	NA	0	NA	NA	ADC-206
GCA_014672735.1_ASM1467273v1_genomic	CP059039.1	Missing	Missing	1995	1995	blood culture	Blood	172	Not IC	NA	0	NA	NA	ADC-206
GCA_029459755.1_ASM2945975v1_genomic	CP113071.1	Missing	Missing	Missing	Missing	Missing	Missing	172	Not IC	NA	0	NA	NA	ADC-206
GCA_029459635.1_ASM2945963v1_genomic	CP113073.1	Missing	Missing	Missing	Missing	Missing	Missing	172	Not IC	NA	0	NA	NA	ADC-206
GCA_029459775.1_ASM2945977v1_genomic	CP113070.1	Missing	Missing	Missing	Missing	Missing	Missing	172	Not IC	NA	0	NA	NA	ADC-206
GCA_024749445.1_AS	CP087298.1	Germany	Germany	Missing	Missing	Missing	Missing	400	Not IC	NA	1	APH(3')-VIa #	NA	ADC-347

M2474944v1_genomic												ANT(2'')-Ia # aadA2 # APH(3'')-Ib # APH(6)-Id # GES-14 # AAC(6')-Ib10 # dfrA7 # qacEdelta1 # sul1		
GCA_050320265.1_Up392_genomic	CP190362.1	USA: Indiana	United States	2018	2018	Urinary source	Urinary_system	810	Not IC	NA	0	NA	NA	ADC-283
GCA_012931665.1_ASM1293166v1_genomic	CP051866.1	Ghana: Sekondi-Takoradi	Ghana	2016-02	2016.1	sputum	Respiratory_tract	107	Not IC	NA	2	tet(39) # msrE # mphE # APH(3')-Ia # ANT(2'')-Ia # OXA-420 # APH(3'')-Ib # APH(6)-Id # sul2 # dfrA20	NA	ADC-87
GCA_034494405.1_ASM3449440v1_genomic	CP140420.1	USA	United States	2023-06-18	2023.5	blood	Blood	1142	Not IC	NA	4	OXA-23	NA	ADC-87
GCA_000069205.1_ASM6920v1_genomic	CU468230.2	Missing	Missing	Missing	Missing	Missing	Missing	17	Not IC	NA	3	NA	NA	NA
GCA_012272755.1_ASM1227275v1_genomic	CP050911.1	China: Beijing	China	2018	2018	sputum	Respiratory_tract	2	IC2	2	2	NA	NA	ADC-30
GCA_012272795.1_AS	CP050916.1	China: Beijing	China	2017	2017	stethoscope	Hospital_enviroment	2	IC2	2	2	NA	NA	ADC-30

M1227279v 1_genomic														
GCA_00180 6365.1_AS M180636v1 _genomic	CP017644.1	South Korea:Dae jeon	Korea, Republic of	2015-02-25	2015.2	Bronchial	Respiratory_tract	2	IC2	2	1	NA	NA	ADC-30
GCA_03318 1805.1_AS M3318180v 1_genomic	CP137066.1	South Korea: Republic of	Korea, Republic of	2016-03-16T16:24Z	2016.2	Seoul National University Bundang Hospital Intensive Care Unit	Hospital_envirome nt	2	IC2	2	2	OXA-23	NA	ADC-30
GCA_00180 6445.1_AS M180644v1 _genomic	CP017652.1	South Korea:Dae gu	Korea, Republic of	2015-08-28	2015.7	wound	Skin	2	IC2	2	1	NA	NA	ADC-30
GCA_00180 6425.1_AS M180642v1 _genomic	CP017650.1	South Korea:Dae gu	Korea, Republic of	2015-08-28	2015.7	blood	Blood	2	IC2	2	1	NA	NA	ADC-30
GCA_03628 7255.1_AS M3628725v 1_genomic	CP142892.1	South Korea	Korea, Republic of	2020-01-16	2020.0	Missing	Missing	2	IC2	2	3	NA	NA	ADC-30
GCA_04527 8655.1_AS M4527865v 1_genomic	CP137073.1	South Korea: Republic of	Korea, Republic of	2018-05-05T22:19Z	2018.3	Seoul National University Bundang Hospital Intensive Care Unit	Hospital_envirome nt	2	IC2	2	1	NA	NA	ADC-30
GCA_04568 9865.1_AS M4568986v 1_genomic	CP142801.1	South Korea	Korea, Republic of	2020-10-09	2020.8	human	human	2	IC2	2	5	NA	NA	ADC-30
GCA_04528 9465.1_AS M4528946v 1_genomic	CP146766.1	South Korea	Korea, Republic of	2020-12-02	2020.9	Missing	Missing	2	IC2	2	2	OXA-23	NA	ADC-30

GCA_024172225.1_ASM2417222v1_genomic	CP100305.1	South Korea	Korea, Republic of	2018-01-25	2018.1	Missing	Missing	2	IC2	2	2	NA	NA	ADC-30
GCA_023361915.1_ASM2336191v1_genomic	CP096727.1	China:Guangdong	China	2010-12-03	2010.9	sputum	Respiratory_tract	2	IC2	2	1	NA	NA	ADC-30
GCA_023361015.1_ASM2336101v1_genomic	CP096684.1	China:Guangdong	China	2016-07-18	2016.5	sputum	Respiratory_tract	2	IC2	2	1	NA	NA	ADC-30
GCA_043950125.1_ASM4395012v1_genomic	CP171791.1	China: Guangzhou	China	2024	2024	bile	Digestive_system	2	IC2	2	3	OXA-23	NA	ADC-30
GCA_023361155.1_ASM2336115v1_genomic	CP096698.1	China:Guangdong	China	2012-08-01	2012.6	bronchial	Respiratory_tract	2	IC2	2	1	NA	NA	ADC-30
GCA_023361175.1_ASM2336117v1_genomic	CP096700.1	China:Guangdong	China	2012-04-10	2012.3	blood	Blood	2	IC2	2	1	NA	NA	ADC-30
GCA_043042175.1_ASM4304217v1_genomic	CP134550.1	China: Hunan	China	2010	2010	sputum	Respiratory_tract	2	IC2	2	1	OXA-23	NA	ADC-30
GCA_003029495.1_ASM302949v1_genomic	CP027123.1	Missing	Missing	Missing	Missing	Missing	Missing	2	IC2	1	3	mphE # msrE # OXA-23	NA	ADC-212
GCA_002947415.1_ASM294741v1_genomic	CP026707.1	Missing	Missing	Missing	Missing	Missing	Missing	2	IC2	1	3	mphE # msrE # OXA-23	NA	ADC-212
GCA_000018445.1_ASM1844v1_genomic	CP000863.1	Missing	Missing	Missing	Missing	Missing	Missing	2	IC2	1	2	OXA-58 # OXA-58	NA	ADC-175

GCA_005519135.2_ASM551913v2_genomic	CP031380.1	Italy: Rome	Italy	2005	2005	cerebrospinal fluid	CNS	2	IC2	1	2	OXA-58 # APH(3')-VIa	NA	ADC-175
GCA_024732425.1_ASM2473242v1_genomic	CP031383.1	Greece: Athens	Greece	1997	1997	Missing	Missing	45	IC2	1	1	NA	NA	ADC-185
GCA_007221455.1_ASM722145v1_genomic	CP032055.1	Netherlands:Rotterdam	Netherlands	1982	1982	Missing	Missing	2	IC2	1	0	NA	NA	ADC-25
GCA_033189995.1_ASM3318999v1_genomic	CP136171.1	France: Marcy l'etoile	France	2021-12-07	2021.9	oral swab	Respiratory_tract	2	IC2	1	0	NA	NA	ADC-25
GCA_033187975.1_ASM3318797v1_genomic	CP136166.1	France: Marcy l'etoile	France	2021-12-07	2021.9	oral swab	Respiratory_tract	2	IC2	1	0	NA	NA	ADC-25
GCA_033189435.1_ASM3318943v1_genomic	CP136168.1	France: Marcy l'etoile	France	2021-12-07	2021.9	oral swab	Respiratory_tract	2	IC2	1	0	NA	NA	ADC-25
GCA_033190675.1_ASM3319067v1_genomic	CP136179.1	France: Marcy l'etoile	France	2021-05-26	2021.4	oral swab	Respiratory_tract	2	IC2	1	0	NA	NA	ADC-25
GCA_033190815.1_ASM3319081v1_genomic	CP136180.1	France: Marcy l'etoile	France	2021-05-26	2021.4	oral swab	Respiratory_tract	2	IC2	1	0	NA	NA	ADC-25
GCA_033191435.1_ASM3319143v1_genomic	CP136182.1	France: Marcy l'etoile	France	2021-05-18	2021.4	oral swab	Respiratory_tract	2	IC2	1	0	NA	NA	ADC-25
GCA_033191195.1_ASM3319119v1_genomic	CP136181.1	France: Marcy l'etoile	France	2021-05-18	2021.4	oral swab	Respiratory_tract	2	IC2	1	0	NA	NA	ADC-25

GCA_033187155.1_ASM3318715v1_genomic	CP136165.1	France: Marcy l'etoile	France	2021-12-09	2021.9	oral swab	Respiratory_tract	2	IC2	1	0	NA	NA	ADC-25
GCA_033188675.1_ASM3318867v1_genomic	CP136167.1	France: Marcy l'etoile	France	2021-12-07	2021.9	oral swab	Respiratory_tract	2	IC2	1	0	NA	NA	ADC-25
GCA_003181015.1_ASM318101v1_genomic	CP029569.1	Spain	Spain	Missing	Missing	Missing	Missing	2	IC2	1	4	APH(3')-VIa # OXA-24	NA	ADC-1
GCA_002753915.1_ASM275391v1_genomic	CP024576.1	Pakistan: Sargodha district of the Punjab province	Pakistan	2012	2012	sheep brocho-alveolar lavage	Sheep	2	IC2	1	2	OXA-23 # APH(3')-VIa	NA	ADC-25
GCA_022459875.1_ASM2245987v1_genomic	CP091370.1	Belgium	Belgium	Missing	Missing	Missing	Missing	2	IC2	1	0	NA	NA	ADC-25
GCA_023221535.1_ASM2322153v1_genomic	CP096575.1	Australia: Brisbane	Australia	1999	1999	clinical	Clinical_sample	2	IC2	1	0	NA	NA	ADC-25
GCA_000188215.1_ASM18821v1_genomic	CP001921.1	Missing	Missing	Missing	Missing	Missing	Missing	2	IC2	1	2	NA	NA	ADC-31
GCA_000498375.2_ASM49837v2_genomic	CP017152.1	Missing	Missing	Missing	Missing	Missing	Missing	2	IC2	1	0	NA	NA	ADC-31
GCA_000828795.1_ASM82879v1_genomic	AP013357.1	Missing	Missing	Missing	Missing	Missing	Missing	2	IC2	2	0	NA	NA	ADC-30

GCA_035754475.1_ASM3575447v1_genomic	CP142102.1	South Korea	Korea, Republic of	2020-01-04	2020.0	Missing	Missing	2	IC2	3	0	NA	NA	ADC-73
GCA_001806345.1_ASM180634v1_genomic	CP017642.1	South Korea:Daejeon	Korea, Republic of	2015-03-02	2015.2	Bronchial	Respiratory_tract	2	IC2	3	1	NA	NA	ADC-73
GCA_024126295.1_ASM2412629v1_genomic	CP099790.1	South Korea: Gyeonggi-do	Dominican Republic	2013-01-01	2013.0	urine	Urinary_system	2	IC2	3	2	NA	NA	ADC-73
GCA_033181005.1_ASM3318100v1_genomic	CP137059.1	South Korea: Republic of	Korea, Republic of	2016-09-02T00:20Z	2016.7	Seoul National University Bundang Hospital Intensive Care Unit	Hospital_environment	2	IC2	3	1	NA	NA	ADC-30
GCA_001806385.1_ASM180638v1_genomic	CP017646.1	South Korea:Daejeon	Korea, Republic of	2015-04-17	2015.3	sputum	Respiratory_tract	2	IC2	3	1	NA	NA	ADC-73
GCA_022459595.1_ASM2245959v1_genomic	CP091368.1	Belgium	Belgium	Missing	Missing	Missing	Missing	2	IC2	3	0	NA	NA	ADC-73
GCA_045278675.1_ASM4527867v1_genomic	CP137061.1	South Korea: Republic of	Korea, Republic of	2016-02-06T18:27Z	2016.1	Seoul National University Bundang Hospital Intensive Care Unit	Hospital_environment	2	IC2	3	1	NA	NA	ADC-73
GCA_027943685.1_ASM2794368v1_genomic	AP025531.1	Japan:Osaka	Japan	2016-08-20	2016.6	Missing	Missing	2	IC2	3	3	NA	NA	ADC-73
GCA_027943705.1_AS	AP025535.1	Japan:Osaka	Japan	2016-08-20	2016.6	Missing	Missing	2	IC2	3	3	OXA-23	NA	ADC-73

M2794370v 1_genomic														
GCA_029713905.1_ASM2971390v1_genomic	CP121579.1	Saudi Arabia:Riyadh	Saudi Arabia	2019	2019	Missing	Missing	1579	IC2	3	1	NA	NA	ADC-73
GCA_029713845.1_ASM2971384v1_genomic	CP121560.1	Saudi Arabia:Riyadh	Saudi Arabia	2019	2019	Missing	Missing	1579	IC2	3	2	NA	NA	ADC-73
GCA_043006125.1_ASM4300612v1_genomic	CP160307.1	Thailand: northern region	Thailand	2016/2017	2016	sputum	Respiratory_tract	2	IC2	3	0	NA	NA	ADC-73
GCA_040957075.1_ASM4095707v1_genomic	CP162566.1	Thailand: northern region	Thailand	2016/2017	2016	sputum	Respiratory_tract	2	IC2	3	1	NA	NA	ADC-73
GCA_029713765.1_ASM2971376v1_genomic	CP121557.1	Saudi Arabia:Riyadh	Saudi Arabia	2018	2018	Missing	Missing	1579	IC2	3	2	NA	NA	ADC-73
GCA_040267665.1_ASM4026766v1_genomic	CP158366.1	China: Shenzhen	China	2022-09-09	2022.7	Sputums	Respiratory_tract	2	IC2	3	0	NA	NA	ADC-73
GCA_040529205.1_ASM4052920v1_genomic	CP159516.1	China: Shenzhen	China	2022-09-14	2022.7	Sputums	Respiratory_tract	2	IC2	3	0	NA	NA	ADC-73
GCA_040529215.1_ASM4052921v1_genomic	CP159517.1	China: Shenzhen	China	2022-09-15	2022.7	Sputums	Respiratory_tract	2	IC2	3	0	NA	NA	ADC-73
GCA_040529225.1_ASM4052922v1_genomic	CP159518.1	China: Shenzhen	China	2022-10-07	2022.8	Sputums	Respiratory_tract	2	IC2	3	0	NA	NA	ADC-73
GCA_040529285.1_AS	CP159524.1	China: Shenzhen	China	2022-09-03	2022.7	Sputums	Respiratory_tract	2	IC2	3	0	NA	NA	ADC-73

M4052928v 1_genomic														
GCA_04052 9305.1_AS M4052930v 1_genomic	CP159526.1	China: Shenzhen	China	2022-09-03	2022.7	Sputums	Respiratory_tract	2	IC2	3	0	NA	NA	ADC-73
GCA_04053 6965.1_AS M4053696v 1_genomic	CP159626.1	China: Shenzhen	China	2022-09-03	2022.7	Sputums	Respiratory_tract	2	IC2	3	0	NA	NA	ADC-73
GCA_04052 9295.1_AS M4052929v 1_genomic	CP159525.1	China: Shenzhen	China	2022-09-03	2022.7	Sputums	Respiratory_tract	2	IC2	3	0	NA	NA	ADC-73
GCA_04052 9325.1_AS M4052932v 1_genomic	CP159528.1	China: Shenzhen	China	2022-08-30	2022.7	Sputums	Respiratory_tract	2	IC2	3	0	NA	NA	ADC-73
GCA_02971 4085.1_AS M2971408v 1_genomic	CP121629.1	Saudi Arabia:Jed dah	Saudi Arabia	2019	2019	Missing	Missing	2	IC2	3	2	NA	NA	ADC-73
GCA_02971 4025.1_AS M2971402v 1_genomic	CP121601.1	Saudi Arabia:Jed dah	Saudi Arabia	2018	2018	Missing	Missing	2	IC2	3	2	APH(3')-VIa # APH(3')-VIa	NA	ADC-73
GCA_02246 7805.1_AS M2246780v 1_genomic	CP091342.1	Belgium	Belgium	Missing	Missing	Missing	Missing	2	IC2	3	0	NA	NA	ADC-73
GCA_02971 4005.1_AS M2971400v 1_genomic	CP121598.1	Saudi Arabia:Jed dah	Saudi Arabia	2018	2018	Missing	Missing	2	IC2	3	2	APH(3')-VIa	NA	ADC-73
GCA_02971 4165.1_AS M2971416v 1_genomic	CP121588.1	Saudi Arabia:Me dina	Saudi Arabia	2019	2019	Missing	Missing	2	IC2	3	2	NA	NA	ADC-73
GCA_01860 4365.1_AS	CP071763.1	Lebanon	Lebanon	2017	2017	blood	Blood	2	IC2	3	2	NA	NA	ADC-73

M1860436v 1_genomic														
GCA_02246 8195.1_AS M2246819v 1_genomic	CP091346.1	Belgium	Belgium	Missing	Missing	Missing	Missing	2	IC2	3	0	NA	NA	ADC-73
GCA_01850 2545.1_AS M1850254v 1_genomic	CP051262.1	Turkey	Türkiye	2017-07-27	2017.6	Missing	Missing	2	IC2	3	0	NA	NA	ADC-73
GCA_02967 4685.1_AS M2967468v 1_genomic	CP121345.1	Poland: Warsaw	Poland	2012	2012	bronchial secretion	Respiratory_tract	2	IC2	3	1	APH(3')-VIb	NA	ADC-73
GCA_02474 9685.1_AS M2474968v 1_genomic	CP087348.1	Germany	Germany	Missing	Missing	Missing	Missing	2	IC2	3	2	APH(3')-VIa	NA	ADC-73
GCA_02474 9565.1_AS M2474956v 1_genomic	CP087357.1	Germany	Germany	Missing	Missing	Missing	Missing	2	IC2	3	4	APH(3')-VIb	NA	ADC-73
GCA_04052 9315.1_AS M4052931v 1_genomic	CP159527.1	China: Shenzhen	China	2022-09-03	2022.7	Sputums	Respiratory_tract	2	IC2	3	0	NA	NA	ADC-73
GCA_04052 9265.1_AS M4052926v 1_genomic	CP159522.1	China: Shenzhen	China	2022-09-03	2022.7	Sputums	Respiratory_tract	2	IC2	3	0	NA	NA	ADC-73
GCA_04052 9235.1_AS M4052923v 1_genomic	CP159519.1	China: Shenzhen	China	2022-09-03	2022.7	Sputums	Respiratory_tract	2	IC2	3	0	NA	NA	ADC-73
GCA_04053 6955.1_AS M4053695v 1_genomic	CP159625.1	China: Shenzhen	China	2022-09-30	2022.7	Sputums	Respiratory_tract	2	IC2	3	0	NA	NA	ADC-73
GCA_04052 9275.1_AS	CP159523.1	China: Shenzhen	China	2022-09-03	2022.7	Sputums	Respiratory_tract	2	IC2	3	0	NA	NA	ADC-73

M4052927v 1_genomic														
GCA_040529245.1_ASM4052924v1_genomic	CP159520.1	China: Shenzhen	China	2022-09-03	2022.7	Sputums	Respiratory_tract	2	IC2	3	0	NA	NA	ADC-73
GCA_040529255.1_ASM4052925v1_genomic	CP159521.1	China: Shenzhen	China	2022-09-03	2022.7	Sputums	Respiratory_tract	2	IC2	3	0	NA	NA	ADC-73
GCA_040536945.1_ASM4053694v1_genomic	CP159624.1	China: Shenzhen	China	2022-09-25	2022.7	Sputums	Respiratory_tract	2	IC2	3	0	NA	NA	ADC-73
GCA_037215885.1_ASM3721588v1_genomic	CP147665.1	China:Luzhou	China	2021	2021	Missing	Missing	2716	IC2	3	2	NA	NA	ADC-73
GCA_017096365.2_ASM1709636v2_genomic	CP059359.1	China: Tianjin	China	2018-02-04	2018.1	blood	Blood	2	IC2	3	1	OXA-23	NA	ADC-73
GCA_022429585.1_ASM2242958v1_genomic	CP092485.1	China: Zhuhai	China	2021-10-12	2021.8	sputum	Respiratory_tract	2	IC2	3	1	NA	NA	ADC-73
GCA_022469075.1_ASM2246907v1_genomic	CP091357.1	Belgium	Belgium	Missing	Missing	Missing	Missing	2	IC2	3	0	NA	NA	ADC-73
GCA_025449195.1_ASM2544919v1_genomic	CP104912.1	Missing	Missing	2017-03	2017.2	Missing	Missing	2	IC2	3	1	NA	NA	ADC-73
GCA_025449155.1_ASM2544915v1_genomic	CP104907.1	Missing	Missing	2017-03	2017.2	Missing	Missing	2	IC2	3	1	NA	NA	ADC-73
GCA_020520365.1_AS	CP084727.1	Hong Kong	Hong Kong	2020-07-07	2020.5	hospital	Hospital_enviroment	2	IC2	3	2	NA	NA	ADC-73

M2052036v 1_genomic														
GCA_02052 0385.1_AS M2052038v 1_genomic	CP084724.1	Hong Kong	Hong Kong	2020-09-26	2020.7	hospital	Hospital_enviroment	2	IC2	3	2	NA	NA	ADC-73
GCA_02052 0405.1_AS M2052040v 1_genomic	CP084721.1	Hong Kong	Hong Kong	2020-09-22	2020.7	hospital	Hospital_enviroment	2	IC2	3	2	NA	NA	ADC-73
GCA_02052 0325.1_AS M2052032v 1_genomic	CP084733.1	Hong Kong	Hong Kong	2020-08-30	2020.7	hospital	Hospital_enviroment	2	IC2	3	2	NA	NA	ADC-73
GCA_02052 0345.1_AS M2052034v 1_genomic	CP084730.1	Hong Kong	Hong Kong	2020-05-09	2020.4	hospital	Hospital_enviroment	2	IC2	3	2	NA	NA	ADC-73
GCA_01709 6345.2_AS M1709634v 2_genomic	CP059349.1	China: Tianjin	China	2014-04-01	2014.2	blood	Blood	2	IC2	3	1	OXA-23	NA	ADC-73
GCA_01709 6325.2_AS M1709632v 2_genomic	CP059350.1	China: Beijing	China	2018-04-06	2018.3	bronchial	Respiratory_tract	2	IC2	3	1	OXA-23	NA	ADC-73
GCA_01709 6405.2_AS M1709640v 2_genomic	CP059351.1	China: Beijing	China	2018-03-08	2018.2	sputum	Respiratory_tract	2	IC2	3	1	OXA-23	NA	ADC-73
GCA_02971 4145.1_AS M2971414v 1_genomic	CP121632.1	Saudi Arabia:Al-Ahsa	Saudi Arabia	2018	2018	Missing	Missing	2	IC2	3	1	NA	NA	ADC-73
GCA_02971 3925.1_AS M2971392v 1_genomic	CP121586.1	Saudi Arabia:Riyadh	Saudi Arabia	2018	2018	Missing	Missing	2	IC2	3	1	NA	NA	ADC-73

GCA_029713745.1_ASM2971374v1_genomic	CP121577.1	Saudi Arabia:Riyadh	Saudi Arabia	2019	2019	Missing	Missing	2	IC2	3	1	NA	NA	ADC-73
GCA_043571745.1_T3_genomic	CP171394.1	Turkey: Kayseri	Türkiye	2023-08-15	2023.6	wound	Skin	2	IC2	3	1	APH(3')-VIa	NA	ADC-73
GCA_040536855.1_ASM4053685v1_genomic	CP159579.1	China:Nanchang	China	2022-02	2022.1	Missing	Missing	2	IC2	3	2	NA	NA	ADC-73
GCA_008807055.1_ASM880705v1_genomic	CP032743.1	China: Shanghai	China	2015-06-04	2015.4	cerebral-spinal fluid	CNS	2	IC2	3	2	NA	NA	ADC-73
GCA_025854315.1_ASM2585431v1_genomic	CP107728.1	China:Xi'an	China	2018-10	2018.8	sputm	Respiratory_tract	2	IC2	3	0	NA	NA	ADC-73
GCA_003288775.1_ASM328877v1_genomic	CP030083.1	China: Beijing	China	2016-08-30	2016.7	Missing	Missing	2	IC2	3	1	NA	NA	ADC-73
GCA_003264295.1_ASM326429v1_genomic	CP019217.1	China:Zhejiang	China	2014	2014	sputum	Respiratory_tract	2	IC2	3	1	NA	NA	ADC-73
GCA_003264255.1_ASM326425v1_genomic	CP021321.1	China:Zhejiang	China	2017	2017	Missing	Missing	2	IC2	3	1	NA	NA	ADC-73
GCA_038098625.1_ASM3809862v1_genomic	CP151169.1	China	China	2018-04-28	2018.3	sputum	Respiratory_tract	2	IC2	3	0	NA	NA	ADC-73
GCA_007647215.1_ASM764721v1_genomic	CP019114.1	China:Chongqing	China	2016-07-25	2016.6	Missing	Missing	2	IC2	3	1	NA	NA	ADC-73

GCA_039518815.2_ASM3951881v2_genomic	CP155724.1	USA	United States	2024-02-23	2024.1	Wound/Abscess	Skin	2	IC2	3	1	NA	NA	ADC-73
GCF_039518815.1_ASM3951881v2_genomic	NZ_CP155724.1	USA	United States	2024-02-23	2024.1	Wound/Abscess	Skin	2	IC2	3	1	NA	NA	ADC-73
GCA_043647435.2_ASM4364743v2_genomic	CP180044.1	USA	United States	2024-09-22	2024.7	Wound/Abscess	Skin	2	IC2	3	1	NA	NA	ADC-73
GCA_043647375.2_ASM4364737v2_genomic	CP180094.1	USA	United States	2024-09-05	2024.7	Wound/Abscess	Skin	2	IC2	3	1	NA	NA	ADC-73
GCA_028622775.1_ASM2862277v1_genomic	CP117764.1	USA	United States	2022-10-10	2022.8	Wound/Abscess	Skin	2	IC2	3	1	NA	NA	ADC-73
GCA_038418265.2_ASM3841826v2_genomic	CP157255.1	USA	United States	2024-03-13	2024.2	Wound/Abscess	Skin	2	IC2	3	1	NA	NA	ADC-73
GCF_038418265.1_ASM3841826v2_genomic	NZ_CP157255.1	USA	United States	2024-03-13	2024.2	Wound/Abscess	Skin	2	IC2	3	1	NA	NA	ADC-73
GCF_038069235.1_ASM3806923v2_genomic	NZ_CP157252.1	USA	United States	2024-02-10	2024.1	Missing	Missing	2	IC2	3	2	NA	NA	ADC-73
GCA_027992645.4_ASM2799264v4_genomic	CP124678.1	USA	United States	2022-11-21	2022.9	Missing	Missing	2	IC2	3	1	NA	NA	ADC-73
GCF_027992645.1_ASM2799264v4_genomic	NZ_CP124678.1	USA	United States	2022-11-21	2022.9	Missing	Missing	2	IC2	3	1	NA	NA	ADC-73

GCA_043647275.2_ASM4364727v2_genomic	CP180098.1	USA	United States	2024-09-12	2024.7	blood	Blood	2	IC2	3	2	sul2	NA	ADC-73
GCA_044537465.2_ASM4453746v2_genomic	CP180038.1	USA	United States	2024-10-01	2024.8	Wound/Abscess	Skin	2	IC2	3	1	NA	NA	ADC-73
GCA_042709435.2_ASM4270943v2_genomic	CP180119.1	USA	United States	2024-09-04	2024.7	urine	Urinary_system	2	IC2	3	1	NA	NA	ADC-73
GCA_038069235.2_ASM3806923v2_genomic	CP157252.1	USA	United States	2024-02-10	2024.1	Missing	Missing	2	IC2	3	2	NA	NA	ADC-73
GCA_044537425.2_ASM4453742v2_genomic	CP180042.1	USA	United States	2024-10-03	2024.8	Missing	Missing	2	IC2	3	1	NA	NA	ADC-73
GCA_042700245.2_2024CK-01503_genomic	CP174430.1	USA	United States	2024-08-25	2024.7	sputum	Respiratory_tract	2	IC2	3	1	NA	NA	ADC-73
GCA_043647295.2_ASM4364729v2_genomic	CP180096.1	USA	United States	2024-09-22	2024.7	urine	Urinary_system	2	IC2	3	1	NA	NA	ADC-73
GCA_028622915.1_ASM2862291v1_genomic	CP117759.1	USA	United States	2022-11-14	2022.9	Missing	Missing	2	IC2	3	1	NA	NA	ADC-73
GCA_030704325.1_ASM3070432v1_genomic	CP131944.1	USA	United States	2023-05-16	2023.4	urine	Urinary_system	2	IC2	3	1	NA	NA	ADC-73
GCA_043647495.2_AS	CP180111.1	USA	United States	2024-09-23	2024.7	urine	Urinary_system	2	IC2	3	1	NA	NA	ADC-73

M4364749v2_genomic														
GCA_042443115.2_2024CK-01459_genomic	CP174435.1	USA	United States	2024-09-03	2024.7	Tissue	Skin	2	IC2	3	0	NA	NA	ADC-73
GCA_042209595.2_ASM4220959v2_genomic	CP174047.1	USA	United States	2024-08-13	2024.6	Missing	Missing	2	IC2	3	2	NA	NA	ADC-268
GCA_042945475.2_2024CK-01504_genomic	CP174432.1	USA	United States	2024-09-07	2024.7	Tracheal Aspirate/Wash	Respiratory_tract	2	IC2	3	2	NA	NA	ADC-268
GCA_043647555.2_ASM4364755v2_genomic	CP180105.1	USA	United States	2024-09-30	2024.7	Wound/Abscess	Skin	2	IC2	3	2	NA	NA	ADC-268
GCA_043647585.2_ASM4364758v2_genomic	CP180108.1	USA	United States	2024-09-30	2024.7	skin	Skin	2	IC2	3	2	NA	NA	ADC-268
GCA_043647545.2_ASM4364754v2_genomic	CP180103.1	USA	United States	2024-09-30	2024.7	skin	Skin	2	IC2	3	1	NA	NA	ADC-268
GCA_044827905.2_ASM4482790v2_genomic	CP180031.1	USA	United States	2024-10-21	2024.8	Wound/Abscess	Skin	2	IC2	3	2	NA	NA	ADC-268
GCA_042809665.2_ASM4280966v2_genomic	CP180115.1	USA	United States	2024-09-09	2024.7	sputum	Respiratory_tract	2	IC2	3	2	NA	NA	ADC-268
GCF_038069255.1_ASM3806925v2_genomic	NZ_CP157249.1	USA	United States	2024-02-20	2024.1	sputum	Respiratory_tract	2	IC2	3	2	NA	NA	ADC-73

GCA_038069255.2_ASM3806925v2_genomic	CP157249.1	USA	United States	2024-02-20	2024.1	sputum	Respiratory_tract	2	IC2	3	2	NA	NA	ADC-73
GCA_043647235.2_ASM4364723v2_genomic	CP180113.1	USA	United States	2024-09-19	2024.7	sputum	Respiratory_tract	2	IC2	3	1	NA	NA	ADC-73
GCA_022459055.1_ASM2245905v1_genomic	CP091359.1	Belgium	Belgium	Missing	Missing	Missing	Missing	2	IC2	3	0	NA	NA	ADC-73
GCA_039701065.1_ASM3970106v1_genomic	CP155637.1	China:Jinan	China	2024-03	2024.2	sputum	Respiratory_tract	2	IC2	3	1	NA	NA	ADC-73
GCA_025916215.1_ASM2591621v1_genomic	CP109836.1	China: Hangzhou	China	2022-10-01	2022.8	cerebrospinal fluid	CNS	2	IC2	3	1	NA	NA	ADC-268
GCA_025449175.1_ASM2544917v1_genomic	CP104908.1	Missing	Missing	2017-03	2017.2	Missing	Missing	2	IC2	3	2	NA	NA	ADC-73
GCA_025402975.1_ASM2540297v1_genomic	CP104786.1	Missing	Missing	2017-03	2017.2	Missing	Missing	2	IC2	3	2	NA	NA	ADC-73
GCA_023361115.1_ASM2336111v1_genomic	CP096692.1	China:Beijing	China	2018-12-13	2018.9	sputum	Respiratory_tract	2	IC2	3	0	NA	NA	ADC-73
GCA_019356075.1_ASM1935607v1_genomic	CP079942.1	China: Beijing	China	2018-12-13	2018.9	sputum	Respiratory_tract	2	IC2	3	0	NA	NA	ADC-73
GCA_028548795.2_ASM2854879v2_genomic	CP166770.1	China: Anhui	China	2022-03	2022.2	cerebrospinal fluid	CNS	2	IC2	3	1	NA	NA	ADC-73

GCA_023517255.1_ASM2351725v1_genomic	CP077837.1	China: Hangzhou, Sir Run Run Shaw Hospital ICU	China	2019-09-03	2019.7	Locker_P26_BU28	Hospital_enviroment	2	IC2	3	2	NA	NA	ADC-73
GCA_029767905.3_ASM2976790v3_genomic	CP130921.1	USA	United States	2023-03-12	2023.2	sputum	Respiratory_tract	2	IC2	3	2	NA	NA	ADC-73
GCF_029767905.1_ASM2976790v3_genomic	NZ_CP130921.1	USA	United States	2023-03-12	2023.2	sputum	Respiratory_tract	2	IC2	3	2	NA	NA	ADC-73
GCA_023615705.2_ASM2361570v2_genomic	CP115629.1	USA	United States	2022-05-12	2022.4	sputum	Respiratory_tract	2	IC2	3	2	NA	NA	ADC-73
GCF_023615705.1_ASM2361570v2_genomic	NZ_CP115629.1	USA	United States	2022	2022	Respiratory_tract	Respiratory_tract	2	IC2	3	2	NA	NA	ADC-73
GCA_030718975.1_ASM3071897v1_genomic	CP132214.1	USA	United States	2022-01-20	2022.1	Wound/Abscess	Skin	2	IC2	3	1	NA	NA	ADC-268
GCA_028622715.1_ASM2862271v1_genomic	CP117762.1	USA	United States	2022-12-10	2022.9	blood	Blood	2	IC2	3	2	NA	NA	ADC-268
GCA_045289435.1_ASM4528943v1_genomic	CP143269.1	South Korea	Korea, Republic of	2020-01-22	2020.1	Missing	Missing	2	IC2	3	2	NA	NA	NA
GCA_023361975.1_ASM2336197v1_genomic	CP096734.1	China:Guangdong	China	2016-07-13	2016.5	blood	Blood	2	IC2	3	0	NA	NA	ADC-73
GCA_028622695.1_AS	CP117728.1	USA	United States	2022-12-01	2022.9	urine	Urinary_system	2	IC2	3	1	NA	NA	ADC-73

M2862269v 1_genomic														
GCA_02351 7215.1_AS M2351721v 1_genomic	CP077832.1	China: Hangzhou , Sir Run Run Shaw Hospital ICU	China	2019-09-03	2019.7	Nasojejunal tube_P50_BU 7	Digestive_system	2	IC2	3	2	NA	NA	ADC-73
GCA_04215 0455.1_Rp6 68_genomic	CP169857.1	France: Lens	France	2018-12-28	2018.9	Respiratory/B AL	Respiratory_tract	2	IC2	3	1	NA	NA	ADC-73
GCA_02351 7735.1_AS M2351773v 1_genomic	CP077846.1	China: Hangzhou , Sir Run Run Shaw Hospital ICU	China	2019-08-20	2019.6	Nasogastric tube_P44_BU 19	Digestive_system	2	IC2	3	2	NA	NA	ADC-73
GCA_04534 8215.1_AS M4534821v 1_genomic	CP155446.1	China:Hen an	China	2023-03-18	2023.2	sputum	Respiratory_tract	2	IC2	3	0	NA	NA	ADC-73
GCA_02245 9155.1_AS M2245915v 1_genomic	CP091364.1	Belgium	Belgium	Missing	Missing	Missing	Missing	2	IC2	3	0	NA	NA	ADC-73
GCA_02981 4405.1_AS M2981440v 1_genomic	CP122360.1	Russia:Mo scow	Russian Federatio n	2021-07-13	2021.5	tracheal aspirate	Respiratory_tract	2	IC2	3	2	OXA-23 # APH(3')-VIa	NA	ADC-73
GCA_02971 3945.1_AS M2971394v 1_genomic	CP121625.1	Saudi Arabia:Jed dah	Saudi Arabia	2019	2019	Missing	Missing	2	IC2	3	3	APH(3')-VIa	NA	ADC-73
GCA_05003 0675.1_AS M5003067v 1_genomic	CP179692.1	Paraguay: Itaugua	Paraguay	2024-02-02	2024.1	tracheal secretion	Respiratory_tract	2	IC2	3	1	NA	NA	ADC-73
GCA_04806 7285.1_AS	CP179705.1	Paraguay: Itaugua	Paraguay	2024-01-14	2024.0	tracheal secretion	Respiratory_tract	2	IC2	3	1	NA	NA	ADC-73

M4806728v1_genomic														
GCA_048069565.1_ASM4806956v1_genomic	CP179709.1	Paraguay: Itaugua	Paraguay	2024-02-06	2024.1	tracheal secretion	Respiratory_tract	2	IC2	3	1	NA	NA	ADC-73
GCA_048067295.1_ASM4806729v1_genomic	CP179707.1	Paraguay: Itaugua	Paraguay	2024-01-03	2024.0	tracheal secretion	Respiratory_tract	2	IC2	3	1	NA	NA	ADC-73
GCA_042165515.2_2024CK-01391_genomic	CP174440.1	USA	United States	2024-09-03	2024.7	skin	Skin	2	IC2	3	1	NA	NA	ADC-73
GCA_045689335.1_ASM4568933v1_genomic	CP137140.1	South Korea	Korea, Republic of	2021-07-16	2021.5	Missing	Missing	2	IC2	3	1	NA	NA	ADC-73
GCA_012935185.1_ASM1293518v1_genomic	CP051474.1	India	India	2019	2019	sputum	Respiratory_tract	2	IC2	3	3	NA	NA	ADC-73
GCA_012935145.1_ASM1293514v1_genomic	CP050523.1	India	India	2019	2019	blood	Blood	2	IC2	3	2	NA	NA	ADC-73
GCA_012934985.1_ASM1293498v1_genomic	CP050401.1	India	India	2019	2019	sputum	Respiratory_tract	2	IC2	3	1	NA	NA	ADC-73
GCA_012935045.1_ASM1293504v1_genomic	CP050410.1	India	India	2019	2019	Pus	Skin	2	IC2	3	1	NA	NA	ADC-73
GCA_042152975.1_Up367_genomic	CP169870.1	Malaysia	Malaysia	2013	2013	Urinary source	Urinary_system	2	IC2	3	1	NA	NA	ADC-73
GCA_000695855.3_AS	CP007535.2	Malaysia: Kuala	Malaysia	2011-07-31	2011.6	Endotracheal Secretion	Respiratory_tract	2	IC2	3	2	OXA-23	NA	ADC-73

M69585v3_genomic		Terengganu, Terengganu												
GCA_048633555.1_ASM4863355v1_genomic	CP184591.1	China:Chongqing	China	2022-08-08	2022.6	BALF	Respiratory_tract	2	IC2	3	1	OXA-23	NA	ADC-30
GCA_048636685.1_ASM4863668v1_genomic	CP184613.1	China:Chongqing	China	2023-01-04	2023.0	BALF	Respiratory_tract	2	IC2	3	1	OXA-23	NA	ADC-30
GCA_048636155.1_ASM4863615v1_genomic	CP184607.1	China:Chongqing	China	2023-02-21	2023.1	BALF	Respiratory_tract	2	IC2	3	1	NA	lpxD # lpxA	ADC-30
GCA_048632625.1_ASM4863262v1_genomic	CP184585.1	China:Chongqing	China	2022-08-17	2022.6	BALF	Respiratory_tract	2	IC2	3	1	armA # msrE # mphE # adeH # adeF # adeG # adeL # abeS	csuE # csuD # csuC # csuB # csuA # csuA/B # adeH # adeG # adeF # gspE2 # gspL # gspM	ADC-30
GCA_048635255.1_ASM4863525v1_genomic	CP184603.1	China:Chongqing	China	2023-01-13	2023.0	BALF	Respiratory_tract	2	IC2	3	1	NA	lpxD # lpxA	ADC-73
GCA_048636165.1_ASM4863616v1_genomic	CP184609.1	China:Chongqing	China	2023-01-15	2023.0	BALF	Respiratory_tract	2	IC2	3	1	OXA-23	NA	ADC-73
GCA_048633525.1_ASM4863352v1_genomic	CP184589.1	China:Chongqing	China	2023-04-19	2023.3	BALF	Respiratory_tract	2	IC2	3	1	NA	vgrG/tssI # lpxC	ADC-73
GCA_048634365.1_ASM4863436v1_genomic	CP184595.1	China:Chongqing	China	2023-04-06	2023.3	BALF	Respiratory_tract	2	IC2	3	1	NA	NA	ADC-73

GCA_048635245.1_ASM4863524v1_genomic	CP184601.1	China:Chongqing	China	2022-10-24	2022.8	BALF	Respiratory_tract	2	IC2	3	1	NA	NA	ADC-73
GCA_048634375.1_ASM4863437v1_genomic	CP184593.1	China:Chongqing	China	2023-04-10	2023.3	BALF	Respiratory_tract	2	IC2	3	1	armA # msrE # mphE # adeH # adeF # adeG # adeL # abeS	csuE # csuD # csuC # csuB # csuA # csuA/B # adeH # adeG # adeF # gspE2 # gspL # gspM	ADC-73
GCA_048634385.1_ASM4863438v1_genomic	CP184597.1	China:Chongqing	China	2023-04-11	2023.3	BALF	Respiratory_tract	2	IC2	3	1	NA	NA	ADC-73
GCA_048635235.1_ASM4863523v1_genomic	CP184599.1	China:Chongqing	China	2023-01-04	2023.0	BALF	Respiratory_tract	2	IC2	3	1	NA	lpxC # vgrG/tssI	ADC-73
GCA_048632605.1_ASM4863260v1_genomic	CP184583.1	China:Chongqing	China	2023-05-04	2023.3	BALF	Respiratory_tract	2	IC2	3	1	NA	NA	ADC-73
GCA_048636675.1_ASM4863667v1_genomic	CP184611.1	China:Chongqing	China	2023-03-23	2023.2	BALF	Respiratory_tract	2	IC2	3	1	OXA-23	NA	ADC-73
GCA_048636145.1_ASM4863614v1_genomic	CP184605.1	China:Chongqing	China	2023-01-18	2023.0	BALF	Respiratory_tract	2	IC2	3	1	NA	lpxC # vgrG/tssI	ADC-73
GCA_048633515.1_ASM4863351v1_genomic	CP184587.1	China:Chongqing	China	2023-01-13	2023.0	BALF	Respiratory_tract	2	IC2	3	1	OXA-23	NA	ADC-73
GCA_022916555.2_ASM2291655v2_genomic	CP115626.1	USA	United States	2022-03-03	2022.2	Missing	Missing	2	IC2	3	2	NA	NA	ADC-73

GCF_022916555.1_ASM2291655v2_genomic	NZ_CP115626.1	USA	United States	2022	2022	Human_related	Human_related	2	IC2	3	2	NA	NA	ADC-73
GCA_041292045.2_2024CK-01225_genomic	CP169605.1	USA	United States	2024-08-05	2024.6	skin	Skin	2	IC2	3	2	OXA-24	NA	ADC-73
GCA_041292025.2_2024CK-01226_genomic	CP169608.1	USA	United States	2024-08-05	2024.6	skin	Skin	2	IC2	3	2	OXA-24	NA	ADC-73
GCA_041292065.2_2024CK-01224_genomic	CP169602.1	USA	United States	2024-08-05	2024.6	skin	Skin	2	IC2	3	2	OXA-24	NA	ADC-73
GCA_038418285.2_ASM3841828v2_genomic	CP157480.1	USA	United States	2024-03-31	2024.2	blood	Blood	2	IC2	3	2	NA	NA	ADC-268
GCF_038418285.1_ASM3841828v2_genomic	NZ_CP157480.1	USA	United States	2024-03-31	2024.2	blood	Blood	2	IC2	3	2	NA	NA	ADC-268
GCA_022468355.1_ASM2246835v1_genomic	CP091348.1	Belgium	Belgium	Missing	Missing	Missing	Missing	2	IC2	3	0	NA	NA	ADC-73
GCA_022467255.1_ASM2246725v1_genomic	CP091336.1	Belgium	Belgium	Missing	Missing	Missing	Missing	2	IC2	3	0	NA	NA	ADC-73
GCA_009498335.1_ASM949833v1_genomic	CP042931.1	Thailand: Chiang Mai	Thailand	2019-04	2019.2	sputum	Respiratory_tract	2	IC2	3	0	NA	NA	ADC-73

GCA_043007235.1_ASM4300723v1_genomic	CP160306.1	Thailand: northern region	Thailand	2016/2017	2016	sputum	Respiratory_tract	2	IC2	3	0	NA	NA	ADC-73
GCA_039566025.1_ASM3956602v1_genomic	CP101653.1	Thailand	Thailand	2005-12-14	2005.9	sputum	Respiratory_tract	2	IC2	3	1	NA	NA	ADC-73
GCA_012935065.1_ASM1293506v1_genomic	CP050412.1	India	India	2019	2019	sputum	Respiratory_tract	2	IC2	3	2	APH(3')-VIa	NA	ADC-73
GCA_012934965.1_ASM1293496v1_genomic	CP050400.1	India	India	2019	2019	blood	Blood	2	IC2	3	6	APH(3')-VIa # sul2	NA	ADC-73
GCA_012935165.1_ASM1293516v1_genomic	CP050526.1	India	India	2019	2019	sputum	Respiratory_tract	2	IC2	3	3	APH(3')-VIa	NA	ADC-73
GCA_012935025.1_ASM1293502v1_genomic	CP050421.1	India	India	2019	2019	sputum	Respiratory_tract	2	IC2	3	2	APH(3')-VIa	NA	ADC-73
GCA_000576535.1_ASM57653v1_genomic	CP006963.1	India: Pondicherry	India	Missing	Missing	wound infection of a hospitalized patient	Skin	NA	NA	NA	1	NA	NA	ADC-73
GCA_022468455.1_ASM2246845v1_genomic	CP091349.1	Belgium	Belgium	Missing	Missing	Missing	Missing	2	IC2	3	0	NA	NA	ADC-73
GCA_022468755.1_ASM2246875v1_genomic	CP091352.1	Belgium	Belgium	Missing	Missing	Missing	Missing	2	IC2	3	0	NA	NA	ADC-73
GCA_002009115.1_ASM200911v1_genomic	CP014291.1	Missing	Missing	2015-12-01	2015.9	Missing	Missing	2	IC2	3	2	APH(3')-VIa	NA	ADC-73

GCA_022467735.1_ASM2246773v1_genomic	CP091341.1	Belgium	Belgium	Missing	Missing	Missing	Missing	604	IC2	3	0	NA	NA	ADC-73
GCA_012934945.1_ASM1293494v1_genomic	CP050390.1	India	India	2019	2019	blood	Blood	2	IC2	3	1	NA	NA	ADC-73
GCA_029748815.1_ASM2974881v1_genomic	CP121583.1	Saudi Arabia:Riyadh	Saudi Arabia	2018	2018	Missing	Missing	2	IC2	3	2	NA	NA	ADC-73
GCA_038441205.1_ASM3844120v1_genomic	CP131889.1	Taiwan	Taiwan	2006	2006	respiratory	Respiratory_tract	2	IC2	2	3	OXA-72	NA	ADC-30
GCA_023361215.1_ASM2336121v1_genomic	CP096704.1	China:Zhejiang	China	2010-11-15	2010.9	blood	Blood	2	IC2	2	0	NA	NA	ADC-30
GCA_023361835.1_ASM2336183v1_genomic	CP096717.1	China:Zhejiang	China	2010-12-03	2010.9	sputum	Respiratory_tract	2	IC2	2	2	sul2	NA	ADC-30
GCA_023361235.1_ASM2336123v1_genomic	CP096705.1	China:Zhejiang	China	2012-02-01	2012.1	blood	Blood	2	IC2	2	1	NA	NA	ADC-30
GCA_039658505.1_ASM3965850v1_genomic	CP155443.1	China:Zhengzhou	China	2023-11-22	2023.9	sputum	Respiratory_tract	2	IC2	2	0	NA	NA	ADC-30
GCA_023517695.1_ASM2351769v1_genomic	CP077840.1	China: Hangzhou, Sir Run Run Shaw Hospital ICU	China	2019-10-29	2019.8	Rectal swab_P131_BU4	Digestive_system	2	IC2	2	2	NA	NA	ADC-30
GCA_023361075.1_AS	CP096690.1	China:Zhejiang	China	2018-05-11	2018.4	blood	Blood	2	IC2	2	1	NA	NA	ADC-30

M2336107v 1_genomic														
GCA_02155 9695.1_AS M2155969v 1_genomic	CP054416.1	China	China	2018-11	2018.8	Eye secretion	Eye	2	IC2	2	1	NA	NA	ADC-30
GCA_02336 1275.1_AS M2336127v 1_genomic	CP096710.1	China:Sha nxi	China	2012-04-25	2012.3	balf	Respiratory_tract	2	IC2	2	3	OXA-23	NA	ADC-30
GCA_02336 1035.1_AS M2336103v 1_genomic	CP096686.1	China:Xinji ang	China	2018-09-06	2018.7	blood	Blood	2	IC2	2	1	NA	NA	ADC-30
GCA_01573 2435.1_AS M1573243v 1_genomic	CP060732.1	China:Beiji ng	China	2018-04-23	2018.3	bronchial	Respiratory_tract	1555	IC2	2	0	NA	NA	ADC-30
GCA_02336 2235.1_AS M2336223v 1_genomic	CP096764.1	China:Liao ning	China	2013-03-01	2013.2	sputum	Respiratory_tract	2	IC2	2	1	NA	NA	ADC-30
GCA_02336 2195.1_AS M2336219v 1_genomic	CP096759.1	China:Liao ning	China	2013-05-09	2013.4	sputum	Respiratory_tract	2	IC2	2	2	NA	NA	ADC-30
GCA_01709 6385.2_AS M1709638v 2_genomic	CP059353.1	China: Liaoning, Shenyang	China	2016-03-18	2016.2	cerebrospinal fluid	CNS	2	IC2	2	3	OXA-893	NA	ADC-30
GCA_01709 6425.2_AS M1709642v 2_genomic	CP059352.1	China: Liaoning, Shenyang	China	2014-05-05	2014.3	blood	Blood	2	IC2	2	3	OXA-23	NA	ADC-30
GCA_02336 1995.1_AS M2336199v 1_genomic	CP096735.1	China:Beiji ng	China	2016-01-20	2016.1	blood	Blood	2	IC2	2	2	OXA-23	NA	ADC-30
GCA_02336 0975.1_AS	CP096681.1	China:Zhej iang	China	2018-10-30	2018.8	blood	Blood	2	IC2	2	0	NA	NA	ADC-30

M2336097v 1_genomic														
GCA_02351 7195.1_AS M2351719v 1_genomic	CP077830.1	China: Hangzhou , Sir Run Run Shaw Hospital ICU	China	2019-08-13	2019.6	Bed controller_P2 2_BU23	Hospital_envirome nt	2	IC2	2	1	NA	NA	ADC-30
GCA_02336 1095.1_AS M2336109v 1_genomic	CP096693.1	China:Xinji ang	China	2012-03-03	2012.2	secretion	Secretion	2	IC2	2	2	OXA-23	NA	ADC-30
GCA_05003 1815.1_AS M5003181v 1_genomic	CP188275.1	China:Nan chang	China	2023-12	2023.9	sputum	Respiratory_tract	2	IC2	2	2	NA	NA	ADC-30
GCA_02336 0995.1_AS M2336099v 1_genomic	CP096682.1	China:Beiji ng	China	2018-10-25	2018.8	tracheal aspirate	Respiratory_tract	2	IC2	2	1	NA	NA	ADC-30
GCA_02336 2155.1_AS M2336215v 1_genomic	CP096755.1	China:Liao ning	China	2014-06-16	2014.5	blood	Blood	2	IC2	2	1	NA	NA	ADC-30
GCA_02336 2135.1_AS M2336213v 1_genomic	CP096753.1	China:Zhej iang	China	2014-05-05	2014.3	blood	Blood	2	IC2	2	1	NA	NA	ADC-30
GCA_01709 8205.2_AS M1709820v 2_genomic	CP059354.1	China: Beijing	China	2014-04-07	2014.3	blood	Blood	2	IC2	2	1	OXA-23	NA	ADC-30
GCA_02336 1935.1_AS M2336193v 1_genomic	CP096729.1	China:Beiji ng	China	2010-11-03	2010.8	secretion	Secretion	2	IC2	2	1	OXA-23	NA	ADC-30
GCA_02336 2115.1_AS M2336211v 1_genomic	CP096749.1	China:Xinji ang	China	2016-03-17	2016.2	blood	Blood	2	IC2	2	3	OXA-23	NA	ADC-30

GCA_023362275.1_ASM2336227v1_genomic	CP096768.1	China:Beijing	China	2016-12-26	2016.9	blood	Blood	2	IC2	2	2	OXA-23	NA	ADC-30
GCA_021398135.1_ASM2139813v1_genomic	CP090182.1	China: Zhuhai	China	2021-10	2021.8	sputum	Respiratory_tract	2	IC2	2	1	NA	NA	ADC-30
GCA_050031495.1_ASM5003149v1_genomic	CP188278.1	China:Nanchang	China	2023-10	2023.8	sputum	Respiratory_tract	2	IC2	2	2	NA	NA	ADC-30
GCA_024730805.1_ASM2473080v1_genomic	CP102831.1	China: Zhuhai	China	2022-03	2022.2	Bronchoalveolar lavage fluid	Respiratory_tract	2	IC2	2	1	NA	NA	ADC-30
GCA_022921035.1_ASM2292103v1_genomic	CP095091.1	China:Zhuhai	China	2022-01	2022.0	sputum	Respiratory_tract	2	IC2	2	1	NA	NA	ADC-30
GCA_024800505.1_ASM2480050v1_genomic	CP103413.1	China:Zhuhai	China	2022-03	2022.2	sputum	Respiratory_tract	2	IC2	2	2	NA	NA	ADC-30
GCA_023362175.1_ASM2336217v1_genomic	CP096757.1	China:Beijing	China	2014-07-17	2014.5	blood	Blood	2	IC2	2	1	NA	NA	ADC-30
GCA_001887305.1_ASM188730v1_genomic	CP018143.1	China: Beijing	China	2014-09	2014.7	sputum	Respiratory_tract	2	IC2	2	1	OXA-23	NA	ADC-30
GCA_024134625.1_ASM2413462v1_genomic	CP099858.1	Missing	Missing	Missing	Missing	Missing	Missing	2	IC2	2	0	NA	NA	ADC-30
GCA_024134685.1_ASM2413468v1_genomic	CP099857.1	Missing	Missing	Missing	Missing	Missing	Missing	2	IC2	2	0	NA	NA	ADC-30

GCA_021725415.1_ASM2172541v1_genomic	CP091328.1	China:wenzhou	China	2018	2018	Missing	Missing	2	IC2	2	1	NA	NA	ADC-30
GCA_050320325.1_Up645_genomic	CP190387.1	USA	United States	2020	2020	respiratory	Respiratory_tract	2	IC2	2	1	NA	NA	ADC-30
GCA_023361815.1_ASM2336181v1_genomic	CP096714.1	China:Beijing	China	2011-10-05	2011.8	abdominal fluid	Abdomen	2	IC2	2	2	OXA-23	NA	ADC-30
GCA_023361195.1_ASM2336119v1_genomic	CP096702.1	China:Beijing	China	2012-07-10	2012.5	blood	Blood	2	IC2	2	1	NA	NA	ADC-30
GCA_023517715.1_ASM2351771v1_genomic	CP077843.1	China: Hangzhou, Sir Run Run Shaw Hospital ICU	China	2019-09-03	2019.7	Infusion stand_P63_BU16	Hospital_enviroment	2	IC2	2	2	NA	NA	ADC-30
GCA_023362035.1_ASM2336203v1_genomic	CP096740.1	China:Shanghai	China	2013-06-01	2013.4	blood	Blood	2	IC2	2	1	NA	NA	ADC-30
GCA_023362055.1_ASM2336205v1_genomic	CP096742.1	China:Shanghai	China	2013-03-08	2013.2	blood	Blood	2	IC2	2	2	NA	NA	ADC-30
GCA_017098225.2_ASM1709822v2_genomic	CP059356.1	China: Wuhan, Hubei	China	2018-06-14	2018.5	abdominal fluid	Abdomen	2	IC2	2	2	OXA-23	NA	ADC-30
GCA_043046865.1_ASM4304686v1_genomic	CP134564.1	China: Henan	China	2010	2010	secretion	Secretion	2	IC2	2	1	OXA-23	NA	ADC-30
GCA_000419425.1_AS	CP003849.1	Missing	Missing	Missing	Missing	Missing	Missing	2	IC2	2	3	APH(3')-Ia # AAC(6')-Ib9 #	NA	ADC-30

M41942v1_genomic												catB8 # aadA # qacEdelta1 # sul1 # armA # msrE # mphE		
GCA_038441225.1_ASM3844122v1_genomic	CP131896.1	Taiwan	Taiwan	2010	2010	respiratory	Respiratory_tract	2	IC2	2	1	OXA-72 # OXA-72	NA	ADC-115
GCA_038441255.1_ASM3844125v1_genomic	CP131903.1	Taiwan	Taiwan	2010	2010	respiratory	Respiratory_tract	2	IC2	2	2	NA	NA	ADC-30
GCA_038441215.1_ASM3844121v1_genomic	CP131893.1	Taiwan	Taiwan	2008	2008	Abs/pus/wd	Skin	2	IC2	2	2	OXA-23	NA	ADC-30
GCA_017098245.2_ASM1709824v2_genomic	CP059357.1	China: Ha'erbin, Heilongjiang	China	2018-07-05	2018.5	abdominal fluid	Abdomen	2	IC2	3	1	OXA-23	NA	ADC-73
GCA_017098265.2_ASM1709826v2_genomic	CP059358.1	China: Ha'erbin, Heilongjiang	China	2018-06-26	2018.5	pus	Skin	2	IC2	3	1	OXA-23	NA	ADC-73
GCA_014705785.1_ASM1470578v1_genomic	CP061517.1	USA:DC	United States	2007	2007	urine	Urinary_system	2	IC2	2	1	NA	NA	ADC-30
GCA_002761575.1_ASM276157v1_genomic	CP024124.1	Australia: Melbourne	Australia	2013-10-11	2013.8	wound	Skin	2	IC2	2	1	NA	NA	ADC-30
GCA_022468565.1_ASM2246856v1_genomic	CP091350.1	Belgium	Belgium	Missing	Missing	Missing	Missing	2	IC2	2	0	NA	NA	ADC-30

GCA_020906565.1_ASM2090656v1_genomic	CP086759.1	Germany	Germany	2018-06-17	2018.5	Missing	Missing	2	IC2	2	0	NA	NA	ADC-30
GCA_014705865.1_ASM1470586v1_genomic	CP061521.1	USA:DC	United States	2004	2004	sterile body site	Clinical_sample	2	IC2	2	1	NA	NA	ADC-30
GCA_014705825.1_ASM1470582v1_genomic	CP061525.1	USA:DC	United States	2009	2009	urine	Urinary_system	2	IC2	2	1	NA	NA	ADC-30
GCA_014705885.1_ASM1470588v1_genomic	CP061519.1	USA:DC	United States	2004	2004	wound	Skin	2	IC2	2	1	NA	NA	ADC-30
GCA_014705845.1_ASM1470584v1_genomic	CP061523.1	USA:DC	United States	2005	2005	blood	Blood	2	IC2	2	1	NA	NA	ADC-30
GCA_014705805.1_ASM1470580v1_genomic	CP061514.1	USA:DC	United States	2005	2005	sterile body site	Clinical_sample	2	IC2	2	2	NA	NA	ADC-30
GCA_001806485.1_ASM180648v1_genomic	CP017656.1	South Korea:Gyeonggi	Korea, Republic of	2015-09-10	2015.7	wound	Skin	2	IC2	2	1	NA	NA	ADC-115
GCA_024126255.1_ASM2412625v1_genomic	CP099784.1	South Korea: Jeollabuk-do	Dominican Republic	2013-01-01	2013.0	sputum	Respiratory_tract	2	IC2	2	1	NA	NA	ADC-30
GCA_023361855.1_ASM2336185v1_genomic	CP096720.1	China:Tianjin	China	2010-11-16	2010.9	sputum	Respiratory_tract	2	IC2	2	1	NA	NA	ADC-30
GCA_023362075.1_ASM2336207v1_genomic	CP096745.1	China:Tianjin	China	2013-06-01	2013.4	blood	Blood	2	IC2	2	1	NA	NA	ADC-30

GCA_023361135.1_ASM2336113v1_genomic	CP096696.1	China:Tianjin	China	2012-05-07	2012.3	balf	Respiratory_tract	2	IC2	2	1	NA	NA	ADC-30
GCA_023362015.1_ASM2336201v1_genomic	CP096738.1	China:Tianjin	China	2014-03-17	2014.2	blood	Blood	2	IC2	2	1	NA	NA	ADC-30
GCA_023361055.1_ASM2336105v1_genomic	CP096688.1	China:Liaoning	China	2018-11-13	2018.9	blood	Blood	2	IC2	2	1	NA	NA	ADC-30
GCA_023362095.1_ASM2336209v1_genomic	CP096747.1	China:Tianjin	China	2013-05-02	2013.3	blood	Blood	2	IC2	2	1	NA	NA	ADC-30
GCA_023361875.1_ASM2336187v1_genomic	CP096722.1	China:Shanghai	China	2010-12-14	2010.9	sputum	Respiratory_tract	2	IC2	2	1	NA	NA	ADC-30
GCA_001026965.1_ASM102696v1_genomic	CP010779.1	China:Hangzhou	China	2014-05-29	2014.4	Lower respiratory tract	Respiratory_tract	2	IC2	2	1	NA	NA	ADC-30
GCA_003264275.1_ASM326427v1_genomic	CP021326.1	China:Zhejiang	China	2017	2017	Missing	Missing	2	IC2	2	1	NA	NA	ADC-30
GCA_022759565.1_ASM2275956v1_genomic	CP094283.1	China:Hebei	China	2018	2018	ascites	Abdomen	2	IC2	2	4	NA	NA	ADC-30
GCA_028752795.1_ASM2875279v1_genomic	CP079943.1	Australia: Flinders University	Australia	2004	2004	Missing	Missing	2	IC2	2	0	NA	NA	ADC-30
GCA_028752815.1_ASM2875281v1_genomic	CP079944.1	Australia: Flinders	Australia	2004	2004	Missing	Missing	2	IC2	2	0	NA	NA	ADC-30

GCA_028751705.1_ASM2875170v1_genomic	CP079743.1	Australia: Flinders	Australia	2004	2004	Missing	Missing	2	IC2	2	1	NA	NA	ADC-30
GCA_030168285.1_ASM3016828v1_genomic	CP096894.1	USA:Georgia,Atlanta	United States	2012/2015	2014	respiratory	Respiratory_tract	2	IC2	2	7	NA	NA	ADC-30
GCA_050320315.1_Up644_genomic	CP190383.1	USA	United States	2020	2020	respiratory	Respiratory_tract	2	IC2	2	3	NA	gspE1	ADC-56
GCA_042140525.1_Hv652_genomic	CP169785.1	USA	United States	2020	2020	respiratory	Respiratory_tract	2	IC2	2	2	NA	NA	ADC-30
GCA_042149725.1_Rp654_genomic	CP169852.1	USA	United States	2020	2020	respiratory	Respiratory_tract	2	IC2	2	4	NA	NA	ADC-30
GCA_005671375.1_ASM567137v1_genomic	CP040425.1	USA:Valhalla	United States	2016	2016	Missing	Missing	2	IC2	2	2	msrE # mphE	NA	ADC-30
GCA_042144705.1_Lv643_genomic	CP169823.1	USA	United States	2020	2020	respiratory	Respiratory_tract	2	IC2	2	2	NA	NA	ADC-30
GCA_042149195.1_Rp436_genomic	CP169848.1	USA	United States	2019	2019	Respiratory/sputum	Respiratory_tract	2	IC2	2	3	APH(3')-VIa	NA	ADC-30
GCA_028753295.1_ASM2875329v1_genomic	CP102580.1	Missing	Missing	Missing	Missing	Missing	Missing	2	IC2	2	2	NA	NA	ADC-30
GCA_045256945.1_G636_genomic	CP174090.1	USA: Atlanta, GA	United States	2018	2018	Respiratory sample	Respiratory_tract	2	IC2	2	3	NA	NA	ADC-30
GCA_003949595.2_ASM394959v2_genomic	CP039993.1	USA: Arizona	United States	2011-07-19	2011.5	trach asp	Respiratory_tract	2	IC2	2	2	NA	NA	ADC-25
GCA_003948535.2_AS	CP039520.1	USA: Arizona	United States	2011-10-15	2011.8	trach asp	Respiratory_tract	2	IC2	2	1	NA	NA	ADC-25

M394853v2_genomic														
GCA_025853995.1_ASM2585399v1_genomic	CP107587.1	USA	United States	2018-08-14	2018.6	skin	Skin	2	IC2	2	2	NA	NA	ADC-25
GCA_025854015.1_ASM2585401v1_genomic	CP107590.1	USA	United States	2018-08-14	2018.6	skin	Skin	2	IC2	2	2	NA	NA	ADC-25
GCA_025854155.1_ASM2585415v1_genomic	CP107605.1	USA	United States	2018-10-14	2018.8	ENVIRONMENT	ENVIRONMENT	2	IC2	2	2	NA	NA	ADC-25
GCA_023273785.1_ASM2327378v1_genomic	CP096892.1	China	China	2015	2015	Respiratory_tract	Respiratory_tract	2	IC2	2	2	NA	NA	ADC-25
GCA_023517175.1_ASM2351717v1_genomic	CP077828.1	China: Hangzhou, Sir Run Run Shaw Hospital ICU	China	2019-10-22	2019.8	Ventilator shelf_P101_BU22	Hospital_enviroment	2	IC2	2	1	NA	NA	ADC-25
GCA_003591615.1_ASM359161v1_genomic	CP023140.1	China:Zhejiang	China	2016	2016	blood	Blood	922	IC2	2	0	NA	NA	ADC-25
GCA_043041525.1_ASM4304152v1_genomic	CP134549.1	China: Shanghai	China	2010	2010	wound	Skin	2	IC2	2	0	NA	NA	ADC-25
GCA_001573085.1_ASM157308v1_genomic	CP014539.1	China: Hubei	China	2009	2009	wound	Skin	2	IC2	2	0	NA	NA	ADC-25
GCA_002762155.1_ASM276215v1_genomic	CP024612.1	China: Beijing	China	2011-03-21	2011.2	sputum	Respiratory_tract	2	IC2	2	0	NA	NA	ADC-25

GCA_002950495.2_ASM295049v2_genomic	CP026750.2	China: Sichuan, Chengdu	China	Missing	Missing	Missing	Missing	2	IC2	2	2	OXA-58 # floR # sul2	NA	ADC-25
GCA_047223335.1_ASM4722333v1_genomic	CP178386.1	Nigeria: Ibadan, Oyo State	Nigeria	2023-04	2023.2	soil/water/Rectum	Missing	2	IC2	2	0	NA	NA	ADC-25
GCA_043038945.1_ASM4303894v1_genomic	CP134540.1	China: Hebei	China	2010	2010	sputum	Respiratory_tract	2	IC2	2	3	OXA-23	NA	ADC-25
GCA_045278855.1_ASM4527885v1_genomic	CP134599.1	China: Anhui	China	2010	2010	sputum	Respiratory_tract	2	IC2	2	1	NA	NA	ADC-30
GCA_018454385.1_ASM1845438v1_genomic	CP075321.1	China: Lushi County, Sanmenxia City, Henan Provience	China	2021-01-12	2021.0	hospital	Hospital_enviroment	2	IC2	2	1	NA	NA	ADC-30
GCA_023517155.1_ASM2351715v1_genomic	CP077826.1	China: Hangzhou, Sir Run Run Shaw Hospital ICU	China	2019-08-27	2019.7	Bedside table_P53_BU18	Hospital_enviroment	2	IC2	2	1	NA	NA	ADC-30
GCA_045678795.1_ASM4567879v1_genomic	CP053034.1	China:Nanjing	China	2018-03-20	2018.2	sputum	Respiratory_tract	2	IC2	2	1	NA	NA	ADC-30
GCA_012272735.1_ASM1227273v1_genomic	CP050907.1	China: Beijing	China	2017	2017	sputum	Respiratory_tract	2	IC2	2	3	NA	NA	ADC-30
GCA_012272715.1_AS	CP050904.1	China: Beijing	China	2019	2019	Bronchoalveolar lavage fluid	Respiratory_tract	2	IC2	2	2	NA	NA	ADC-30

M1227271v1_genomic														
GCA_023362255.1_ASM2336225v1_genomic	CP096766.1	China:Jiangsu	China	2005-07-01	2005.5	sputum	Respiratory_tract	2	IC2	2	1	NA	NA	ADC-30
GCA_043050195.1_ASM4305019v1_genomic	CP134574.1	China: Shandong	China	2010	2010	sputum	Respiratory_tract	2	IC2	2	2	OXA-23	NA	ADC-30
GCA_043056065.1_ASM4305606v1_genomic	CP134597.1	China: Anhui	China	2010	2010	sputum	Respiratory_tract	2	IC2	2	1	NA	NA	ADC-30
GCA_000820685.1_CLCgenomics_genomic	HG977526.1	Missing	Missing	Missing	Missing	Missing	Missing	2	IC2	2	3	NA	NA	ADC-30
GCA_000820665.1_CLCgenomics_genomic	HG977522.1	Missing	Missing	Missing	Missing	Missing	Missing	2	IC2	2	3	NA	NA	ADC-30
GCA_022460015.1_ASM2246001v1_genomic	CP091371.1	Belgium	Belgium	Missing	Missing	Missing	Missing	2	IC2	2	0	NA	NA	ADC-30
GCA_000226275.2_ASM22627v2_genomic	CP001937.2	China: Zheijiang	China	2006	2006	Missing	Missing	2	IC2	2	0	NA	NA	ADC-30
GCA_043040285.1_ASM4304028v1_genomic	CP134547.1	China: Hubei	China	2010	2010	sputum	Respiratory_tract	2	IC2	2	0	NA	NA	ADC-30
GCA_043044755.1_ASM4304475v1_genomic	CP134558.1	China: Henan	China	2010	2010	secretion	Secretion	2	IC2	2	1	NA	pilF	ADC-30
GCF_038627275.1_AS	NZ_CP157260.1	USA	United States	2024-04-12	2024.3	Missing	Missing	2	IC2	2	1	NA	NA	ADC-30

M3862727v2_genomic														
GCA_038627275.2_ASM3862727v2_genomic	CP157260.1	USA	United States	2024-04-12	2024.3	Missing	Missing	2	IC2	2	1	NA	NA	ADC-30
GCF_038627255.1_ASM3862725v2_genomic	NZ_CP157342.1	USA	United States	2024-04-12	2024.3	Missing	Missing	2	IC2	2	1	NA	NA	ADC-30
GCA_038627255.2_ASM3862725v2_genomic	CP157342.1	USA	United States	2024-04-12	2024.3	Missing	Missing	2	IC2	2	1	NA	NA	ADC-30
GCF_038522965.1_ASM3852296v2_genomic	NZ_CP157344.1	USA	United States	2024-04-12	2024.3	Missing	Missing	2	IC2	2	1	NA	NA	ADC-30
GCA_038522965.2_ASM3852296v2_genomic	CP157344.1	USA	United States	2024-04-12	2024.3	Missing	Missing	2	IC2	2	1	NA	NA	ADC-30
GCA_001922225.1_ASM192222v1_genomic	CP016298.1	USA:VA	United States	Missing	Missing	sputum	Respiratory_tract	2	IC2	2	1	NA	NA	ADC-30
GCA_001922245.1_ASM192224v1_genomic	CP016300.1	USA:VA	United States	Missing	Missing	Missing	Missing	2	IC2	2	2	OXA-23	NA	ADC-162
GCA_001922205.1_ASM192220v1_genomic	CP016295.1	USA:VA	United States	Missing	Missing	BAL	Respiratory_tract	2	IC2	2	2	APH(3')-VIa # OXA-23	NA	ADC-162
GCA_003931775.1_ASM393177v1_genomic	CP034243.1	Canada	Canada	2016	2016	Missing	Missing	2	IC2	2	0	NA	NA	ADC-30
GCA_003931755.1_AS	CP034242.1	Canada	Canada	2016	2016	Missing	Missing	2	IC2	2	0	NA	NA	ADC-30

M393175v1_genomic														
GCA_003948455.2_ASM394845v2_genomic	CP039518.1	USA: Arizona	United States	2011-10-15	2011.8	BAL	Respiratory_tract	2	IC2	2	1	OXA-23	NA	ADC-30
GCA_003949295.2_ASM394929v2_genomic	CP036283.1	USA: Arizona	United States	2013-04-18	2013.3	sputum	Respiratory_tract	NA	NA	NA	3	OXA-23	lpxD # lpxA	ADC-30
GCA_001895985.1_ASM189598v1_genomic	CP018256.1	USA: Arizona	United States	2008-03-20	2008.2	sputum	Respiratory_tract	2	IC2	2	1	NA	NA	ADC-30
GCA_040939585.2_ASM4093958v2_genomic	CP168448.1	USA	United States	2024-07-10	2024.5	blood	Blood	2	IC2	2	1	NA	NA	ADC-30
GCA_044827985.2_ASM4482798v2_genomic	CP180029.1	USA	United States	2024-10-28	2024.8	skin	Skin	2	IC2	2	1	NA	NA	ADC-30
GCA_002948475.1_ASM294847v1_genomic	CP026943.1	USA: Pittsburgh	United States	2007-01-09	2007.0	BAL	Respiratory_tract	2	IC2	2	3	OXA-23	NA	ADC-162
GCA_034126905.2_ASM3412690v2_genomic	CP143346.1	USA	United States	2023-11-14	2023.9	skin	Skin	new_alleles	NA	NA	1	NA	NA	ADC-30
GCF_034126905.1_ASM3412690v2_genomic	NZ_CP143346.1	USA	United States	2023-11-14	2023.9	skin	Skin	new_alleles	NA	NA	1	NA	NA	ADC-30
GCF_034126645.1_ASM3412664v2_genomic	NZ_CP143344.1	USA	United States	2023-11-14	2023.9	skin	Skin	new_alleles	NA	NA	1	NA	NA	ADC-30
GCA_034126645.2_AS	CP143344.1	USA	United States	2023-11-14	2023.9	skin	Skin	new_alleles	NA	NA	1	NA	NA	ADC-30

M3412664v 2_genomic														
GCA_04364 7355.2_AS M4364735v 2_genomic	CP180092.1	USA	United States	2024-10-08	2024.8	Rectal Swab	Digestive_system	2	IC2	2	1	NA	NA	ADC-30
GCA_04453 7505.2_AS M4453750v 2_genomic	CP180036.1	USA	United States	2024-10-15	2024.8	Rectal Swab	Digestive_system	2	IC2	2	1	NA	NA	ADC-30
GCA_00093 9415.2_HG AP_assembl y_of_Acinet obacter_ba umannii_OR AB01_geno mic	CP015483.1	USA: Lincoln County, Oregon	United States	2012-06-14	2012.5	bodily fluid	Secretion	2	IC2	2	3	sul2 # OXA-237	NA	ADC-30
GCA_05050 0295.1_WM 99a_Hybrid _Assembly_ genomic	CP191379.1	Australia: Sydney	Australia	1999	1999	Missing	Missing	2	IC2	2	2	NA	NA	ADC-30
GCA_02245 9135.1_AS M2245913v 1_genomic	CP091363.1	Belgium	Belgium	Missing	Missing	Missing	Missing	2	IC2	2	0	NA	NA	ADC-30
GCA_01990 3215.1_AS M1990321v 1_genomic	CP059548.1	France	France	Missing	Missing	Seine River	Water	2	IC2	2	0	NA	NA	ADC-30
GCA_00352 2785.1_AS M352278v1 _genomic	CP023026.1	Mexico: Torreon, Coahuila	Mexico	2011-04-10	2011.3	Secretion	Secretion	2	IC2	2	2	OXA-72 # APH(3')-VIa	NA	ADC-73
GCA_00190 2375.1_AS M190237v1 _genomic	CP018421.1	China: Beijing	China	2007-05-18	2007.4	medical patient	Clinical_sample	2	IC2	2	1	NA	NA	ADC-30

GCA_043055495.1_ASM4305549v1_genomic	CP134596.1	China: Anhui	China	2010	2010	sputum	Respiratory_tract	2	IC2	2	0	NA	NA	ADC-30
GCA_000419385.1_ASM41938v1_genomic	CP003846.1	Missing	Missing	Missing	Missing	Missing	Missing	2	IC2	2	2	AAC(6')-Ib9 # catB8 # aadA # qacEdelta1 # sul1 # armA # msrE # mphE # APH(3')-Ia	NA	ADC-30
GCA_043049525.1_ASM4304952v1_genomic	CP134571.1	China: Heilongjiang	China	2010	2010	sputum	Respiratory_tract	2	IC2	2	2	OXA-23	NA	ADC-140
GCA_003516005.1_ASM351600v1_genomic	CP023031.1	Mexico: Monterrey, Nuevo Leon	Mexico	2008-04-28	2008.3	blood	Blood	1544	IC2	2	2	OXA-58	NA	ADC-30
GCA_023362215.1_ASM2336221v1_genomic	CP096762.1	China:Liaoning	China	2005-07-01	2005.5	sputum	Respiratory_tract	2	IC2	3	1	NA	NA	ADC-30
GCA_043048875.1_ASM4304887v1_genomic	CP134570.1	China: Sichuan	China	2010	2010	bronchial lavage fluid	Respiratory_tract	2	IC2	2	0	NA	NA	ADC-30
GCA_003202135.1_ASM320213v1_genomic	CP021496.1	China:Zhejiang	China	2009-06-01	2009.4	Missing	Missing	2	IC2	2	0	NA	NA	ADC-73
GCA_023361955.1_ASM2336195v1_genomic	CP096731.1	China:Liaoning	China	2016-05-05	2016.3	blood	Blood	2	IC2	2	2	NA	NA	ADC-30
GCA_030585325.1_AS	CP043469.1	USA: WI	United States	2018	2018	Skin/Wound	Skin	2	IC2	2	3	OXA-72 # OXA-72	plcD	ADC-30

M3058532v 1_genomic														
GCA_030585225.1_ASM3058522v1_genomic	CP043452.1	USA: WI	United States	2018	2018	Skin/Wound	Skin	2	IC2	2	2	OXA-72 # OXA-72	NA	ADC-30
GCA_030585245.1_ASM3058524v1_genomic	CP043455.1	USA: WI	United States	2018	2018	Skin/Wound	Skin	2	IC2	2	2	OXA-72 # OXA-72	NA	ADC-30
GCA_030585285.1_ASM3058528v1_genomic	CP043462.1	USA: WI	United States	2018	2018	Skin/Wound	Skin	2	IC2	2	2	OXA-72 # OXA-72	NA	ADC-30
GCA_030585305.1_ASM3058530v1_genomic	CP043465.1	USA: WI	United States	2018	2018	Skin/Wound	Skin	2	IC2	2	3	OXA-72 # OXA-72	NA	ADC-30
GCA_033189955.1_ASM3318995v1_genomic	CP136169.1	France: Marcy l'etoile	France	2021-12-07	2021.9	oral swab	Respiratory_tract	2	IC2	2	1	NA	NA	ADC-30
GCA_023361895.1_ASM2336189v1_genomic	CP096724.1	China:Beijing	China	2010-06-29	2010.5	blood	Blood	2	IC2	2	2	OXA-23	NA	ADC-30
GCA_002082645.1_ASM208264v1_genomic	CP020586.1	South Korea: Chungbuk	Korea, Republic of	2013-10	2013.8	sputum	Respiratory_tract	2	IC2	2	1	NA	NA	ADC-30
GCA_003010655.1_ASM301065v1_genomic	CP027607.1	Missing	Missing	Missing	Missing	Missing	Missing	2	IC2	2	2	NA	NA	ADC-30
GCA_023361255.1_ASM2336125v1_genomic	CP096707.1	China:Xinjiang	China	2012-05-29	2012.4	sputum	Respiratory_tract	2	IC2	2	2	OXA-23	NA	ADC-30
GCA_042209715.2_AS	CP174045.1	USA	United States	2024-08-23	2024.6	skin	Skin	2	IC2	2	1	NA	NA	ADC-30

M4220971v2_genomic														
GCA_043647605.2_ASM4364760v2_genomic	CP180101.1	USA	United States	2024-09-29	2024.7	skin	Skin	2	IC2	2	1	NA	NA	ADC-30
GCA_013376855.2_ASM1337685v2_genomic	CP056784.2	USA	United States	2016	2016	human clinical sample	Clinical_sample	570	IC2	3	1	NA	NA	ADC-73
GCA_015870765.1_ASM1587076v1_genomic	CP060011.1	USA	United States	2016	2016	human clinical sample	Clinical_sample	570	IC2	3	1	NA	NA	ADC-73
GCA_015870965.1_ASM1587096v1_genomic	CP060013.1	USA	United States	2016	2016	human clinical sample	Clinical_sample	570	IC2	3	1	NA	NA	ADC-73
GCA_019356095.1_ASM1935609v1_genomic	CP079945.1	China:Beijing	China	2019-02-22	2019.1	blood	Blood	2	IC2	3	0	NA	NA	ADC-73
GCA_029713825.1_ASM2971382v1_genomic	CP121581.1	Saudi Arabia:Riyadh	Saudi Arabia	2018	2018	Missing	Missing	2	IC2	2	1	msrE # mphE	NA	ADC-30
GCA_029713785.1_ASM2971378v1_genomic	CP121563.1	Saudi Arabia:Riyadh	Saudi Arabia	2019	2019	Missing	Missing	570	IC2	3	3	APH(3')-VIa	NA	ADC-73
GCA_029713965.1_ASM2971396v1_genomic	CP121595.1	Saudi Arabia:Medina	Saudi Arabia	2019	2019	Missing	Missing	570	IC2	3	2	APH(3')-VIa	NA	ADC-73
GCA_000189735.2_ASM18973v2_genomic	CP002522.2	Missing	Missing	Missing	Missing	Missing	Missing	2	IC2	2	2	NA	NA	ADC-30
GCA_016903215.1_AS	CP069851.1	USA:MD	United States	Missing	Missing	Missing	Missing	2	IC2	2	1	NA	NA	ADC-30

M1690321v 1_genomic														
GCA_02246 0575.1_AS M2246057v 1_genomic	CP091375.1	Belgium	Belgium	Missing	Missing	Missing	Missing	2	IC2	2	0	NA	NA	ADC-30
GCA_04304 7475.1_AS M4304747v 1_genomic	CP134565.1	China: Henan	China	2010	2010	secretion	Secretion	2	IC2	3	0	NA	NA	ADC-30
GCA_01635 0085.1_AS M1635008v 1_genomic	CP066237.1	South Korea: Hwaseong -si	Slovenia	2020-04-11	2020.3	blood	Blood	2	IC2	2	3	NA	NA	ADC-30
GCA_01635 0005.1_AS M1635000v 1_genomic	CP066229.1	South Korea: Hwaseong -si	Slovenia	2020-05-16	2020.4	blood	Blood	2	IC2	2	2	NA	NA	ADC-30
GCA_04568 9765.1_AS M4568976v 1_genomic	CP146828.1	South Korea	Korea, Republic of	2020-05-16	2020.4	Missing	Missing	2	IC2	2	3	NA	NA	ADC-30
GCA_01635 0105.1_AS M1635010v 1_genomic	CP066235.1	South Korea: Hwaseong -si	Slovenia	2020-05-07	2020.3	blood	Blood	2	IC2	2	1	NA	NA	ADC-30
GCA_01635 0065.1_AS M1635006v 1_genomic	CP066232.1	South Korea: Hwaseong -si	Slovenia	2020-05-08	2020.4	blood	Blood	2	IC2	2	2	NA	NA	ADC-30
GCA_04568 9745.1_AS M4568974v 1_genomic	CP146769.1	South Korea	Korea, Republic of	2020-03-30	2020.2	Missing	Missing	2	IC2	2	7	NA	NA	ADC-30
GCA_00208 2885.1_AS M208288v1 _genomic	CP020581.1	South Korea: Seoul	Korea, Republic of	2013-10	2013.8	Bronchial washing fluid	Respiratory_tract	2	IC2	2	1	NA	NA	ADC-30

GCA_002082845.1_ASM208284v1_genomic	CP020579.1	South Korea: Seoul	Korea, Republic of	2013-09	2013.7	blood	Blood	187	IC2	2	0	NA	NA	ADC-162
GCA_045278645.1_ASM4527864v1_genomic	CP137069.1	South Korea: Republic of	Korea, Republic of	2015-02-23T01:50Z	2015.1	Seoul National University Bundang Hospital Intensive Care Unit	Hospital_enviroment	2	IC2	2	1	NA	NA	ADC-30
GCA_036287275.1_ASM3628727v1_genomic	CP142895.1	South Korea	Korea, Republic of	2020-07-02	2020.5	Missing	Missing	2	IC2	2	3	NA	NA	ADC-30
GCA_045289525.1_ASM4528952v1_genomic	CP146831.1	South Korea	Korea, Republic of	2020-08-28	2020.7	Missing	Missing	2	IC2	2	5	NA	NA	ADC-30
GCA_045278635.1_ASM4527863v1_genomic	CP137071.1	South Korea: Republic of	Korea, Republic of	2017-05-23T11:57Z	2017.4	Seoul National University Bundang Hospital Intensive Care Unit	Hospital_enviroment	2	IC2	2	1	OXA-23	NA	ADC-30
GCA_002082625.2_ASM208262v2_genomic	CP020584.1	South Korea: Jeonbuk	Korea, Republic of	2013-10	2013.8	sputum	Respiratory_tract	2	IC2	2	1	NA	NA	ADC-30
GCA_024138105.1_ASM2413810v1_genomic	CP099989.1	South Korea	Korea, Republic of	2016-04-25	2016.3	blood	Blood	2	IC2	2	0	NA	NA	ADC-30
GCA_001806465.1_ASM180646v1_genomic	CP017654.1	South Korea:Jeonju	Korea, Republic of	2015-09-10	2015.7	sputum	Respiratory_tract	2	IC2	2	1	NA	NA	ADC-30

GCA_001806405.1_ASM180640v1_genomic	CP017648.1	South Korea:Busan	Korea, Republic of	2015-10-08	2015.8	sputum	Respiratory_tract	2	IC2	2	1	NA	NA	ADC-30
GCA_002082865.1_ASM208286v1_genomic	CP020578.1	South Korea: Seoul	Korea, Republic of	2013-09	2013.7	blood	Blood	2	IC2	2	2	NA	NA	ADC-30
GCA_012272775.1_ASM1227277v1_genomic	CP050914.1	China: Beijing	China	2017	2017	sputum	Respiratory_tract	2	IC2	2	1	NA	NA	ADC-30
GCA_002762095.1_ASM276209v1_genomic	CP024611.1	China: Beijing	China	2011-07-07	2011.5	sputum	Respiratory_tract	2	IC2	2	0	NA	NA	ADC-30
GCA_002762115.1_ASM276211v1_genomic	CP024613.1	China: Beijing	China	2011-02-09	2011.1	sputum	Respiratory_tract	2	IC2	2	0	NA	NA	ADC-30
GCA_002082705.1_ASM208270v1_genomic	CP020574.1	South Korea	Korea, Republic of	2013-01	2013.0	Pulmonary	Respiratory_tract	2	IC2	2	2	NA	NA	ADC-30
GCA_001543995.1_ASM154399v1_genomic	CP014215.1	Missing	Missing	2014-05	2014.3	sputum	Respiratory_tract	2	IC2	2	2	NA	NA	ADC-30 # ADC-30
GCA_002843665.1_ASM284366v1_genomic	CP025266.1	South Korea: Seoul	Korea, Republic of	2012-04-12	2012.3	Blood, Central line	Blood	2	IC2	2	1	NA	NA	ADC-30
GCA_001514375.1_ASM151437v1_genomic	CP013924.1	South Korea:Daegu	Korea, Republic of	2012-02-13	2012.1	pus	Skin	2	IC2	2	1	NA	NA	ADC-115
GCA_045278665.1_ASM4527866v1_genomic	CP137064.1	South Korea: Republic of	Korea, Republic of	2016-09-07T12:14Z	2016.7	Seoul National University Bundang	Hospital_enviroment	2	IC2	2	2	NA	NA	ADC-30

						Hospital Intensive Care Unit								
GCA_045289885.1_ASM4528988v1_genomic	CP142645.1	South Korea	Korea, Republic of	2020-05-06	2020.3	human	human	2	IC2	2	3	NA	NA	ADC-30
GCA_041002685.1_ASM4100268v1_genomic	CP163254.1	South Korea: Seoul	Korea, Republic of	2019-04-09	2019.3	blood	Blood	2	IC2	2	0	NA	NA	ADC-30
GCA_041002735.1_ASM4100273v1_genomic	CP163255.1	South Korea: Seoul	Korea, Republic of	2019-02-20	2019.1	blood	Blood	2	IC2	2	0	NA	NA	ADC-30
GCA_041002475.1_ASM4100247v1_genomic	CP163253.1	South Korea: Seoul	Korea, Republic of	2019-08-14	2019.6	blood	Blood	2	IC2	2	0	NA	NA	ADC-30
GCA_037039585.1_ASM3703958v1_genomic	CP146229.1	South Korea	Korea, Republic of	2020-01-15	2020.0	human	human	2	IC2	2	1	OXA-23	NA	ADC-30
GCA_045290085.1_ASM4529008v1_genomic	CP142636.1	South Korea	Korea, Republic of	2020-01-08	2020.0	human	human	2	IC2	2	6	OXA-1223	NA	ADC-30
GCA_043054745.1_ASM4305474v1_genomic	CP134595.1	China: Anhui	China	2010	2010	sputum	Respiratory_tract	2	IC2	2	0	NA	NA	ADC-30
GCA_040957045.1_ASM4095704v1_genomic	CP162563.1	Thailand: northern region	Thailand	2016/2017	2016	Pus	Skin	2	IC2	3	2	NA	NA	ADC-73
GCA_040956955.1_ASM4095695v1_genomic	CP162561.1	Thailand: northern region	Thailand	2016/2017	2016	sputum	Respiratory_tract	2	IC2	3	2	NA	NA	ADC-73

GCA_040956905.1_ASM4095690v1_genomic	CP162554.1	Thailand: northeastern region	Thailand	2016/2017	2016	sputum	Respiratory_tract	2	IC2	3	3	NA	NA	ADC-73
GCA_040956925.1_ASM4095692v1_genomic	CP162558.1	Thailand: northern region	Thailand	2016/2017	2016	sputum	Respiratory_tract	2	IC2	3	1	NA	NA	ADC-73
GCA_040957165.1_ASM4095716v1_genomic	CP162568.1	Thailand: northern region	Thailand	2016/2017	2016	sputum	Respiratory_tract	2	IC2	3	1	NA	NA	ADC-73
GCA_012974845.1_ASM1297484v1_genomic	CP041148.1	Thailand: Bangkok	Thailand	2017-05	2017.3	urine	Urinary_system	2	IC2	2	1	NA	NA	ADC-30
GCA_040822405.1_ASM4082240v1_genomic	CP161992.1	Singapore	Singapore	2008-09-30	2008.7	Missing	Missing	2	IC2	2	2	OXA-23	NA	ADC-30
GCA_017098185.2_ASM1709818v2_genomic	CP059355.1	China: Beijing	China	2016-02-03	2016.1	sputum	Respiratory_tract	2	IC2	3	1	OXA-23	NA	ADC-73
GCA_013305465.1_ASM1330546v1_genomic	CP054302.1	Australia: Brisbane	Australia	2016	2016	Urine (catheter)	Urinary_system	2	IC2	2	0	NA	NA	ADC-30
GCA_029714065.1_ASM2971406v1_genomic	CP121621.1	Saudi Arabia:Jeddah	Saudi Arabia	2018	2018	Missing	Missing	2	IC2	2	3	NA	NA	ADC-30
GCA_021387855.2_ASM2138785v2_genomic	CP097878.1	China	China	2018	2018	Surface	Surface	2	IC2	2	3	OXA-23	NA	ADC-30
GCA_021387885.2_ASM2138788v2_genomic	CP097875.1	China	China	2018	2018	Surface	Surface	2	IC2	2	1	NA	NA	ADC-30

GCA_028201695.1_ASM2820169v1_genomic	CP116442.1	China:Hengyang	China	2022-11-13	2022.9	Labatory, 37662RM1 cultured in medium without carbapenem	Lab	2	IC2	2	1	NA	NA	NA
GCA_028201675.1_ASM2820167v1_genomic	CP116440.1	China:Hengyang	China	2022-11-13	2022.9	Labatory, 37662RM2 cultured in medium without carbapenem	Lab	new_alleles	NA	NA	1	NA	NA	ADC-30
GCA_028464005.1_ASM2846400v1_genomic	CP116801.1	China: Guangzhou	China	2016-03	2016.2	selected with meropenem showing different genome comparing to wild type (37662WT)	Missing	2	IC2	2	1	NA	NA	ADC-30
GCA_025402915.1_ASM2540291v1_genomic	CP104751.1	China: Meizhou	China	2012-05	2012.3	sputum of an intensive care unit patient at Meizhou People's Hospital	Respiratory_tract	2	IC2	2	1	NA	NA	ADC-30
GCA_028464025.1_ASM2846402v1_genomic	CP116803.1	China: Guangzhou	China	2016-03	2016.2	selected with meropenem showing different genome comparing to wild type (37662WT)	Missing	2	IC2	2	1	NA	NA	ADC-30
GCA_001573125.1_ASM157312v1_genomic	CP014541.1	China: Guangdong	China	2010	2010	drainage fluid	Missing	2	IC2	2	0	NA	NA	ADC-30
GCA_021730415.1_AS	CP091452.1	Thailand:Eastern	Thailand	2015	2015	sputum	Respiratory_tract	98	IC2	3	0	NA	NA	ADC-73

M2173041v1_genomic		region hospital												
GCA_029581555.1_ASM2958155v1_genomic	CP120894.1	Russia:Moscow	Russian Federation	2016-02-11	2016.1	sputum	Respiratory_tract	2	IC2	2	2	OXA-72 # OXA-72	NA	ADC-30
GCA_025732115.1_ASM2573211v1_genomic	CP107034.1	Russia:Moscow	Russian Federation	2019	2019	upper respiratory tract	Respiratory_tract	2	IC2	2	1	OXA-72 # OXA-72	NA	ADC-30
GCA_024749545.1_ASM2474954v1_genomic	CP087321.1	Germany	Germany	Missing	Missing	Missing	Missing	2	IC2	2	3	OXA-72 # OXA-72 # OXA-72 # OXA-72 # OXA-72 # OXA-72 # OXA-72 # OXA-72	NA	ADC-56
GCA_029714125.1_ASM2971412v1_genomic	CP121615.1	Saudi Arabia:Jeddah	Saudi Arabia	2018	2018	Missing	Missing	2	IC2	2	5	OXA-72 # OXA-72 # msrE # mphE # OXA-72 # OXA-23 # APH(3')-VIa	NA	ADC-30
GCA_029713865.1_ASM2971386v1_genomic	CP121609.1	Saudi Arabia:Jeddah	Saudi Arabia	2019	2019	Missing	Missing	2	IC2	2	2	mphE # msrE # OXA-72 # OXA-72 # OXA-72	NA	ADC-30
GCA_029714045.1_ASM2971404v1_genomic	CP121612.1	Saudi Arabia:Jeddah	Saudi Arabia	2018	2018	Missing	Missing	1572	Not IC	NA	2	NA	NA	ADC-73
GCA_005280695.1_ASM528069v1_genomic	CP040084.1	India	India	2018	2018	blood	Blood	2	IC2	2	2	NA	csuA/B # csuA # csuB # csuC # csuD # csuE	ADC-30

GCA_022468645.2_ASM2246864v2_genomic	CP091351.1	Belgium	Belgium	2017	2017	Missing	Missing	2	IC2	2	1	OXA-72	NA	ADC-30
GCA_900088705.1_26872B01_genomic	LT594095.1	Missing	Missing	Missing	Missing	Missing	Missing	1550	IC2	2	1	NA	NA	ADC-82
GCA_046097545.1_BAL114_Hybrid_assembly_genomic	CP175804.1	Viet Nam	Viet Nam	2009	2009	Missing	Missing	2	IC2	2	1	NA	NA	ADC-82
GCA_050030175.1_ASM5003017v1_genomic	CP179696.1	Paraguay: Itaugua	Paraguay	2024-02-07	2024.1	tracheal secretion	Respiratory_tract	2	IC2	3	2	NA	NA	ADC-73
GCA_048067305.1_ASM4806730v1_genomic	CP179702.1	Paraguay: Itaugua	Paraguay	2024-02-05	2024.1	tracheal secretion	Respiratory_tract	2	IC2	3	2	NA	NA	ADC-73
GCA_048480215.1_ASM4848021v1_genomic	CP179699.1	Paraguay: Itaugua	Paraguay	2024-01-07	2024.0	tracheal secretion	Respiratory_tract	2	IC2	3	2	NA	NA	ADC-73
GCA_022468075.1_ASM2246807v1_genomic	CP091345.1	Belgium	Belgium	Missing	Missing	Missing	Missing	2	IC2	2	0	NA	NA	ADC-30
GCA_022468295.1_ASM2246829v1_genomic	CP091347.1	Belgium	Belgium	Missing	Missing	Missing	Missing	2	IC2	2	0	NA	NA	ADC-30
GCA_033170255.1_ASM3317025v1_genomic	CP137021.1	Italy	Italy	2022-05-30	2022.4	blood	Blood	2	IC2	2	0	NA	NA	ADC-73
GCF_000187205.2_AS	NC_017847.1	Missing	Missing	Missing	Missing	Missing	Missing	2	IC2	2	2	NA	NA	ADC-82

M18720v4_genomic														
GCA_000187205.4_ASM18720v4_genomic	CP003500.1	Missing	Missing	Missing	Missing	Missing	Missing	2	IC2	2	2	NA	NA	ADC-82
GCA_043039655.1_ASM4303965v1_genomic	CP134544.1	China: Hebei	China	2010	2010	urine	Urinary_system	2	IC2	2	2	OXA-23	NA	ADC-82
GCA_041004195.1_W155_genomic	CP163039.1	China:Kunming	China	2020-01	2020.0	feces	Digestive_system	2	IC2	3	2	NA	NA	ADC-73
GCA_008244885.1_ASM824488v1_genomic	CP043417.1	Canada	Canada	2013	2013	Missing	Missing	2	IC2	2	0	NA	NA	ADC-30
GCA_004101685.1_ASM410168v1_genomic	CP035184.1	Canada	Canada	2013	2013	Missing	Missing	2	IC2	2	0	NA	NA	ADC-30
GCA_004101725.1_ASM410172v1_genomic	CP035185.1	Canada	Canada	2012	2012	Missing	Missing	2	IC2	2	0	NA	NA	ADC-30
GCA_008244905.1_ASM824490v1_genomic	CP043418.1	Canada	Canada	2013	2013	Missing	Missing	2	IC2	2	0	NA	NA	ADC-30
GCA_004101705.1_ASM410170v1_genomic	CP035183.1	Canada	Canada	2014	2014	Missing	Missing	2	IC2	2	0	NA	NA	ADC-30
GCA_008244865.1_ASM824486v1_genomic	CP043419.1	Canada	Canada	2012	2012	Missing	Missing	2	IC2	2	0	NA	NA	ADC-30
GCA_004101745.1_AS	CP035186.1	Canada	Canada	2012	2012	Missing	Missing	2	IC2	2	0	NA	NA	ADC-30

M410174v1_genomic														
GCA_042140315.1_Hv383_genomic	CP169770.1	Mexico	Mexico	2018	2018	Urine/Urinary tract	Urinary_system	2	IC2	2	2	OXA-72 # OXA-72	NA	ADC-73
GCA_003522885.1_ASM352288v1_genomic	CP023034.1	Mexico: San Luis Potosi	Mexico	2009-12-31	2009.9	wound	Skin	2	IC2	2	1	OXA-72	NA	ADC-30
GCA_036870965.1_ASM3687096v1_genomic	CP145430.1	South Korea	Korea, Republic of	2020-01-23	2020.1	Missing	Missing	2	IC2	3	6	NA	NA	ADC-73
GCA_043054075.1_ASM4305407v1_genomic	CP134594.1	China: Xinjiang	China	2010	2010	sputum	Respiratory_tract	2	IC2	2	2	OXA-23	NA	ADC-30
GCA_025732095.1_ASM2573209v1_genomic	CP107032.1	Russia:Moscow	Russian Federation	2019	2019	upper respiratory tract	Respiratory_tract	2	IC2	2	1	NA	NA	ADC-30
GCA_033378915.2_ASM3337891v2_genomic	CP157866.1	Russia:Moscow	Russian Federation	2019-10-15	2019.8	cerebrospinal fluid	CNS	2	IC2	2	1	NA	NA	ADC-30
GCA_029814335.1_ASM2981433v1_genomic	CP122365.1	Russia:Moscow	Russian Federation	2019-07-29	2019.6	blood	Blood	2	IC2	2	2	APH(3')-VIa	NA	ADC-30
GCA_041154815.1_ASM4115481v1_genomic	AP031580.1	Nepal:Kathmandu	Nepal	2019	2019	Missing	Missing	2	IC2	3	0	NA	NA	ADC-73
GCA_029713985.1_ASM2971398v1_genomic	CP121604.1	Saudi Arabia:Medina	Saudi Arabia	2019	2019	Missing	Missing	2	IC2	2	4	NA	NA	ADC-30
GCA_004136355.1_AS	CP031444.1	USA: North Carolina,	United States	2012-08-22	2012.6	right leg muscle, right leg fascia,	Missing	2	IC2	2	2	msrE # mphE #	NA	ADC-56

M413635v1_genomic		Chapel Hill				central venous catheter, dialysis catheter, surface of the left leg ulcer, and left lower leg						OXA-72 # OXA-72		
GCA_022467975.1_ASM2246797v1_genomic	CP091344.1	Belgium	Belgium	Missing	Missing	Missing	Missing	2	IC2	2	0	NA	NA	ADC-30
GCA_022459175.1_ASM2245917v1_genomic	CP091365.1	Belgium	Belgium	Missing	Missing	Missing	Missing	2	IC2	2	0	NA	NA	ADC-30
GCA_022459295.1_ASM2245929v1_genomic	CP091366.1	Belgium	Belgium	Missing	Missing	Missing	Missing	2	IC2	2	0	NA	NA	ADC-30
GCA_022461025.2_ASM2246102v2_genomic	CP091378.1	Belgium	Belgium	2017	2017	Missing	Missing	2	IC2	2	1	OXA-58	NA	ADC-56
GCA_044232175.1_ASM4423217v1_genomic	CP048102.1	Nepal	Nepal	2017	2017	Missing	Missing	2	IC2	2	0	NA	NA	ADC-30
GCA_005280375.1_ASM528037v1_genomic	CP040047.1	India	India	2019	2019	blood	Blood	NA	NA	NA	1	NA	NA	ADC-73
GCA_012934925.1_ASM1293492v1_genomic	CP050388.1	India	India	2019	2019	sputum	Respiratory_tract	2	IC2	3	1	NA	NA	ADC-73
GCA_029714105.1_AS	CP121567.1	Saudi Arabia:Riyadh	Saudi Arabia	2019	2019	Missing	Missing	1580	IC2	3	2	NA	NA	ADC-73

M2971410v 1_genomic														
GCA_02412 5815.1_AS M2412581v 1_genomic	CP099795.1	South Korea: Seoul	Korea, Republic of	2011-01-01	2011.0	Missing	Missing	2	IC2	2	1	NA	NA	ADC-30
GCA_03703 9675.1_AS M3703967v 1_genomic	CP146231.1	South Korea	Korea, Republic of	2020-05-06	2020.3	human	human	2	IC2	2	0	NA	NA	ADC-30
GCA_02640 9185.1_AS M2640918v 1_genomic	CP112859.1	South Korea	Korea, Republic of	2021-12-09	2021.9	lab-evolved strain	Lab	2	IC2	2	0	NA	NA	ADC-30
GCA_02412 6315.1_AS M2412631v 1_genomic	CP099793.1	South Korea: Seoul	Korea, Republic of	2011-01-01	2011.0	Missing	Missing	2	IC2	2	1	NA	NA	ADC-30
GCA_01106 7065.1_AS M1106706v 1_genomic	CP049363.1	South Korea: Seoul	Korea, Republic of	2011-01-01	2011.0	urine	Urinary_system	2	IC2	2	2	NA	NA	ADC-30
GCA_02176 4725.1_AS M2176472v 1_genomic	CP091465.1	South Korea: Seoul	Korea, Republic of	2011-01-01	2011.0	urine	Urinary_system	2	IC2	2	1	NA	NA	ADC-30
GCA_02640 9205.1_AS M2640920v 1_genomic	CP112860.1	South Korea	Korea, Republic of	2021-12-09	2021.9	lab-evolved strain	Lab	2	IC2	2	0	NA	NA	ADC-30
GCA_02412 6275.1_AS M2412627v 1_genomic	CP099788.1	South Korea: Seoul	Korea, Republic of	2013-01-01	2013.0	Missing	Missing	2	IC2	2	1	NA	NA	ADC-217
GCA_00208 2745.1_AS M208274v1 _genomic	CP020591.1	South Korea: Seoul	Korea, Republic of	2013-09	2013.7	Endotracheal aspirate	Respiratory_tract	2	IC2	2	0	NA	NA	ADC-217

GCA_041001725.1_ASM4100172v1_genomic	CP163252.1	South Korea: Seoul	Korea, Republic of	2020-09-09	2020.7	blood	Blood	new_alleles	NA	NA	0	NA	NA	ADC-30
GCA_035753605.1_ASM3575360v1_genomic	CP142100.1	South Korea	Korea, Republic of	2020-07-30	2020.6	Missing	Missing	187	IC2	2	0	NA	NA	ADC-56
GCA_035755505.1_ASM3575550v1_genomic	CP142101.1	South Korea	Korea, Republic of	2020-03-09	2020.2	Missing	Missing	2	IC2	2	0	NA	NA	ADC-30
GCA_024137925.1_ASM2413792v1_genomic	CP099969.1	South Korea	Korea, Republic of	Missing	Missing	Missing	Missing	2	IC2	2	1	NA	NA	ADC-30
GCA_024126235.1_ASM2412623v1_genomic	CP099786.1	South Korea: Seoul	Korea, Republic of	2013-01-01	2013.0	Missing	Missing	new_alleles	NA	NA	1	NA	NA	ADC-30
GCA_002082725.1_ASM208272v1_genomic	CP020592.1	South Korea: Seoul	Korea, Republic of	2013-10	2013.8	urine	Urinary_system	2	IC2	2	1	NA	NA	ADC-30
GCA_045689825.1_ASM4568982v1_genomic	CP142652.1	South Korea	Korea, Republic of	2020-03-15	2020.2	human	human	2	IC2	2	8	NA	NA	ADC-30
GCA_045689845.1_ASM4568984v1_genomic	CP142660.1	South Korea	Korea, Republic of	2020-05-18	2020.4	human	human	2	IC2	2	2	NA	NA	ADC-30
GCA_002082685.1_ASM208268v1_genomic	CP020590.1	South Korea	Korea, Republic of	2014-08	2014.6	Pulmonary	Respiratory_tract	2	IC2	2	1	NA	NA	ADC-30
GCA_029774315.1_ASM2977431v1_genomic	CP076736.1	Taiwan	Taiwan	2020-03-03	2020.2	blood	Blood	724	IC2	2	2	OXA-72	NA	ADC-30

GCA_029774355.1_ASM2977435v1_genomic	CP076742.1	Taiwan	Taiwan	2020-07-10	2020.5	pleural effusion	Respiratory_tract	724	IC2	2	2	OXA-72	NA	ADC-30
GCA_029774335.1_ASM2977433v1_genomic	CP076739.1	Taiwan	Taiwan	2020-06-17	2020.5	urine	Urinary_system	724	IC2	2	2	OXA-72	NA	ADC-30
GCA_029774275.1_ASM2977427v1_genomic	CP074698.1	Taiwan	Taiwan	2020	2020	Missing	Missing	724	IC2	2	2	OXA-72	NA	ADC-30
GCA_029814975.1_ASM2981497v1_genomic	CP064194.1	Taiwan	Taiwan	2020	2020	Missing	Missing	724	IC2	2	2	OXA-72	NA	ADC-30
GCA_029774235.1_ASM2977423v1_genomic	CP064203.1	Taiwan	Taiwan	2020	2020	Missing	Missing	724	IC2	2	1	NA	NA	ADC-30
GCA_029774295.1_ASM2977429v1_genomic	CP074695.1	Taiwan	Taiwan	2020	2020	Missing	Missing	724	IC2	2	2	OXA-72	NA	ADC-30
GCA_000302575.1_ASM30257v1_genomic	CP003856.1	Missing	Missing	Missing	Missing	Missing	Missing	2	IC2	2	0	NA	NA	ADC-115
GCA_038441245.1_ASM3844124v1_genomic	CP131901.1	Taiwan	Taiwan	2010	2010	respiratory	Respiratory_tract	2	IC2	2	1	NA	NA	ADC-115
GCA_038441235.1_ASM3844123v1_genomic	CP131898.1	Taiwan	Taiwan	2008	2008	respiratory	Respiratory_tract	2	IC2	2	2	OXA-23	NA	ADC-115
GCA_022548575.1_ASM2254857v1_genomic	CP091358.1	Belgium	Belgium	Missing	Missing	Missing	Missing	2	IC2	2	0	NA	NA	ADC-30

GCA_002762545.2_ASM276254v2_genomic	CP035672.1	India: Vellore	India	2015	2015	blood	Blood	2	IC2	2	3	NA	NA	ADC-30
GCA_046054615.2_ASM4605461v2_genomic	CP181411.1	USA	United States	2024-11-21	2024.9	sputum	Respiratory_tract	2	IC2	4	3	NA	NA	ADC-33
GCF_034331205.1_ASM3433120v2_genomic	NZ_CP143348.1	USA	United States	2023-11-20	2023.9	skin	Skin	2	IC2	4	4	NA	NA	ADC-33
GCA_039654205.1_ASM3965420v1_genomic	CP155476.1	USA	United States	2024-02-20	2024.1	skin	Skin	2	IC2	4	4	NA	NA	ADC-33
GCA_034331205.2_ASM3433120v2_genomic	CP143348.1	USA	United States	2023-11-20	2023.9	skin	Skin	2	IC2	4	4	NA	NA	ADC-33
GCF_037008665.1_ASM3700866v2_genomic	NZ_CP149831.1	USA	United States	2024-02-21	2024.1	sputum	Respiratory_tract	2	IC2	4	4	NA	NA	ADC-33
GCA_037008665.2_ASM3700866v2_genomic	CP149831.1	USA	United States	2024-02-21	2024.1	sputum	Respiratory_tract	2	IC2	4	4	NA	NA	ADC-33
GCF_037751415.1_ASM3775141v2_genomic	NZ_CP155458.1	USA	United States	2024-03-12	2024.2	Wound/Abscess	Skin	2	IC2	4	4	OXA-23	NA	ADC-33
GCA_037751415.2_ASM3775141v2_genomic	CP155458.1	USA	United States	2024-03-12	2024.2	Wound/Abscess	Skin	2	IC2	4	4	OXA-23	NA	ADC-33
GCF_039653385.1_ASM3965338v2_genomic	NZ_CP166162.1	USA	United States	2024-04-26	2024.3	Wound/Abscess	Skin	2	IC2	4	4	NA	NA	ADC-33

GCA_039653385.2_ASM3965338v2_genomic	CP166162.1	USA	United States	2024-04-26	2024.3	Wound/Abscess	Skin	2	IC2	4	4	NA	NA	ADC-33
GCA_041760695.2_2024CK-01357_genomic	CP169597.1	USA	United States	2024-08-16	2024.6	Missing	Missing	2	IC2	4	4	NA	NA	ADC-33
GCA_045689365.1_ASM4568936v1_genomic	CP140438.1	USA	United States	2023-09-28	2023.7	urine	Urinary_system	2	IC2	4	3	NA	NA	ADC-33
GCA_028051765.3_ASM2805176v3_genomic	CP137930.1	USA	United States	2022-12-22	2022.9	Tissue	Skin	2	IC2	4	4	NA	NA	ADC-33
GCF_032634445.1_ASM3263444v3_genomic	NZ_CP140434.1	USA	United States	2023-09-20	2023.7	Wound/Abscess	Skin	2	IC2	4	3	NA	NA	ADC-33
GCA_032634445.3_ASM3263444v3_genomic	CP140434.1	USA	United States	2023-09-20	2023.7	Wound/Abscess	Skin	2	IC2	4	3	NA	NA	ADC-33
GCA_028634015.3_ASM2863401v3_genomic	CP131950.1	USA	United States	2023-01-18	2023.0	Wound/Abscess	Skin	2	IC2	4	3	NA	NA	ADC-33
GCF_028634015.1_ASM2863401v3_genomic	NZ_CP131950.1	USA	United States	2023-01-18	2023.0	Wound/Abscess	Skin	2	IC2	4	3	NA	NA	ADC-33
GCA_028051805.3_ASM2805180v3_genomic	CP137927.1	USA	United States	2022-12-27	2022.9	Tissue	Skin	2	IC2	4	2	NA	NA	ADC-33
GCA_038418365.2_AS	CP157257.1	USA	United States	2024-04-05	2024.3	Missing	Missing	2	IC2	4	2	NA	NA	ADC-33

M3841836v 2_genomic														
GCF_03841 8365.1_AS M3841836v 2_genomic	NZ_CP1572 57.1	USA	United States	2024-04-05	2024.3	Missing	Missing	2	IC2	4	2	NA	NA	ADC-33
GCA_04089 8785.2_AS M4089878v 2_genomic	CP168455.1	USA	United States	2024-07-05	2024.5	sputum	Respiratory_tract	2	IC2	4	1	NA	NA	ADC-33
GCA_04453 7525.2_AS M4453752v 2_genomic	CP180034.1	USA	United States	2024-10-16	2024.8	Wound/Abscess	Skin	2	IC2	4	1	NA	NA	ADC-33
GCA_04609 7205.1_HU MC1_Hybrid _Assembly_ genomic	CP175646.1	USA: California	United States	2009	2009	Missing	Missing	2	IC2	4	2	NA	NA	ADC-33
GCA_03016 8245.1_AS M3016824v 1_genomic	CP096818.1	USA:Georga, Atlanta	United States	2012/2015	2014	blood	Blood	2	IC2	4	2	NA	NA	ADC-33
GCA_04609 7175.1_UM B001_Hybri d_Assembly _genomic	CP175656.1	USA: Maryland	United States	2008	2008	blood	Blood	2	IC2	4	2	APH(3')-VIa	NA	ADC-33
GCA_00803 3255.2_AS M803325v2 _genomic	CP042841.1	USA:DC	United States	2008	2008	sputum	Respiratory_tract	2	IC2	4	2	NA	NA	ADC-33
GCA_02572 2955.1_AS M2572295v 1_genomic	CP106988.1	Missing	Missing	Missing	Missing	sputum	Respiratory_tract	2	IC2	4	3	NA	NA	ADC-33
GCA_00166 8465.2_AS M166846v2 _genomic	CP035049.1	Missing	Missing	2015-05-24	2015.4	Missing	Missing	2	IC2	4	1	NA	NA	ADC-33

GCA_014882805.2_ASM1488280v2_genomic	CP081144.1	USA	United States	2018	2018	Respiratory_tract	Respiratory_tract	2	IC2	4	3	OXA-23	NA	ADC-33
GCA_014881055.2_ASM1488105v2_genomic	CP081139.1	USA	United States	2018	2018	Blood	Blood	2	IC2	4	4	OXA-23	NA	ADC-224
GCA_042145195.1_Lv646_genomic	CP169826.1	USA	United States	2020	2020	respiratory	Respiratory_tract	195	IC2	4	1	NA	NA	ADC-227
GCA_042145845.1_Lv647_genomic	CP169828.1	USA	United States	2020	2020	respiratory	Respiratory_tract	195	IC2	4	3	OXA-23	NA	ADC-227
GCA_001674475.2_ASM167447v2_genomic	CP035051.1	Missing	Missing	2015-02-26	2015.2	Missing	Missing	2	IC2	4	2	OXA-23 # APH(3')-VIa	NA	ADC-33
GCA_001674505.2_ASM167450v2_genomic	CP035043.1	Missing	Missing	2015-10-30	2015.8	Missing	Missing	2	IC2	4	1	APH(3')-VIa	NA	ADC-33
GCA_001669145.2_ASM166914v2_genomic	CP035045.1	Missing	Missing	2015-11-08	2015.9	Missing	Missing	2	IC2	4	3	OXA-23 # APH(3')-VIa	NA	ADC-33
GCA_022467905.1_ASM2246790v1_genomic	CP091343.1	Belgium	Belgium	Missing	Missing	Missing	Missing	2	IC2	4	0	NA	NA	ADC-33
GCA_022468795.1_ASM2246879v1_genomic	CP091353.1	Belgium	Belgium	Missing	Missing	Missing	Missing	2	IC2	4	0	NA	NA	ADC-33
GCA_030027985.1_ASM3002798v1_genomic	CP125223.1	USA: Boston	United States	2011	2011	peripheral blood	Blood	2	IC2	4	1	NA	NA	ADC-33
GCA_030028005.1_AS	CP125225.1	USA: Boston	United States	2010	2010	hip wound	Skin	2	IC2	4	1	NA	NA	ADC-33

M3002800v1_genomic														
GCA_046097185.1_NIH1_Hybrid_Assembly_genomic	CP175653.1	USA: Maryland	United States	2007	2007	Missing	Missing	2	IC2	4	2	NA	NA	ADC-33
GCA_046097165.1_LUH5537_Hybrid_Assembly_genomic	CP175648.1	Netherlands: Leiden	Netherlands	Missing	Missing	Missing	Missing	2	IC2	4	2	APH(3')-Ia	NA	ADC-25
GCA_029814355.1_ASM2981435v1_genomic	CP122363.1	Russia:Moscow	Russian Federation	2016-01-28	2016.1	sputum	Respiratory_tract	2	IC2	4	1	NA	NA	ADC-11
GCA_017584105.1_ASM1758410v1_genomic	CP071919.1	Russia:Moscow	Russian Federation	2017	2017	blood	Blood	45	IC2	4	1	OXA-72 # OXA-72	NA	ADC-11
GCA_043045365.1_ASM4304536v1_genomic	CP134560.1	China: Henan	China	2010	2010	sputum	Respiratory_tract	2	IC2	2	0	NA	NA	ADC-25
GCA_041154785.1_ASM4115478v1_genomic	AP031577.1	Nepal:Kathmandu	Nepal	2019	2019	Missing	Missing	2	IC2	2	0	NA	NA	ADC-30
GCA_005280715.1_ASM528071v1_genomic	CP040087.1	India	India	2018	2018	blood	Blood	2	IC2	2	1	NA	NA	ADC-30
GCA_003627485.2_ASM362748v2_genomic	CP035930.1	India: Vellore	India	2017	2017	blood	Blood	NA	NA	NA	2	NA	NA	ADC-30
GCA_041154795.1_ASM4115479v1_genomic	AP031578.1	Nepal:Kathmandu	Nepal	2019	2019	Missing	Missing	2	IC2	2	0	NA	NA	ADC-30

GCA_043053445.1_ASM4305344v1_genomic	CP134591.1	China: Guangdong	China	2010	2010	sputum	Respiratory_tract	2	IC2	3	1	NA	NA	ADC-73
GCA_001573065.1_ASM157306v1_genomic	CP014538.1	China: Guangdong	China	2009	2009	Missing	Missing	2	IC2	3	0	NA	NA	ADC-73
GCA_014267385.1_ASM1426738v1_genomic	CP060285.1	China:Changsha	China	2018-05-11T10:12:05Z	2018.4	blood	Blood	2	IC2	3	0	NA	NA	ADC-73
GCA_016865465.1_ASM1686546v1_genomic	AP024415.1	Cambodia:Phnom Penh	Cambodia	2017	2017	Missing	Missing	571	IC2	2	0	NA	NA	ADC-82
GCA_043051495.1_ASM4305149v1_genomic	CP134580.1	China: Heilongjiang	China	2010	2010	sputum	Respiratory_tract	2	IC2	2	2	NA	NA	ADC-30
GCA_042209675.2_ASM4220967v2_genomic	CP174050.1	USA	United States	2024-08-23	2024.6	skin	Skin	2	IC2	2	1	NA	NA	ADC-30
GCA_009455505.1_ASM945550v1_genomic	CP045528.1	India	India	Missing	Missing	Missing	Missing	2	IC2	2	0	NA	NA	ADC-30
GCA_043043455.1_ASM4304345v1_genomic	CP134553.1	China: Shanxi	China	2010	2010	sputum	Respiratory_tract	104	IC2	2	1	OXA-23	NA	ADC-30
GCA_022468835.2_ASM2246883v2_genomic	CP091354.1	Belgium	Belgium	2017	2017	Missing	Missing	636	Not IC	NA	1	OXA-72	NA	ADC-74
GCA_022468855.2_ASM2246885v2_genomic	CP091355.1	Belgium	Belgium	2017	2017	Missing	Missing	636	Not IC	NA	1	OXA-72	NA	ADC-74

GCA_022460745.2_ASM2246074v2_genomic	CP091376.1	Belgium	Belgium	2014	2014	Missing	Missing	636	Not IC	NA	1	OXA-72	NA	ADC-74
GCA_022460875.2_ASM2246087v2_genomic	CP091377.1	Belgium	Belgium	2014	2014	Missing	Missing	636	Not IC	NA	1	OXA-72	NA	ADC-74
GCA_022459115.2_ASM2245911v2_genomic	CP091362.1	Belgium	Belgium	2017	2017	Missing	Missing	636	Not IC	NA	1	OXA-72	NA	ADC-74
GCA_022460435.2_ASM2246043v2_genomic	CP091374.1	Belgium	Belgium	2014	2014	Missing	Missing	636	Not IC	NA	1	OXA-72	NA	ADC-74
GCA_024749765.1_ASM2474976v1_genomic	CP087354.1	Germany	Germany	Missing	Missing	Missing	Missing	636	Not IC	NA	2	APH(3')-VIa # OXA-72	NA	ADC-74
GCA_024749745.1_ASM2474974v1_genomic	CP087351.1	Germany	Germany	Missing	Missing	Missing	Missing	636	Not IC	NA	2	APH(3')-VIa # OXA-72	NA	ADC-74
GCA_024749785.1_ASM2474978v1_genomic	CP087374.1	Germany	Germany	Missing	Missing	Missing	Missing	636	Not IC	NA	2	APH(3')-VIa # OXA-72	NA	ADC-74
GCA_024749725.1_ASM2474972v1_genomic	CP087370.1	Germany	Germany	Missing	Missing	Missing	Missing	636	Not IC	NA	3	APH(3')-VIa # OXA-72 # AAC(3)-Ia # aadA # qacEdelta1 # sul1 # APH(3')-Ia	NA	ADC-74
GCA_024749705.1_ASM2474970v1_genomic	CP087365.1	Germany	Germany	Missing	Missing	Missing	Missing	636	Not IC	NA	4	APH(3')-VIa # OXA-72	NA	ADC-74

GCA_024749665.1_ASM2474966v1_genomic	CP087344.1	Germany	Germany	Missing	Missing	Missing	Missing	636	Not IC	NA	3	APH(3')-VIa # OXA-72	NA	ADC-74
GCA_042140585.1_Hv770_genomic	CP169791.1	France: Paris	France	2022	2022	respiratory	Respiratory_tract	636	Not IC	NA	3	OXA-72	NA	ADC-276
GCA_019457735.1_ASM1945773v1_genomic	CP078099.1	France	France	2012-02	2012.1	Missing	Missing	2	IC2	2	1	NA	NA	ADC-30
GCA_016903135.1_ASM1690313v1_genomic	CP069840.1	USA:MD	United States	Missing	Missing	Missing	Missing	2	IC2	1	2	APH(3')-VIa # OXA-23	NA	ADC-25
GCA_038441195.1_ASM3844119v1_genomic	CP131885.1	Taiwan	Taiwan	2008	2008	Abs/pus/wd	Skin	195	IC2	2	3	OXA-72	NA	ADC-30
GCA_043052795.1_ASM4305279v1_genomic	CP134589.1	China: Guangdong	China	2010	2010	Bronchoscopic brushing	Respiratory_tract	215	Not IC	NA	0	NA	NA	ADC-30
GCA_001573105.1_ASM157310v1_genomic	CP014540.1	China: Guangdong	China	2010	2010	sputum	Respiratory_tract	215	Not IC	NA	0	NA	NA	ADC-30
GCA_022467435.1_ASM2246743v1_genomic	CP091338.1	Belgium	Belgium	Missing	Missing	Missing	Missing	215	Not IC	NA	0	NA	NA	ADC-30
GCA_024749585.1_ASM2474958v1_genomic	CP087362.1	Germany	Germany	Missing	Missing	Missing	Missing	867	Not IC	NA	2	NA	NA	ADC-312
GCA_013377175.1_ASM1337717v1_genomic	CP046898.1	China:Guangdong	China	2010-10	2010.8	secretion	Secretion	108	Not IC	NA	3	OXA-24 # TEM-1 # AAC(3)-IIe # AAC(6')-Ian #	NA	ADC-236

												APH(3'')-Ib # APH(6)-Id # tet(B) # sul2 # tetR		
GCA_043052145.1_ASM4305214v1_genomic	CP134583.1	China: Guangdong	China	2010	2010	secretion	Secretion	108	Not IC	NA	2	APH(3'')-Ib # APH(6)-Id # tet(B) # sul2 # TEM-1 # AAC(3)-IIe # AAC(6')-Ian # tetR	NA	ADC-236
GCA_001721705.1_ASM172170v1_genomic	CP012587.1	Australia	Australia	2010	2010	Missing	Missing	1546	Not IC	NA	0	NA	NA	ADC-328
GCA_028583465.1_ASM2858346v1_genomic	CP113077.1	Poland	Poland	2013	2013	Nestling, choana	White_stork	690	Not IC	NA	0	NA	NA	ADC-312
GCA_032822615.1_ASM3282261v1_genomic	CP127906.1	Australia: South Australia	Australia	2019	2019	lake	Water	350	Not IC	NA	0	NA	NA	ADC-332
GCA_040822435.1_ASM4082243v1_genomic	CP161999.1	Singapore	Singapore	2009-11-17	2009.9	Missing	Missing	150	Not IC	NA	0	NA	NA	ADC-163
GCA_036584025.1_ASM3658402v1_genomic	CP144465.1	Missing	Missing	2017-04-17	2017.3	Missing	Missing	150	Not IC	NA	3	sul2 # tet(B) # APH(6)-Id # APH(3'')-Ib # tetR	NA	ADC-163
GCA_042478285.1_ASM4247828v1_genomic	CP133052.1	China	China	2010	2010	sputum	Respiratory_tract	150	Not IC	NA	0	NA	NA	ADC-163

GCA_009740145.1_ASM974014v1_genomic	CP046536.1	China:Nanjing	China	2018-07-01	2018.5	cucumber rhizosphere	Soil	150	Not IC	NA	1	NA	NA	ADC-163
GCA_041930545.1_ASM4193054v1_genomic	CP169375.1	China:nanjing	China	2023-03-01	2023.2	rhizosphere soil	Soil	150	Not IC	NA	1	NA	NA	ADC-163
GCA_900093475.1_36734_D01_2_genomic	LT605059.1	Missing	Missing	Missing	Missing	Missing	Missing	494	Not IC	NA	1	sul2	NA	ADC-106
GCA_004354125.1_ASM435412v1_genomic	CP037869.1	Canada: Ontario	Canada	2013	2013	Drainage basin water	Water	1039	Not IC	NA	0	NA	NA	ADC-158
GCA_035666135.1_ASM3566613v1_genomic	CP142019.1	China:Nanjing	China	2023-10	2023.8	Missing	Missing	new_ST	NA	NA	0	NA	NA	ADC-103
GCA_020985285.1_ASM2098528v1_genomic	CP087594.1	China: Shanghai	China	2021-07-11	2021.5	Andrias davidianus	Salamander	40	Not IC	NA	2	NA	NA	ADC-155
GCA_043056915.1_ASM4305691v1_genomic	CP134601.1	China: Guangdong	China	2010	2010	sputum	Respiratory_tract	40	Not IC	NA	0	NA	NA	ADC-155
GCA_027239755.1_ASM2723975v1_genomic	CP102762.1	China:Beijing	China	2020-07	2020.5	blood	Blood	40	Not IC	NA	3	aadT # mphE # msrE # APH(6)-Id # APH(3'')-Ib # sul2 # AAC(3)-IId # OXA-58 # OXA-58 # OXA-58 # OXA-58	NA	ADC-155

												# OXA-58 # OXA-58		
GCA_022605145.1_ASM2260514v1_genomic	CP091173.1	China: Xi'an	China	2013	2013	sputum	Respiratory_tract	40	Not IC	NA	0	NA	NA	ADC-155
GCA_026194635.2_ASM2619463v2_genomic	CP113442.1	USA: Oregon	United States	2019-09-10	2019.7	Draining-Matting Conveyor	Draining_Matting_Conveyor	40	Not IC	NA	11	NA	NA	ADC-155
GCA_043451925.1_ASM4345192v1_genomic	CP134586.1	China: Beijing	China	2010	2010	sputum	Respiratory_tract	40	Not IC	NA	2	OXA-23	NA	ADC-155
GCA_043572315.1_T14_genomic	CP171396.1	Turkey: Kayseri	Türkiye	2023-08-15	2023.6	Broncho alveoler lavaj	Respiratory_tract	new_ST	NA	NA	1	NA	NA	ADC-270
GCA_043572945.1_T24_genomic	CP171398.1	Turkey: Kayseri	Türkiye	2023-08-15	2023.6	blood	Blood	new_ST	NA	NA	1	NA	NA	ADC-270
GCA_000505685.2_ASM50568v2_genomic	CP006768.1	Missing	Missing	Missing	Missing	Missing	Missing	639	Not IC	NA	2	APH(3')-VIa # NDM-1 # sul2 # tet(B) # tetR	NA	ADC-165
GCF_000505685.1_ASM50568v2_genomic	NC_023028.1	Missing	Missing	Missing	Missing	Clinical_sample	Clinical_sample	639	Not IC	NA	2	APH(3')-VIa # NDM-1 # sul2 # tet(B) # tetR	NA	ADC-165
GCA_045345925.1_ASM4534592v1_genomic	CP150097.1	China: Zhengzhou, Henan Province	China	2021-12-10	2021.9	soil	Soil	new_ST	NA	NA	2	NA	NA	ADC-165
GCA_003812385.1_ASM381238v1_genomic	CP033768.1	Missing	Missing	2016-04-15	2016.3	sputum	Respiratory_tract	57	Not IC	NA	2	ANT(2'')-Ia	NA	ADC-247

GCA_019903155.1_ASM1990315v1_genomic	CP059542.1	Finland	Finland	Missing	Missing	paper mill kaolin	Paper_production	648	Not IC	NA	3	NA	NA	ADC-105
GCA_001077555.2_ASM107755v2_genomic	CP021342.1	India: Vellore	India	2014-03-15	2014.2	blood	Blood	1545	Not IC	NA	0	NA	NA	ADC-247
GCA_003522665.1_ASM352266v1_genomic	CP023020.1	Mexico: Guadalajara	Mexico	2013-08	2013.6	blood	Blood	422	IC5	NA	1	OXA-72	NA	ADC-259
GCA_002947845.1_ASM294784v1_genomic	CP026711.1	Missing	Missing	Missing	Missing	Missing	Missing	79	IC5	NA	1	OXA-23	NA	ADC-114
GCA_042147895.1_Rp426_genomic	CP169839.1	Missing	Missing	Missing	Missing	Missing	Missing	298	IC5	NA	5	OXA-24	NA	ADC-214
GCA_042144055.1_Lv419_genomic	CP169819.1	USA	United States	2019	2019	Urinary source	Urinary_system	79	IC5	NA	3	NA	NA	ADC-214
GCA_050320305.1_Up438_genomic	CP190375.1	USA	United States	2019	2019	Respiratory/Endotracheal	Respiratory_tract	79	IC5	NA	7	NA	NA	ADC-336
GCA_050320255.1_Hv637_genomic	CP190359.1	USA	United States	2020	2020	respiratory	Respiratory_tract	79	IC5	NA	2	NA	NA	ADC-214
GCA_003010675.1_ASM301067v1_genomic	CP027611.1	Missing	Missing	Missing	Missing	Missing	Missing	79	IC5	NA	1	NA	NA	ADC-214
GCA_042148545.1_Rp428_genomic	CP169842.1	USA	United States	2019	2019	urine	Urinary_system	79	IC5	NA	5	OXA-23 # sul2 # APH(3'')-Ib # APH(6)-Id # mphE # msrE # AAC(3)-IId	NA	ADC-259

GCA_000746645.1_ASM74664v1_genomic	CP009257.1	Canada: Winnipeg	Canada	2010-01-01	2010.0	hospital	Hospital_enviroment	79	IC5	NA	0	NA	NA	ADC-5
GCA_001922425.2_ASM192242v2_genomic	CP018861.2	Mexico	Mexico	2012	2012	Bronchial	Respiratory_tract	156	IC5	NA	1	NA	NA	ADC-214
GCA_003345235.1_ASM334523v1_genomic	CP026338.1	Mexico: Mexico City	Mexico	2015-01-11	2015.0	feces	Digestive_system	156	IC5	NA	2	NA	NA	ADC-214
GCA_050269755.1_ASM5026975v1_genomic	CP189874.1	Venezuela: Caracas	Venezuela	2015	2015	skin	Skin	79	IC5	NA	3	OXA-23 # qacJ	NA	ADC-5
GCA_000761175.1_ASM76117v1_genomic	CP009534.1	Missing	Missing	Missing	Missing	Missing	Missing	79	IC5	NA	0	NA	NA	ADC-5
GCA_001628795.1_ASM162879v1_genomic	CP014266.1	Spain	Spain	2010	2010	biological sample	Missing	79	IC5	NA	0	NA	NA	ADC-5
GCA_001896005.1_ASM189600v1_genomic	CP018254.1	Mexico	Mexico	2009-07-01	2009.5	Small Colon	Digestive_system	79	IC5	NA	1	OXA-235	NA	ADC-5
GCA_003812485.1_ASM381248v1_genomic	CP033869.1	Brazil: Salvador	Brazil	2008	2008	cerebrospinal fluid	CNS	79	IC5	NA	3	MCR-4.3	NA	ADC-182
GCA_024139035.1_ASM2413903v1_genomic	CP076812.1	Chile:Santiago	Chile	2011	2011	blood culture	Blood	79	IC5	NA	1	NA	NA	ADC-263
GCA_050030185.1_ASM5003018v1_genomic	CP179694.1	Paraguay: Itaugua	Paraguay	2024-01-07	2024.0	urine	Urinary_system	79	IC5	NA	1	NA	NA	ADC-5

GCA_025369955.1_ASM2536995v1_genomic	CP104448.1	USA	United States	2021-08-23	2021.6	sputum	Respiratory_tract	422	IC5	NA	0	NA	NA	ADC-259
GCA_025258705.1_ASM2525870v1_genomic	CP104335.1	USA	United States	2021-08-30	2021.7	blood	Blood	422	IC5	NA	3	NA	NA	ADC-259
GCA_025258745.1_ASM2525874v1_genomic	CP104340.1	USA	United States	2021-08-30	2021.7	Wound/Abscess	Skin	422	IC5	NA	1	NA	NA	ADC-259
GCA_025258805.1_ASM2525880v1_genomic	CP104350.1	USA	United States	2021-08-10	2021.6	sputum	Respiratory_tract	422	IC5	NA	1	NA	NA	ADC-259
GCA_025258765.1_ASM2525876v1_genomic	CP104342.1	USA	United States	2021-08-02	2021.6	Wound/Abscess	Skin	422	IC5	NA	1	OXA-72	NA	ADC-259
GCA_025258845.1_ASM2525884v1_genomic	CP104351.1	USA	United States	2021-08-09	2021.6	Wound/Abscess	Skin	422	IC5	NA	1	OXA-72	NA	ADC-259
GCA_025258785.1_ASM2525878v1_genomic	CP104347.1	USA	United States	2021-08-09	2021.6	Wound/Abscess	Skin	422	IC5	NA	1	NA	NA	ADC-259
GCA_009497995.1_ASM949799v1_genomic	CP033243.1	Mexico: Monterrey, Nuevo Leon	Mexico	2007-07-16	2007.5	Tissue	Skin	422	IC5	NA	2	NA	NA	ADC-183
GCA_001636235.1_ASM163623v1_genomic	CP015364.1	Mexico: Monterrey NL	Mexico	2008-08-05	2008.6	Bronchial fluid	Respiratory_tract	422	IC5	NA	2	OXA-58	NA	ADC-183
GCA_004354145.1_ASM435414v1_genomic	CP037872.1	Canada: Ontario	Canada	2013	2013	Drainage basin water	Water	2250	Not IC	NA	0	NA	NA	ADC-274

GCA_040822125.1_ASM4082212v1_genomic	CP161986.1	Singapore	Singapore	2006-03-11	2006.2	Missing	Missing	10	IC8	NA	2	sul2 # tet(B) # tetR	NA	ADC-76
GCA_040357515.1_ASM4035751v1_genomic	CP128372.1	China: Zhoushan	China	2021-08-18	2021.6	Missing	Missing	10	IC8	NA	2	tet(B) # sul2 # tetR	NA	ADC-76
GCA_045037785.1_ASM4503778v1_genomic	CP173428.1	South Korea	Korea, Republic of	2018-11	2018.8	blood	Blood	10	IC8	NA	1	NA	NA	ADC-76
GCA_045278625.1_ASM4527862v1_genomic	CP137075.1	South Korea: Republic of	Korea, Republic of	2018-01-15T11:15Z	2018.0	Seoul National University Bundang Hospital Intensive Care Unit	Hospital_enviroment	10	IC8	NA	1	NA	NA	ADC-76
GCA_002082805.1_ASM208280v1_genomic	CP020598.1	South Korea: Jeonju	Korea, Republic of	2013-10	2013.8	sputum	Respiratory_tract	10	IC8	NA	0	NA	NA	ADC-76
GCA_040822395.1_ASM4082239v1_genomic	CP161989.1	Singapore	Singapore	2008-07-11	2008.5	Missing	Missing	10	IC8	NA	2	sul2 # tet(B) # tetR	NA	ADC-76
GCA_043040895.1_ASM4304089v1_genomic	CP134548.1	China: Jiangsu	China	2010	2010	sputum	Respiratory_tract	10	IC8	NA	0	NA	NA	ADC-76
GCA_012935105.1_ASM1293510v1_genomic	CP050425.1	India	India	2019	2019	BAL	Respiratory_tract	10	IC8	NA	6	mphE # msrE # armA # sul1 # cmlA5 # arr-2 # sul2 # APH(6)-Id # APH(3'')-	NA	ADC-76

												Ib # NDM-1 # sul2 # tet(B) # tetR		
GCA_012935085.1_ASM1293508v1_genomic	CP050415.1	India	India	2019	2019	Pus	Skin	10	IC8	NA	5	mphE # msrE # armA # sul1 # cmlA5 # arr-2 # sul2 # APH(6)-Id # APH(3'')-Ib # NDM-1 # sul2 # tet(B) # tetR	NA	ADC-76
GCA_042142205.1_HvAb04_genomic	CP169805.1	Missing	Missing	Missing	Missing	Missing	Missing	10	IC8	NA	3	sul1 # PER-7 # sul1 # cmlA5 # arr-2 # sul2 # APH(6)-Id # APH(3'')-Ib # sul2 # tet(B) # mphE # msrE # armA # OXA-23 # tetR	NA	ADC-76
GCA_001077655.1_ASM107765v1_genomic	CP012006.1	Canada: Edmonton	Canada	2012	2012	blood	Blood	10	IC8	NA	2	APH(3'')-Ib # APH(6)-Id # arr-2 # cmlA5 # qacEdelta1 # sul1 # PER-7 #	NA	ADC-76

												sul1 # armA # msrE # mphE # tet(B) # sul2 # OXA-23 # tetR		
GCA_001908295.1_ASM190829v1_genomic	CP018677.1	USA	United States	1997-01-02	1997.0	Missing	Missing	10	IC8	NA	2	ANT(2'')-Ia	NA	ADC-76
GCA_000786735.1_ASM78673v1_genomic	CP007712.1	USA: Los Angeles County, California	United States	1997-01-02	1997.0	Missing	Missing	10	IC8	NA	2	ANT(2'')-Ia	NA	ADC-76
GCA_012935125.1_ASM1293512v1_genomic	CP050432.1	India	India	2019	2019	BAL	Respiratory_tract	10	IC8	NA	3	mphE # msrE # armA # sul1 # PER-7 # sul1 # cmlA5 # arr-2 # sul2 # APH(6)-Id # APH(3'')-Ib # sul2 # tet(B) # tetR	NA	ADC-76
GCA_041154835.1_ASM4115483v1_genomic	AP031582.1	Nepal:Kathmandu	Nepal	2019	2019	Missing	Missing	10	IC8	NA	0	NA	NA	ADC-76
GCA_022459075.1_ASM2245907v1_genomic	CP091360.1	Belgium	Belgium	Missing	Missing	Missing	Missing	10	IC8	NA	0	NA	NA	ADC-76
GCA_036701095.1_AS	CP144907.1	Missing	Missing	2019-03-06	2019.2	Missing	Missing	10	IC8	NA	2	NA	NA	ADC-76

M3670109v1_genomic														
GCA_040822385.1_ASM4082238v1_genomic	CP161995.1	Singapore	Singapore	2008-10-31	2008.8	Missing	Missing	575	IC8	NA	3	tet(B) # sul2 # msrE # mphE # tetR	NA	ADC-76
GCA_003522705.1_ASM352270v1_genomic	CP023022.1	Mexico: Acapulco, Guerrero	Mexico	2012-08	2012.6	Bronchial fluid	Respiratory_tract	10	IC8	NA	3	tet(B) # sul2 # tetR	NA	ADC-76
GCA_005280415.1_ASM528041v1_genomic	CP040056.1	India	India	2018	2018	blood	Blood	575	IC8	NA	1	CARB-58 # qacEdelta1 # PER-7 # APH(3')-VIa # aadT	NA	ADC-76
GCA_019264825.1_ASM1926482v1_genomic	CP078527.1	Thailand	Thailand	Missing	Missing	Missing	Missing	1435	Not IC	NA	0	NA	NA	ADC-76
GCA_004758865.1_ASM475886v1_genomic	CP038500.1	India	India	2018	2018	blood	Blood	10	IC8	NA	3	tet(39) # AAC(3)-IId # msrE # mphE # OXA-58 # PER-7 # sul1 # aadT	NA	ADC-76
GCA_005280395.1_ASM528039v1_genomic	CP040053.1	India	India	2018	2018	blood	Blood	1512	IC8	NA	2	APH(3'')-Ib # APH(6)-Id # sul2 # arr-2 # cmlA5 # sul1 # PER-7 # armA # msrE # mphE # tet(B) #	NA	ADC-331

												sul2 # tetR		
GCA_034081005.1_ASM3408100v1_genomic	CP139204.1	China: Zhengzhou	China	2022-03-10	2022.2	throat swab	Respiratory_tract	1153	Not IC	NA	3	NA	NA	ADC-76
GCA_000419405.1_ASM41940v1_genomic	CP003847.1	Missing	Missing	Missing	Missing	Missing	Missing	23	IC8	NA	1	APH(3')-VIa # AAC(3)-IId # OXA-58	NA	ADC-76
GCA_023981045.1_ASM2398104v1_genomic	CP045645.1	China:Shijiazhuang	China	2010-08-31	2010.7	sputum	Respiratory_tract	256	IC8	NA	0	NA	NA	ADC-76
GCA_043046095.1_ASM4304609v1_genomic	CP134562.1	China: Henan	China	2010	2010	sputum	Respiratory_tract	23	IC8	NA	1	AAC(3)-IId # APH(3')-VIa # OXA-58	NA	ADC-76
GCA_001578145.1_ASM157814v1_genomic	CP014528.1	China:Zhejiang	China	2010	2010	sputum	Respiratory_tract	23	IC8	NA	0	NA	NA	ADC-76
GCA_012974585.1_ASM1297458v1_genomic	CP048827.1	China:Guangdong province	China	2017-12	2017.9	trachea	Respiratory_tract	23	IC8	NA	1	tet(M) # aadT # OXA-58 # AAC(3)-IId # APH(3')-VIa # NDM-1 # floR # sul2 # tet(X6)	NA	ADC-76
GCA_036689575.1_ASM3668957v1_genomic	CP144757.1	Missing	Missing	2019-03-07	2019.2	Missing	Missing	10	IC8	NA	0	NA	NA	ADC-158
GCA_004354165.1_ASM435416v1_genomic	CP037871.1	Canada: Ontario	Canada	2013	2013	Drainage basin water	Water	2251	Not IC	NA	0	NA	NA	ADC-356

GCA_021729465.1_ASM2172946v1_genomic	CP054560.1	China: Jiangsu Province, Yancheng City	China	2019-01-20	2019.1	stool	Digestive_system	1093	Not IC	NA	1	NA	NA	ADC-356
GCA_013394265.1_ASM1339426v1_genomic	CP044517.1	China:Guangdong	China	2017-06	2017.4	feces	Digestive_system	1093	Not IC	NA	1	tet(39)	NA	ADC-356
GCA_019264845.1_ASM1926484v1_genomic	CP078528.1	Thailand: Narathiwat	Thailand	Missing	Missing	food	food	2207	Not IC	NA	0	NA	NA	ADC-76
GCA_050408015.1_ASM5040801v1_genomic	CP191205.1	China	China	2024	2024	sputum	Respiratory_tract	392	Not IC	NA	0	NA	NA	ADC-271
GCA_020810655.1_ASM2081065v1_genomic	CP085788.1	Missing	Missing	Missing	Missing	Missing	Missing	729	Not IC	NA	2	NA	NA	ADC-68
GCA_020271605.1_ASM2027160v1_genomic	CP084297.1	China: Nantong, jiangsu province	China	2020-05	2020.3	a feces sample of chicken origin	Chicken	2253	Not IC	NA	2	OXA-58 # aadT # floR # tet(M) # AAC(3)-IVa # APH(4)-Ia # sul2 # tet(X6) # dfrA1 # SAT-2 # aadA # mphE # msrE # ErmB	NA	ADC-166
GCA_033190095.1_ASM3319009v1_genomic	CP136178.1	France: Marcy l'etoile	France	2021-09-22	2021.7	oral swab	Respiratory_tract	1384	Not IC	NA	0	NA	NA	ADC-346

GCA_049177255.1_ASM4917725v1_genomic	CP185772.1	India: Broadway, Kochi, Kerala	India	2023-09-03	2023.7	shrimp	shrimp	1422	Not IC	NA	0	NA	NA	ADC-57
GCA_004564115.1_ASM456411v1_genomic	CP038258.1	Czech Republic	Czech Republic	2018	2018	Missing	Missing	345	Not IC	NA	3	MCR-4.3	NA	ADC-6
GCA_004564095.1_ASM456409v1_genomic	CP038262.1	Czech Republic	Czech Republic	2018	2018	Missing	Missing	345	Not IC	NA	3	MCR-4.3	NA	ADC-6
GCA_041501475.1_Acinetobacter_baumannii_strain_G6290164_partial_genome_sequence_genomic	CP167770.1	Brazil: Sao Paulo	Brazil	2015-10	2015.8	rectal swab	Digestive_system	329	Not IC	NA	6	NDM-1 # APH(3')-VIa	NA	ADC-6
GCA_024139015.1_ASM2413901v1_genomic	CP076807.1	Chile:Santiago	Chile	2010	2010	peritoneal abcess	Abdomen	109	Not IC	NA	4	sul2 # AAC(3)-IIe # APH(3')-VIa # APH(3'')-Ib # OXA-58	NA	ADC-263 # ADC-263
GCA_004194555.1_ASM419455v1_genomic	CP028138.1	USA: PA, Chesnut Hill	United States	1943	1943	aerobic microbial decomposition of whole and defoliated guayule (retting)	guayule	1197	Not IC	NA	1	NA	NA	ADC-154
GCA_013787185.1_ASM1378718v1_genomic	CP059386.1	Russia: Moscow	Russian Federation	2013-01-01	2013.0	respiratory tract	Respiratory_tract	203	Not IC	NA	4	sul2	NA	ADC-154

GCA_008802935.1_ASM880293v1_genomic	CP044356.1	Cambodia: Battambang	Cambodia	2016-01-13	2016.0	oral cavity	Respiratory_tract	203	Not IC	NA	2	NA	NA	ADC-6
GCA_043048185.1_ASM4304818v1_genomic	CP134566.1	China: Shaanxi	China	2010	2010	secretion	Secretion	239	Not IC	NA	3	APH(3')-VIa	NA	ADC-165
GCA_000372585.2_ASM37258v2_genomic	CP012035.1	Malaysia: Selangor	Malaysia	2012-05	2012.3	blood	Blood	239	Not IC	NA	0	NA	NA	ADC-165
GCA_013416275.1_ASM1341627v1_genomic	CP053218.1	Tanzania: Dar es Salaam	Tanzania, United Republic of	2017-11	2017.8	Missing	Missing	239	Not IC	NA	3	floR # sul2 # AAC(3)-IId # CARB-16 # APH(3')-VIa # NDM-1	NA	ADC-165
GCA_002902885.1_ASM290288v1_genomic	CP026125.1	USA	United States	2016	2016	Missing	Missing	1543	Not IC	NA	4	NDM-1 # APH(3')-VIa	NA	ADC-165
GCA_042151695.1_Rp779_genomic	CP169866.1	USA: St. Louis	United States	2018	2018	respiratory	Respiratory_tract	new_ST	NA	NA	2	NA	NA	ADC-154
GCA_029674825.1_ASM2967482v1_genomic	CP121370.1	China:GuangZhou	China	2020-05-10	2020.4	wastewater	Water	331	Not IC	NA	4	NA	NA	ADC-154
GCA_029674845.1_ASM2967484v1_genomic	CP121365.1	China:GuangZhou	China	2020-05-10	2020.4	wastewater	Water	331	Not IC	NA	4	NA	NA	ADC-154
GCA_029674805.1_ASM2967480v1_genomic	CP121375.1	China:GuangZhou	China	2020-05-10	2020.4	wastewater	Water	331	Not IC	NA	4	NA	NA	ADC-154
GCA_024749505.1_AS	CP087300.1	Germany	Germany	Missing	Missing	Missing	Missing	58	Not IC	NA	3	APH(3')-VIa # sul1	NA	ADC-154

M2474950v 1_genomic												# ANT(2'')-Ia # GIM-1		
GCA_015571935.1_ASM1557193v 1_genomic	CP062919.1	Canada: Quebec	Canada	2018-01-17	2018.0	swab	Hospital_enviroment	2252	Not IC	NA	4	NA	NA	ADC-99
GCA_047772455.1_ASM4777245v 1_genomic	CP179883.1	USA	United States	2011	2011	Missing	Missing	15	IC4	NA	3	sul2	NA	ADC-181
GCA_047772295.1_ASM4777229v 1_genomic	CP179869.1	Ukraine	Ukraine	2023	2023	wound	Skin	15	IC4	NA	2	APH(3'')-Ib # APH(6)-Id # sul2 # floR # mphE # msrE	NA	ADC-263
GCA_047772305.1_ASM4777230v 1_genomic	CP179872.1	USA	United States	2011	2011	Missing	Missing	15	IC4	NA	2	TEM-1 # AAC(3)-IIc # sul2 # APH(6)-Id # APH(3'')-Ib	NA	ADC-263
GCA_024139075.1_ASM2413907v 1_genomic	CP076817.1	Chile:Santiago	Chile	2011	2011	sacrum ulcer	Skin	15	IC4	NA	3	ANT(2'')-Ia # floR # sul2 # OXA-23 # TEM-1 # AAC(3)-IIc	NA	ADC-263
GCA_024139055.1_ASM2413905v 1_genomic	CP076814.1	Chile:Santiago	Chile	2010	2010	tissue	Skin	318	IC4	NA	2	OXA-58	NA	ADC-263
GCA_041154805.1_ASM4115480v 1_genomic	AP031579.1	Nepal:Kathmandu	Nepal	2019	2019	Missing	Missing	15	IC4	NA	0	NA	NA	ADC-263
GCA_047772435.1_AS	CP179875.1	Iraq	Iraq	2003	2003	Hospital environment	Hospital_enviroment	15	IC4	NA	4	ANT(2'')-Ia # CARB-50	NA	ADC-2

M4777243v 1_genomic														
GCA_047772445.1_ASM4777244v1_genomic	CP179880.1	Iraq	Iraq	2003	2003	Hospital environment	Hospital_enviroment	15	IC4	NA	2	ANT(2'')-Ia # CARB-50	NA	ADC-2
GCA_017753625.1_ASM1775362v1_genomic	CP072526.1	China:Hangzhou	China	2019-09-24	2019.7	Missing	Missing	1554	Not IC	NA	3	sul2 # tet(B) # AAC(3)-IId # OXA-58 # sul2 # aadT # msrE # mphE # tetR	NA	ADC-338
GCA_024271825.1_ASM2427182v1_genomic	CP076801.1	Chile:Santiago	Chile	2012	2012	peritoneal liquid	Abdomen	162	Not IC	NA	2	OXA-23	NA	ADC-234
GCA_024138435.1_ASM2413843v1_genomic	CP076804.1	Chile:Santiago	Chile	2011	2011	endotracheal aspirate	Respiratory_tract	162	Not IC	NA	2	OXA-23	NA	ADC-234
GCA_024922185.2_ASM2492218v2_genomic	CP115621.1	USA	United States	2022-08-19	2022.6	sputum	Respiratory_tract	499	Not IC	NA	1	NA	NA	ADC-222
GCA_023615745.2_ASM2361574v2_genomic	CP115623.1	USA	United States	2022-05-13	2022.4	Wound/Abscess	Skin	499	Not IC	NA	2	NA	NA	ADC-222
GCF_023615745.1_ASM2361574v2_genomic	NZ_CP115623.1	USA	United States	2022	2022	Skin	Skin	499	Not IC	NA	2	NA	NA	ADC-222
GCF_024922185.1_ASM2492218v2_genomic	NZ_CP115621.1	USA	United States	2022	2022	Respiratory_tract	Respiratory_tract	499	Not IC	NA	1	NA	NA	ADC-222

GCA_042443055.2_2024CK-01445_genomic	CP174436.1	USA	United States	2024-09-07	2024.7	Wound/Abscess	Skin	499	Not IC	NA	1	NA	NA	ADC-222
GCA_042709415.2_ASM4270941v2_genomic	CP180122.1	USA	United States	2024-09-23	2024.7	Missing	Missing	499	Not IC	NA	1	NA	NA	ADC-222
GCA_041292005.2_2024CK-01227_genomic	CP169592.1	USA	United States	2024-08-05	2024.6	skin	Skin	499	Not IC	NA	1	NA	NA	ADC-222
GCA_042945515.2_ASM4294551v2_genomic	CP180124.1	USA	United States	2024-09-18	2024.7	sputum	Respiratory_tract	499	Not IC	NA	1	NA	NA	ADC-222
GCA_040273095.2_ASM4027309v2_genomic	CP166841.1	USA	United States	2024-06-04	2024.4	skin	Skin	499	Not IC	NA	1	NA	NA	ADC-222
GCA_040273435.2_ASM4027343v2_genomic	CP166843.1	USA	United States	2024-06-04	2024.4	skin	Skin	499	Not IC	NA	1	NA	NA	ADC-222
GCA_044537485.2_ASM4453748v2_genomic	CP180040.1	USA	United States	2024-10-15	2024.8	skin	Skin	499	Not IC	NA	1	NA	NA	ADC-222
GCA_042140325.1_Hv431_genomic	CP169773.1	Missing	Missing	Missing	Missing	Missing	Missing	499	Not IC	NA	2	NA	NA	ADC-222
GCA_050320295.1_Up425_genomic	CP190370.1	USA	United States	2019	2019	urine	Urinary_system	499	Not IC	NA	4	NA	NA	ADC-222
GCA_025853955.1_ASM2585395v1_genomic	CP107583.1	USA	United States	2018-08-14	2018.6	skin	Skin	499	Not IC	NA	1	NA	NA	ADC-222

GCA_025853915.1_ASM2585391v1_genomic	CP107579.1	USA	United States	2018-08-14	2018.6	skin	Skin	499	Not IC	NA	1	NA	NA	ADC-222
GCA_025853935.1_ASM2585393v1_genomic	CP107581.1	USA	United States	2018-08-14	2018.6	skin	Skin	499	Not IC	NA	1	NA	NA	ADC-222
GCA_025854175.1_ASM2585417v1_genomic	CP107608.1	USA	United States	2018-10-14	2018.8	ENVIRONMENT	ENVIRONMENT	499	Not IC	NA	1	NA	NA	ADC-222
GCA_025854235.1_ASM2585423v1_genomic	CP107614.1	USA	United States	2018-08-14	2018.6	ENVIRONMENT	ENVIRONMENT	499	Not IC	NA	1	NA	NA	ADC-222
GCA_025854115.1_ASM2585411v1_genomic	CP107601.1	USA	United States	2018-08-14	2018.6	skin	Skin	499	Not IC	NA	1	NA	NA	ADC-222
GCA_025854055.1_ASM2585405v1_genomic	CP107595.1	USA	United States	2018-08-14	2018.6	wound	Skin	499	Not IC	NA	1	NA	NA	ADC-222
GCA_025854215.1_ASM2585421v1_genomic	CP107612.1	USA	United States	2018-08-14	2018.6	ENVIRONMENT	ENVIRONMENT	499	Not IC	NA	1	NA	NA	ADC-222
GCA_025853895.1_ASM2585389v1_genomic	CP107577.1	USA	United States	2018-08-09	2018.6	Cervical	Cervical	499	Not IC	NA	1	NA	NA	ADC-222
GCA_025854195.1_ASM2585419v1_genomic	CP107610.1	USA	United States	2018-10-14	2018.8	ENVIRONMENT	ENVIRONMENT	499	Not IC	NA	1	NA	NA	ADC-222
GCA_025854135.1_ASM2585413v1_genomic	CP107603.1	USA	United States	2018-08-14	2018.6	skin	Skin	499	Not IC	NA	1	NA	NA	ADC-222

GCA_042445505.2_2024CK-01444_genomic	CP174438.1	USA	United States	2024-09-05	2024.7	Wound/Abscess	Skin	499	Not IC	NA	1	NA	NA	ADC-222
GCA_025854075.1_ASM2585407v1_genomic	CP107597.1	USA	United States	2018-08-14	2018.6	wound	Skin	499	Not IC	NA	1	NA	NA	ADC-222
GCA_025853975.1_ASM2585397v1_genomic	CP107585.1	USA	United States	2018-08-14	2018.6	skin	Skin	new_alleles	NA	NA	1	NA	NA	ADC-222
GCF_022107375.1_ASM2210737v2_genomic	NZ_CP115632.1	USA	United States	2022	2022	Respiratory_tract	Respiratory_tract	499	Not IC	NA	1	NA	NA	ADC-222
GCA_022107375.2_ASM2210737v2_genomic	CP115632.1	USA	United States	2022-01-17	2022.0	sputum	Respiratory_tract	499	Not IC	NA	1	NA	NA	ADC-222
GCA_014880215.2_ASM1488021v2_genomic	CP081137.1	USA	United States	2018	2018	Blood	Blood	499	Not IC	NA	1	OXA-207 # OXA-207	NA	ADC-222
GCA_042141315.1_Hv781_genomic	CP169798.1	USA: St. Louis	United States	2018	2018	respiratory	Respiratory_tract	499	Not IC	NA	2	OXA-24 # OXA-24	NA	ADC-222
GCA_042141115.1_Hv780_genomic	CP169795.1	USA: St. Louis	United States	2018	2018	respiratory	Respiratory_tract	499	Not IC	NA	2	OXA-24	NA	ADC-222
GCA_042141555.1_Hv782_genomic	CP169801.1	USA: St. Louis	United States	2019	2019	respiratory	Respiratory_tract	499	Not IC	NA	3	OXA-24 # OXA-24 # OXA-24	NA	ADC-222
GCA_027558855.1_ASM2755885v1_genomic	CP033876.1	USA	United States	2018-08-08	2018.6	urine	Urinary_system	499	Not IC	NA	0	NA	NA	ADC-222

GCA_042140495.1_Hv640_genomic	CP169782.1	USA	United States	2020	2020	respiratory	Respiratory_tract	499	Not IC	NA	2	NA	NA	ADC-222
GCA_042140485.1_Hv635_genomic	CP169779.1	USA	United States	2020	2020	respiratory	Respiratory_tract	499	Not IC	NA	2	NA	NA	ADC-222
GCA_041801535.1_ASM4180153v1_genomic	CP163382.1	China: Linyi	China	2021-10-30	2021.8	Temperate monsoon climate	Chicken	new_ST	NA	NA	4	NA	NA	ADC-304
GCA_964290795.1_23S01404-6_genomic	OZ197095.1	Netherlands	Netherlands	2023	2023	equine	equine	new_ST	NA	NA	0	NA	NA	ADC-312
GCA_002948925.1_ASM294892v1_genomic	CP026761.1	Missing	Missing	Missing	Missing	Missing	Missing	229	IC3	NA	0	NA	NA	ADC-25
GCA_042146535.1_Lv648_genomic	CP169832.1	USA	United States	2020	2020	respiratory	Respiratory_tract	229	IC3	NA	0	NA	NA	ADC-25
GCA_030585385.1_ASM3058538v1_genomic	CP046554.1	USA: WI	United States	2019	2019	Skin/Wound	Skin	406	Not IC	NA	2	OXA-24 # OXA-24	NA	ADC-170
GCA_030585405.1_ASM3058540v1_genomic	CP046552.1	USA: WI	United States	2019	2019	Skin/Wound	Skin	406	Not IC	NA	1	OXA-24 # OXA-24	NA	ADC-170
GCA_030585345.1_ASM3058534v1_genomic	CP046546.1	USA: WI	United States	2019	2019	Skin/Wound	Skin	406	Not IC	NA	2	OXA-24 # OXA-24	NA	ADC-170
GCA_030585265.1_ASM3058526v1_genomic	CP043458.1	USA: WI	United States	2018	2018	Skin/Wound	Skin	NA	NA	NA	3	OXA-24 # OXA-24	ACICU_RS04610 # ACICU_RS04605 # hemO # ACICU_RS04595 #	ADC-170

													ACICU_RS04590 # ACICU_RS04585 # ACICU_RS04580 # ACICU_RS04575 # ACICU_RS04570 # ACICU_RS04565 # pilT # pilU	
GCA_030585365.1_ASM3058536v1_genomic	CP046549.1	USA: WI	United States	2019	2019	Skin/Wound	Skin	406	Not IC	NA	2	OXA-24 # OXA-24	NA	ADC-170
GCA_036718155.2_ASM3671815v2_genomic	CP149838.1	USA	United States	2024-01-04	2024.0	Wound/Abscess	Skin	2512	Not IC	NA	2	OXA-139	NA	ADC-25
GCF_036718155.1_ASM3671815v2_genomic	NZ_CP149838.1	USA	United States	2024-01-04	2024.0	Wound/Abscess	Skin	2512	Not IC	NA	2	OXA-139	NA	ADC-25
GCA_024749485.1_ASM2474948v1_genomic	CP087317.1	Germany	Germany	Missing	Missing	Missing	Missing	1	IC1	NA	3	APH(3')-Ia # APH(3')-Ia # OXA-58 # OXA-58 # APH(3')-Ia # APH(3')-Ia	NA	ADC-81
GCA_002741415.1_ASM274141v1_genomic	CP024418.1	Greece	Greece	2002	2002	Missing	Missing	1	IC1	NA	1	APH(3')-Ia # OXA-58	NA	ADC-81
GCA_028201495.1_AS	CP116387.1	Australia: Sydney	Australia	1998	1998	wound	Skin	1	IC1	NA	2	NA	NA	ADC-11

M2820149v1_genomic														
GCA_039780165.1_ASM3978016v1_genomic	CP156044.1	Australia: Sydney	Australia	2009	2009	urine	Urinary_system	1	IC1	NA	3	NA	NA	ADC-174
GCA_024749385.1_ASM2474938v1_genomic	CP087304.1	Germany	Germany	Missing	Missing	Missing	Missing	1	IC1	NA	4	NA	NA	ADC-79
GCA_034422775.1_ASM3442277v1_genomic	CP139831.1	Germany	Germany	2017-05-12	2017.4	Missing	Missing	1	IC1	NA	9	APH(3')-VIa # tet(B) # APH(6)-Id # APH(3'')-Ib # msrE # mphE # OXA-97 # msrE # mphE # sul1 # APH(3')-Ia # AAC(3)-Ia # aadA # qacEdelta1 # sul1 # tetR	NA	ADC-176
GCA_042147245.1_Rp376_genomic	CP169833.1	Greece	Greece	2017	2017	Urinary source	Urinary_system	1	IC1	NA	2	APH(3')-VIa # OXA-23	NA	ADC-175
GCA_025677625.1_ASM2567762v1_genomic	CP106943.1	Greece	Greece	2017	2017	urine	Urinary_system	1	IC1	NA	3	APH(3')-VIa # OXA-23	NA	ADC-175
GCA_003296225.1_ASM329622v1_genomic	CP030106.1	Germany	Germany	Missing	Missing	Tracheal secretion	Respiratory_tract	1	IC1	NA	3	APH(3')-VIa # OXA-164	NA	ADC-186

GCA_002999195.2_ASM299919v2_genomic	CP027246.2	China: Sichuan, Chengdu	China	Missing	Missing	Missing	Missing	20	IC1	NA	3	AAC(3)-IId # OXA-58 # msrE # mphE # floR	NA	ADC-79
GCA_029168475.1_ASM2916847v1_genomic	CP119232.1	Taiwan	Taiwan	2001	2001	blood	Blood	1	IC1	NA	3	NA	NA	ADC-185
GCA_003522845.1_ASM352284v1_genomic	CP023029.1	Mexico: Baja California	Mexico	2010-06-04	2010.4	Bronchial fluid	Respiratory_tract	1	IC1	NA	1	APH(3')-VIa	NA	ADC-5
GCA_024139095.1_ASM2413909v1_genomic	CP076821.1	Chile:Santiago	Chile	2011	2011	catheter tip	Urinary_system	1	IC1	NA	1	TEM-1 # AAC(3)-IIc	NA	ADC-263
GCA_046097195.1_ASM4609719v1_genomic	CP175642.1	Australia: Melbourne	Australia	2002	2002	Missing	Missing	1	IC1	NA	2	APH(3')-VIa	NA	ADC-184
GCA_006385075.1_ASM638507v1_genomic	CP041035.1	United Kingdom: Nottingham	United Kingdom	2015	2015	Missing	Missing	1	IC1	NA	0	NA	NA	ADC-184
GCA_022459415.1_ASM2245941v1_genomic	CP091367.1	Belgium	Belgium	Missing	Missing	Missing	Missing	1	IC1	NA	0	NA	NA	ADC-75
GCA_016919505.2_ASM1691950v2_genomic	CP070362.2	Belgium	Belgium	2008	2008	Missing	Missing	1	IC1	NA	3	sul1 # qacEdelta1 # dfrA7 # AAC(6')-Ib3 # GES-11 # APH(6)-Id # APH(3'')-Ib # aadA3 # cmlA5 # ANT(2'')-Ia	NA	ADC-11

												# APH(3')-VIa		
GCA_028583485.1_ASM2858348v1_genomic	CP113080.1	Missing	Missing	Missing	Missing	Missing	Missing	1	IC1	NA	2	ANT(2'')-Ia # cmlA5 # aadA3 # APH(3'')-Ib # APH(6)-Id # GES-11 # AAC(6')-Ib3 # dfrA7 # qacEdelta1 # sul1 # APH(3')-VIa	NA	ADC-11
GCA_028583505.1_ASM2858350v1_genomic	CP113078.1	USA	United States	2008	2008	Tibia, osteotomy elitis	Bone	1	IC1	NA	1	ANT(2'')-Ia # aadA2 # APH(3'')-Ib # APH(6)-Id # GES-11 # AAC(6')-Ib10 # dfrA7 # qacEdelta1 # sul1 # APH(3')-VIa	NA	ADC-11
GCA_000963815.1_ASM96381v1_genomic	CP008706.1	USA: Walter Reed Army Medical Center	United States	2008	2008	tibia/osteomyelitis of diabetes patient	Bone	1	IC1	NA	3	sul1 # qacEdelta1 # dfrA7 # AAC(6')-Ib3 # GES-11 # APH(6)-Id # APH(3'')-Ib # aadA3 # cmlA5 # ANT(2'')-Ia	NA	ADC-11

												# APH(3')-VIa		
GCF_016919525.1_ASM1691952v2_genomic	NZ_CP070358.1	Belgium	Belgium	2008	2008	Human_related	Human_related	1	IC1	NA	3	APH(3')-VIa # ANT(2'')-Ia # cmlA5 # aadA3 # APH(3'')-Ib # APH(6)-Id # ANT(2'')-Ia # cmlA5 # aadA3 # APH(3'')-Ib # APH(6)-Id # GES-11 # AAC(6')-Ib3 # dfrA7 # sul1	NA	ADC-11
GCA_016919525.2_ASM1691952v2_genomic	CP070358.2	Belgium	Belgium	2008	2008	Missing	Missing	1	IC1	NA	3	APH(3')-VIa # ANT(2'')-Ia # cmlA5 # aadA3 # APH(3'')-Ib # APH(6)-Id # ANT(2'')-Ia # cmlA5 # aadA3 # APH(3'')-Ib # APH(6)-Id # GES-11 # AAC(6')-Ib3 # dfrA7 # sul1	NA	ADC-11

GCA_036602705.1_AB5075_UoB_gtr52_SAba13_1.0_genomic	CP144563.1	United Kingdom: University of Birmingham, School of Biosciences	United Kingdom	2021	2021	Agar plate	Lab	1	IC1	NA	2	NA	NA	ADC-11
GCA_036601155.1_AB5075_UoB_1.0_genomic	CP144559.1	USA: Walter Reed Army Medical Center	United States	2008	2008	tibia/osteomyelitis of diabetes patient	Bone	1	IC1	NA	3	aadA3 # cmlA5 # ANT(2'')-Ia # APH(6)-Id	NA	ADC-11
GCA_900478145.1_34555_D01_genomic	LS483472.1	United Kingdom	United Kingdom	2004	2004	Missing	Missing	1	IC1	NA	0	NA	NA	ADC-176 # ADC-11
GCA_028370175.1_ASM2837017v1_genomic	CP116680.1	Canada: British Columbia	Canada	2007	2007	nosocomial spread of war-related MDR-ABC in a Canadian civilian hospital with origin from soldier evacuated via Landstuhl Regional Medical Center	Hospital_enviroment	1	IC1	NA	1	NA	NA	ADC-176 # ADC-11
GCA_013403505.1_ASM1340350v1_genomic	CP058625.1	Canada	Canada	2006-06-30	2006.5	sputum	Respiratory_tract	1	IC1	NA	1	NA	NA	ADC-176 # ADC-11
GCA_016127515.1_AS	CP066016.1	USA:VA	United States	Missing	Missing	Missing	Missing	1	IC1	NA	1	NA	NA	ADC-176 # ADC-11

M1612751v1_genomic														
GCA_000021245.2_ASM2124v2_genomic	CP001182.2	Missing	Missing	Missing	Missing	Missing	Missing	1	IC1	NA	1	NA	NA	ADC-176 # ADC-11
GCA_046531805.1_ASM4653180v1_genomic	CP157212.1	Pakistan: Karachi, Dist. Malir	Pakistan	2023-03-07	2023.2	Wound exudate	Skin	1	IC1	NA	0	NA	NA	ADC-11
GCA_000794125.2_ASM79412v2_genomic	CP114381.1	Australia	Australia	2002	2002	Human sputum	Respiratory_tract	1	IC1	NA	3	NA	NA	ADC-176
GCA_002210065.1_ASM221006v1_genomic	CP021782.1	Australia: Sydney	Australia	2003	2003	sputum	Respiratory_tract	1	IC1	NA	5	OXA-23	NA	ADC-176 # ADC-11
GCA_040834015.1_ASM4083401v1_genomic	CP162145.1	Singapore	Singapore	2010-05-05	2010.3	Missing	Missing	1	IC1	NA	4	APH(3')-VIa # OXA-23	NA	ADC-176 # ADC-11
GCA_045289605.1_ASM4528960v1_genomic	CP142667.1	South Korea	Korea, Republic of	2020-06-07	2020.4	human	human	1	IC1	NA	8	OXA-23	NA	ADC-176 # ADC-11
GCA_036320755.1_ASM3632075v1_genomic	CP143262.1	South Korea	Korea, Republic of	2020-02-11	2020.1	Missing	Missing	1	IC1	NA	1	NA	NA	ADC-176 # ADC-11
GCA_045689785.1_ASM4568978v1_genomic	CP146812.1	South Korea	Korea, Republic of	2020-12-17	2020.9	Missing	Missing	1	IC1	NA	13	OXA-23	NA	ADC-267 # ADC-176
GCA_002082825.1_ASM208282v1_genomic	CP020595.1	South Korea: Seoul	Korea, Republic of	2013-10	2013.8	sputum	Respiratory_tract	1	IC1	NA	1	APH(3')-VIa # OXA-23	NA	ADC-176 # ADC-11
GCA_039545565.1_NCS	CP154372.1	Viet Nam	Viet Nam	2007-04-11	2007.3	Carriage	Carriage	1	IC1	NA	5	OXA-23	NA	ADC-11

R_106_Hybrid_assembly_genomic														
GCA_039779435.1_ASM3977943v1_genomic	CP156041.1	Australia: Sydney	Australia	2010	2010	Missing	Missing	1	IC1	NA	2	OXA-58 # APH(3')-VIa # APH(3')-VIa # APH(3')-VIa	NA	ADC-11
GCA_022467655.1_ASM2246765v1_genomic	CP091340.1	Belgium	Belgium	Missing	Missing	Missing	Missing	1	IC1	NA	0	NA	NA	ADC-267
GCA_022467535.1_ASM2246753v1_genomic	CP091339.1	Belgium	Belgium	Missing	Missing	Missing	Missing	1	IC1	NA	0	NA	NA	ADC-267
GCA_022467375.2_ASM2246737v2_genomic	CP091337.1	Belgium	Belgium	2017	2017	Missing	Missing	1	IC1	NA	1	NA	NA	ADC-267
GCA_001399655.1_ASM139965v1_genomic	CP012952.1	Australia: Sydney	Australia	2008	2008	wound	Skin	81	IC1	NA	4	sul2 # APH(3')-Ia # ANT(2'')-Ia	NA	ADC-240
GCA_042140575.1_Hv766_genomic	CP169788.1	France: Brest	France	2018	2018	respiratory	Respiratory_tract	new_alleles	NA	NA	2	sul1 # qacEdelta1 # dfrA7 # AAC(6')-Ib3 # GES-11	NA	ADC-11
GCA_018831485.1_ASM1883148v1_genomic	CP049314.1	Lebanon	Lebanon	2012	2012	Water (artesian well)	Water	1	IC1	NA	1	NA	NA	ADC-11
GCA_043042805.1_ASM4304280v1_genomic	CP134552.1	China: Jiangxi	China	2010	2010	urine	Urinary_system	1	IC1	NA	0	NA	NA	ADC-30

GCA_002803025.2_ASM280302v2_genomic	CP001172.2	Missing	Missing	Missing	Missing	Missing	Missing	1	IC1	NA	0	NA	NA	ADC-11
GCA_018831465.1_ASM1883146v1_genomic	CP049240.1	Lebanon	Lebanon	2015	2015	Animal (cat)	Cat	1	IC1	NA	0	NA	NA	ADC-11
GCA_012935005.1_ASM1293500v1_genomic	CP050403.1	India	India	2019	2019	sputum	Respiratory_tract	1	IC1	NA	6	OXA-23 # mphE # msrE # APH(3')-VIa # ANT(3'')-IIa	NA	ADC-186
GCA_009428985.1_ASM942898v1_genomic	CP045541.1	India	India	Missing	Missing	Missing	Missing	623	IC1	NA	0	NA	NA	ADC-186
GCA_000069245.1_ASM6924v1_genomic	CU459141.1	Missing	Missing	Missing	Missing	Missing	Missing	1	IC1	NA	4	NA	NA	ADC-11
GCA_000830055.1_ASM83005v1_genomic	CP010781.1	United Kingdom: Nottingham	United Kingdom	1982	1982	Missing	Missing	1	IC1	NA	1	NA	NA	ADC-11
GCA_047227755.2_A297_complete_genome_genomic	CP178354.2	Netherlands	Netherlands	1984	1984	Missing	Missing	1	IC1	NA	3	ANT(2'')-Ia # sul2 # APH(6)-Id # APH(3'')-Ib	NA	ADC-11
GCA_019458485.1_ASM1945848v1_genomic	CP080452.1	USA	United States	2010-06	2010.4	Hip	Hip	1	IC1	NA	4	NA	NA	ADC-191
GCA_021609965.1_ASM2160996v1_genomic	CP091172.1	USA	United States	2010-06	2010.4	Hip	Hip	1	IC1	NA	0	NA	NA	ADC-191

GCA_021484905.1_ASM2148490v1_genomic	CP090607.1	USA	United States	2010-06	2010.4	Hip	Hip	1	IC1	NA	0	NA	NA	ADC-191
GCA_021484925.1_ASM2148492v1_genomic	CP090606.1	USA	United States	2021	2021	Laboratory passage	Lab	1	IC1	NA	0	NA	NA	ADC-191
GCA_002996805.1_ASM299680v1_genomic	CP027528.1	Missing	Missing	Missing	Missing	Missing	Missing	1	IC1	NA	1	NA	NA	ADC-275
GCA_041154875.1_ASM4115487v1_genomic	AP031588.1	Nepal:Kathmandu	Nepal	2020	2020	Missing	Missing	1	IC1	NA	0	NA	NA	ADC-191
GCA_041154775.1_ASM4115477v1_genomic	AP031576.1	Nepal:Kathmandu	Nepal	2019	2019	Missing	Missing	1	IC1	NA	0	NA	NA	ADC-191
GCA_021019455.3_ASM2101945v3_genomic	CP130627.2	Afghanistan	Afghanistan	2018	2018	wound	Skin	1	IC1	NA	0	NA	NA	ADC-191
GCA_021019495.3_ASM2101949v3_genomic	CP130628.2	Afghanistan	Afghanistan	2018	2018	wound	Skin	1	IC1	NA	0	NA	NA	ADC-191
